# Genome wide population genetics and molecular surveillance of insecticide resistance in *Anopheles stephensi* mosquitoes from Awash Sebat Kilo in Ethiopia

**DOI:** 10.1038/s41598-025-95814-0

**Published:** 2025-05-12

**Authors:** Holly Acford-Palmer, Fitsum G. Tadesse, Emilia Manko, Jody E. Phelan, Matthew Higgins, Ashley Osborne, Mojca Kristan, Thomas Walker, Teun Bousema, Louisa A. Messenger, Taane G. Clark, Susana Campino

**Affiliations:** 1 Faculty of Infectious and Tropical Diseases, London School of Hygiene and Tropical Medicine, London, UK; 2 Malaria and NTD Directorate, Armauer Hansen Research Institute, ALERT Hospital Compound, Addis Ababa, Ethiopia; 3 School of Life Sciences, Gibbet Hill Campus, University of Warwick, Coventry, CV4 7AL UK; 4 Department of Medical Microbiology, Radboud University Nijmegen Medical Centre, Nijmegen, The Netherlands; 5 Department of Environmental and Occupational Health, School of Public Health, University of Nevada, Las Vegas, Las Vegas, USA; 6 Parasitology and Vector Biology (PARAVEC), School of Public Health, University of Nevada, Las Vegas, NV USA; 7 Faculty of Epidemiology and Population Health, London School of Hygiene and Tropical Medicine, London, UK

**Keywords:** Population genetics, Ecological genetics

## Abstract

Since the detection of the Asian mosquito *Anopheles stephensi* in Dijbouti in 2012, it has spread throughout the Horn of Africa. This invasive vector continues to expand across the continent and is a significant threat to malaria control programs. Vector control methods, including insecticide-treated nets and indoor residual spraying, have substantially reduced the malaria burden. However, the increasing prevalence of mosquitoes resistant to insecticides, including *An. stephensi* populations, undermines ongoing malaria elimination efforts. Understanding population structure, gene flow between populations, and the distribution of insecticide resistance mutations is essential for guiding effective malaria control strategies. Here, we generated whole genome sequencing data for *An. stephensi* sourced from Awash Sebat Kilo, Ethiopia (*n* = 27) and compared with South Asian populations (*n* = 45; India and Pakistan) to assess genomic diversity, population structure, and uncovering insecticide resistance mutations. Population structure analysis using genome-wide single nucleotide polymorphisms (*n* = 15,533,476) revealed Ethiopian isolates clustering as a distinct ancestral group, separate from South Asian isolates. Three insecticide resistance-associated SNPs (*gaba* gene: A296S and V327I; *vgsc* L1014F) were detected. Evidence of ongoing selection was found in several loci, including genes previously associated with neonicotinoids, ivermectin, DDT, and pyrethroid resistance. This study represents the first whole genome population genetics study of invasive *An. stephensi*, revealing genomic differences from South Asian populations, which can be used for future assessments of vector population dispersal and detection of insecticide resistance mechanisms.

**Supplementary Information:**

The online version contains supplementary material available at 10.1038/s41598-025-95814-0.

## Introduction

The Asian malaria vector, *Anopheles (An.) stephensi*, was first detected in Africa in Djibouti in 2012^1^. Since then, this mosquito species has spread throughout the Horn of Africa (HOA), including Somalia, Ethiopia, Eritrea, South Sudan, Kenya, and with recent reports in Nigeria and Ghana^2–7^. Historically, *An. stephensi* was distributed across South Asia, the Persian Gulf, and the Arabian Peninsula. It is a primary vector of human malaria in India and Pakistan, proficiently transmitting both *Plasmodium falciparum* and *P. vivax*^8,9^. Unlike many traditional malaria vectors, *An. stephensi* is well-adapted to urban environments. Its spread into the HOA has coincided with a surge in malaria cases and urban outbreaks. In Djibouti, *An. stephensi*’s emergence has been epidemiologically linked to a dramatic resurgence in malaria cases, which increased 35-fold between the years 2013 and 2021^10,11^. In Ethiopia, the role of *An. stephensi* in regional urban malaria transmission was confirmed in a recent outbreak of *P. falciparum* in Dire Diwa^12^.

This species is filling a currently empty ecological niche in urban areas in Africa, breeding in both small artificial water sources and natural aquatic habitats near human dwellings^13–15^. This adaptability allows *An. stephensi* to thrive in both rural and urban environments, unlike the primary malaria vectors in the HOA - *An. arabiensis*, *An. gambiae s.s*, and *An. funestus s.s*,- which typically occupy rural areas or regions of high agricultural activity. The ongoing urbanization across Africa and the expanding geographical range *of An. stephensi* is estimated to put a further 120 million Africans at risk from malaria^6,14,16^. With the World Health Organization’s (WHO’s) target to reduce the global malaria burden by 90% by 2030, the need to control this vector has never been more crucial^17^.

Enhancing our understanding of how *An. stephensi* became so well-established in the HOA is essential for predicting its potential spread to new regions. Population genetics has been widely applied to vector species to gain insights into population structure, ongoing selection, and gene flow^18,19^. For *An. stephensi*, studying population structure and genomic architecture can help elucidate its invasion routes and any adaptive evolution that may have occurred since its introduction. Alongside gene flow, selection is of particular interest, as it can reveal alleles associated with insecticide resistance and illustrate how these alleles are spreading through populations^20^.

In Africa, malaria vector control relies on long-lasting insecticide-treated nets (LLINs) and indoor residual spraying (IRS). There are five main classes of insecticides in use: pyrethroids, organochlorides, organophosphates, carbamates, and pyrroles, each with varying modes of action. However, resistance to all major adulticide classes has been reported across many *Anopheles* species, including *An. stephensi* across the HOA, India, Pakistan, Sri Lanka, and the WHO Eastern Mediterranean region^3,21–23^. Resistance to insecticides can arise through multiple mechanisms: target site resistance, overexpression of metabolic enzymes, behavioural changes, microbiome alterations, and thickening of the insect cuticle^24–26^. Predominantly, resistance results from metabolic or target site alterations. Target-site resistance arises from single nucleotide polymorphisms (SNPs) that alter the target protein’s amino acid sequence and result in conformational changes that prevent the insecticide from binding properly^25^. The target site mutation A296S in the *gaba* receptor has been reported in *An. stephensi* from Ethiopia, and is associated with resistance to dieldrin (*rdl)*, an organochlorine banned in the 1990s due to concerns about its environmental persistence and potential impact on human health^27–29^. The L1014 knockdown resistance (*kdr*) mutation in the *voltage-gated sodium channel (vgsc*) is linked to cross-resistance to Dichlorodiphenyltrichloroethane (DDT) and pyrethroids and has been reported in *An. stephensi* from Ethiopia, Afghanistan, and India^28,30–33^. However, there have been examples of pyrethroid resistance in *An. stephensi* in the absence of this mutation, implying other mechanisms may contribute to the development of resistance^23,34^. These alternative mechanisms could include metabolically mediated resistance, characterized by increased detoxification of insecticides through the overexpression of *glutathione s-transferases* or *cytochrome P450s*, thereby reducing insecticide efficacy^24,25^. The genomic changes leading to metabolic resistance are generally more complex than those associated with target-site resistance. In some cases, missense SNPs increase insecticide metabolism, such as the L119F mutation in the *GSTe2* gene, which causes cross-resistance to pyrethroids and DDT in *An. funestus*^35^. Other, more complex mechanisms include structural variants or copy number variations, like the 6.5 kb insertion that mediates pyrethroid resistance, also observed in *An. funestus*^6^. These examples underscore the importance of looking beyond simple SNPs and small insertions or deletions (INDELs) to fully understand the molecular mechanisms underlying insecticide resistance.

To date, the detection of insecticide resistance markers and population genetics of the invasive *An. stephensi* species has been limited to the examination of a few candidate genes. This species emergence in Africa is not fully understood, but one hypothesis is that *An. stephensi* was transported through either human mediated travel or cattle transportation^18,36,37^. Other theories include long-distance wind-borne migration^38^. The Ethiopian isolates used in this study were collected in Awash Sebat Kilo, a town 200 km east of Addis Ababa and a main transportation corridor from Addis Ababa to Djibouti. Awash Sebat Kilo is an area of high malaria transmission and with large populations of *An. stephensi* with reports of insecticide resistance, making it an important site for investigating the population genetics of *An. stephensi*^8,18,21,40^.

The application of whole genome sequencing (WGS) provides a holistic view of the genomic landscape, allowing for insights into mosquito ancestry, ongoing genetic selection, and the identification of insecticide resistance markers. The availability of increasingly affordable and high throughput platforms for WGS is resulting in a growing number of *Anopheles* species with available genomic data^19,40,41^. Previous population genetics studies of *An. stephensi* from Ethiopia have either investigated limited gene numbers or used double digest restriction-site associated DNA sequencing (ddRADseq). While these approaches yielded valuable insights into insecticide resistance and population structure, they provided only a partial view of the genome^18,21,40^. Here, we generate the first whole genome sequence data from *An. stephensi* collected in Africa, specifically from Awash Sebat Kilo, Ethiopia. We conduct a population genetic analysis of these Ethiopian *An. stephensi* WGS data (*n* = 27), alongside publicly available data sourced from South Asia (India and Pakistan, *n* = 45) to gain a deeper understanding of the genomic architecture of this vector, to gain insight into its ancestry, phylogenetics, and uncover loci under selective pressure.

## Results

### Whole genome sequence data and nucleotide diversity

Whole genome data was generated from 27 mosquitoes sourced from Awash Sebat Kilo, Ethiopia, in 2019. The number of raw sequenced paired reads across the 27 samples ranged between 26,841,458 and 85,763,451. After mapping to *An. stephensi*’s three chromosomes, this resulted in an average coverage of 33.7-fold (standard deviation 7.5-fold). A further 45 isolates with publicly available WGS data from India (Bangalore and Mangalore, *N* = 21; insectary colonies, *N* = 16) and Pakistan (insectary colony, *N* = 8) were also mapped, and across the combined dataset (*N* = 72), 15,533,476 SNPs passed filtering criteria (details in Methods). Sliding window analysis (size 100kbp) revealed low nucleotide diversity with π averaging < 0.03 across *An. stephensi*’s three chromosomes (Supplementary Fig. S1 and Supplementary Table S1).

### Population differentiation and ancestral analysis reveals distinct geographic groups

**Fig. 1:**
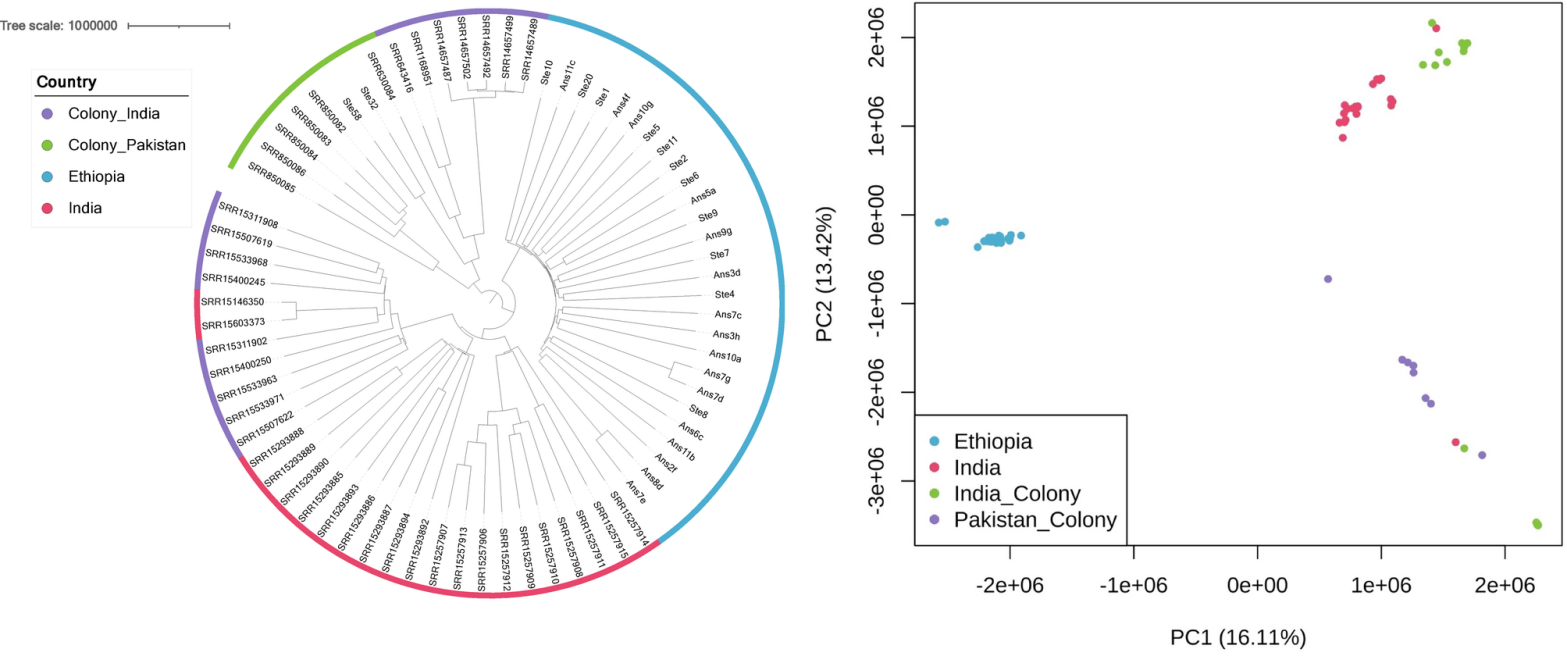
Neighbourhood joining tree and principal component analysis plot generated from pairwise distance matrix of available *An. stephensi* WGS isolates. (Ethiopia (*n* = 27), Indian colony (*n* = 21), Indian field(*n* = 16), and Pakistan colony (*n* = 8)).

Using the 15,533,476 SNPs, both principal component and phylogenetic analysis revealed that the Ethiopian, Pakistan colony, and Indian field *An. stephensi* isolates formed distinct genetic clusters (Fig. 1). The Indian field isolates also clustered with a subset of Indian colony mosquitoes, whilst the remaining (*n* = 5) Indian colony isolates appear genetically closer to the Pakistani SDA500 colony isolates. This separation is based on their origin, the five clustering with Pakistani colony samples are the Walter Reed colony strain, and those appearing with the Indian field isolates are a different laboratory-reared colony strain. Application of the pairwise F_ST_ population differentiation metric, which quantifies differences in allele frequencies, confirmed that the greatest genetic distinctness was between Ethiopian field and Pakistan insectary colony samples (70,898 SNPs with F_ST_ > 0.8; 3,472 SNPs with F_ST_ = 1, average F_ST_ = 0.133) (Table 1). Indian field and colony samples were the most genetically similar (2,411 SNPs with F_ST_ > 0.8, 354 SNPs with F_ST_ =1, average F_ST_ = 0.073) (Supplementary Table S2).

**Table 1:** Number of SNPs with significant pairwise F_ST_ calculations.

Populations	F_ST_ > 0.8	F_ST_ = 1.0	Average F_ST_
Ethiopia vs. India field	7539	194	0.081
Ethiopia vs. India Colony	30,567	938	0.120
Ethiopia vs. Pakistan Colony	70,898	3,472	0.133
India field vs. India Colony	2411	355	0.073
India field vs. Pakistan Colony	35,077	2,942	0.103
India Colony vs. Pakistan Colony	34,479	3,390	0.117

**Fig. 2:**
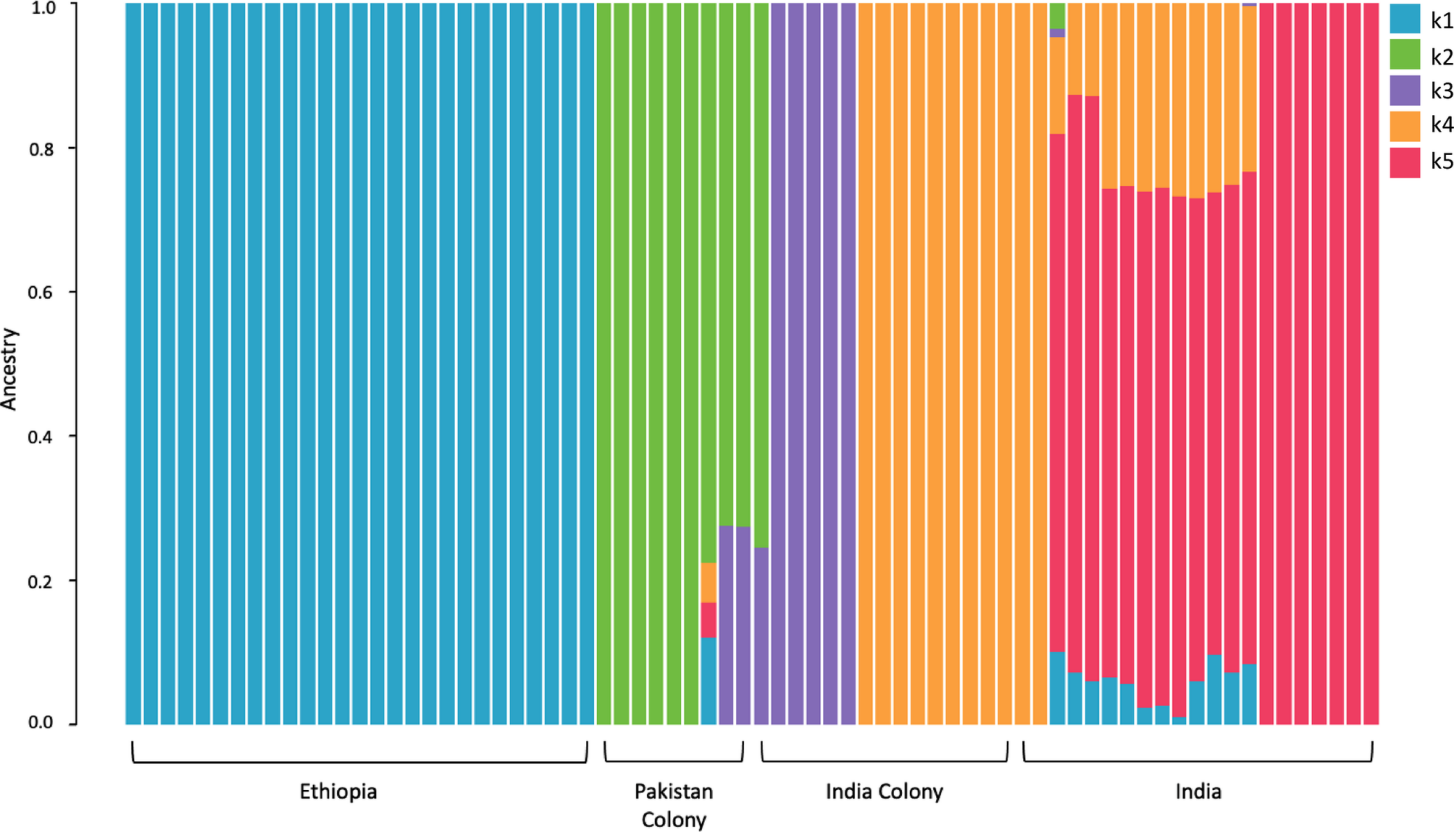
Genome wide admixture analysis of *An. stephensi* isolates. Each isolate is represented by a column. Five ancestral populations were identified using ADMIXTURE (K = 5 ancestral populations, labelled k1-k5), across the four isolate groups (Ethiopia (*n* = 27), Indian colony (*n* = 21), Indian field (*n* = 16), and Pakistan colony (*n* = 8)) analyses.

Admixture analysis was performed to identify any potential ancestral relationships between the isolates and revealed five ancestral populations (labelled, k1-k5): Ethiopian, Indian field, Pakistani colony, and the separation of Indian colony isolates, as observed in the phylogenetic tree. The Ethiopian isolates consisted of one sub-population and the same ancestral population was also observed in a sample from Pakistan colony and Indian field isolates (Admixture plots K = 2, K = 3, K = 4 in Supplementary Fig. **S2**, and K = 5 in Fig. 2). In the Indian field isolates, the k4 ancestral sub-population was dominant, and no clear distinction could be seen between Bangalore and Mangalore isolates (Fig. 2**)**. The k3 ancestral sub-population appeared as the only ancestry present in a subset of Indian colony samples. In the remaining Indian colony samples, k5 was dominant, although two isolates shared the k2 ancestral sub-population that was predominant in colony samples from Pakistan. The single Pakistani colony sample sharing ancestry with Ethiopia also had k3 and k5 ancestral types seen in Indian field and colony samples(Fig. 2).

### Selection analysis reveals genes involved with insecticide resistance

Genome-wide selection scans were performed to identify signals of recent positive selection using the Cross-Population Extended Haplotype Homozygosity (XP-EHH) method between populations and integrated Haplotype Score (iHS) within each population. Using XP-EHH, 275 SNPs were identified as having ongoing directional selection (XP-EHH > 4.0), with 148 SNPs within coding sequences (CDS) of 81 genes, and 127 SNPs within introns (Supplementary Table S3). One gene was identified as a candidate potentially involved in insecticide resistance (*acetylcholine receptor (nAChR) subunit beta-like 2*) (two SNPs, XP-EHH values > 5.3) in a comparison of Indian and Ethiopian field samples (Table 2). In single population (iHS) analysis, a total of 997 loci were identified as having significant iHS scores (iHS > 4.0), 439 were within non-coding regions, and the remaining 559 occurred within CDS across 325 different genes (Supplementary Table S4). Of these, five were candidate genes that had loci with iHS scores indicative of selection (iHS > 4.0), including the *nAChR subunit beta-like* 2 in the Indian field isolates. The remaining four genes were *nAChR alpha-like* (Ethiopia and Indian field samples), glutamate-gated chloride channel (Pakistani Colony), *cytochrome P450 307a1*-like (*CYP307a1*) (Indian field isolates), and *gaba* subunit beta (India field samples and Pakistan colony).

**Table 2:** Genes identified as undergoing directional selection with either XP-EHH or iHS statistics.

Chromosome	Position	Score	Gene	Populations
XP-EHH				
NC_050202.1	68,720,664	5.30439559	acetylcholine receptor subunit beta-like 2	Ethiopia|India field
NC_050202.1	68,720,666	5.30439559	acetylcholine receptor subunit beta-like 2	Ethiopia|India field
iHS				
NC_050201.1	13,607,505	4.157532377	gamma-aminobutyric acid receptor subunit beta-like	Pakistan colony
NC_050202.1	68,736,715	4.16320592	acetylcholine receptor subunit beta-like 2	India field
NC_050202.1	68,736,717	4.24447916	acetylcholine receptor subunit beta-like 2	India field
NC_050202.1	68,789,775	-4.5335888	acetylcholine receptor subunit alpha-like	India field
NC_050202.1	68,790,027	-4.2056141	acetylcholine receptor subunit alpha-like	India field
NC_050202.1	68,790,037	-4.5254523	acetylcholine receptor subunit alpha-like	India field
NC_050202.1	68,790,043	-4.5118037	acetylcholine receptor subunit alpha-like	India field
NC_050202.1	68,874,338	3.97023238	acetylcholine receptor subunit alpha-like	Ethiopia
NC_050203.1	8,347,167	4.26288047	gamma-aminobutyric acid receptor subunit beta	India field
NC_050203.1	20,668,384	3.94395547	glutamate-gated chloride channel	Pakistan colony
NC_050203.1	20,668,385	3.94395547	glutamate-gated chloride channel	Pakistan colony
NC_050203.1	67,922,600	4.0921626	cytochrome P450 307a1-like	India field
NC_050203.1	67,925,612	3.91798343	cytochrome P450 307a1-like	India field
NC_050203.1	67,925,620	3.92503232	cytochrome P450 307a1-like	India field
NC_050203.1	67,925,893	4.3089353	cytochrome P450 307a1-like	India field
NC_050203.1	67,926,103	4.25137268	cytochrome P450 307a1-like	India field
NC_050203.1	67,926,118	4.03362144	cytochrome P450 307a1-like	India field
NC_050203.1	67,926,235	4.11747932	cytochrome P450 307a1-like	India field

### Identification of three insecticide resistance associated SNPs

A total of 25 missense SNPs were identified across four genes previously associated with insecticide resistance in *Anopheles* mosquitoes (*ace-1*, *gaba* (*rdl*), *GSTe2*, and *vgsc*) (Table 3). Three of these are SNPs known to be associated with target-site mediated resistance (*vgsc* L1014F; *gaba* A296S and V327I). The *vgsc* L1014F SNP mediates resistance to pyrethroids and was identified exclusively in the Ethiopian population. It was only identified as a heterozygous genotype in 2 samples (allelic frequency of 7.4% (2/27 samples)). The *gaba* A296S mutation was found in all four populations (allelic frequency of 18.8%; 27/72 samples), with all samples being heterozygous at this position, no homozygotes were found for this mutation. The V327I mutation was only present in the Indian field and colony populations (5 samples). This SNP was also present as the heterozygous genotype and only in samples that also had the *gaba* A296S mutation. A further 22 missense mutations were identified across the four key insecticide resistance-associated genes (*ace-1 (n =* 5), *gaba* (*n =* 9), *GSTe2 (n =* 9), *vgsc* (*n* = 2)), with allelic frequencies ranging from 1 to 83% (Table 3). No nonsense or nonstop mutations were detected in these genes.

**Table 3:** Missense SNPs identified across four main insecticide resistance associated genes.

Chromosome	Position	Amino acid alteration	Populations*	Allele frequency		Genotype frequency		
				Reference (*R*)	Alternative (A)	*R*/*R*	*R*/A	A/A
NC_050202.1	60,913,808	V189I	E, IC & IF	0.91	0.09	0.83	0.16	0.01
NC_050202.1	60,913,844	N177D	All	0.17	0.83	0.07	0.21	0.72
NC_050202.1	60,916,071	G94S	All	0.22	0.78	0.07	0.31	0.63
NC_050202.1	60,916,173	G60S	PC	0.99	0.01	0.99	0.01	0.00
NC_050202.1	60,916,175	V59A	All	0.24	0.76	0.13	0.22	0.65
NC_050203.1	8,349,210	M349I	PC	0.99	0.01	0.99	0.01	0.00
NC_050203.1	8,352,962	V327I	IC & IF	0.97	0.03	0.93	0.07	0.00
NC_050203.1	8,353,055	A296S	All	0.81	0.19	0.63	0.36	0.01
NC_050203.1	8,380,167	L101S	IC	0.98	0.02	0.96	0.04	0.00
NC_050203.1	8,391,526	P77A	Ethiopia	0.97	0.03	0.93	0.07	0.00
NC_050203.1	8,391,528	P76Q	IF	0.99	0.01	0.97	0.03	0.00
NC_050203.1	8,391,529	P76T	Ethiopia	0.99	0.01	0.99	0.01	0.00
NC_050203.1	8,395,314	G74E	IC	0.95	0.05	0.90	0.10	0.00
NC_050203.1	8,395,317	Y73C	Ethiopia	0.96	0.04	0.93	0.06	0.01
NC_050203.1	42,808,541	A1955C	Ethiopia	0.96	0.04	0.97	0.03	0.00
NC_050203.1	42,817,709	L958F	Ethiopia	0.99	0.01	0.94	0.06	0.00
NC_050203.1	70,580,273	T222A	E & PC	0.97	0.03	0.90	0.10	0.00
NC_050203.1	70,581,404	F196Y	E & PC	0.95	0.05	0.08	0.06	0.86
NC_050203.1	70,581,440	I184T	IC	0.11	0.89	0.98	0.01	0.01
NC_050203.1	70,581,534	R153C	All	0.98	0.02	0.57	0.26	0.17
NC_050203.1	70,581,555	C146S	All	0.70	0.30	0.08	0.10	0.82
NC_050203.1	70,581,607	N128R	E & PC	0.93	0.07	0.90	0.06	0.04
NC_050203.1	70,581,621	P124T	Ethiopia	0.98	0.02	0.96	0.04	0.00
NC_050203.1	70,581,766	H97N	All	0.21	0.79	0.14	0.14	0.72
NC_050203.1	70,581,858	G66A	All	0.24	0.76	0.18	0.11	0.71

* E = Ethiopia, IC = India colony, IF = India field, PC = Pakistan colony.

### Detection of structural variants: copy number variants and indels

For coverage-based analysis, only one significant deviation from the median genome coverage for the Ethiopian population was observed (Supplementary Figure S3). A possible copy number variant (CNV) was detected on chromosome two in a region containing a cluster of *cytochrome P450* genes (CYP). The *CYP6a* cluster on chromosome two (NC_050202.1) was observed to have elevated coverage in comparison to both the median coverage and other populations.

From analysis with Delly, a structural variant prediction tool, 57,815 deletions were identified after quality control filtering (specified in the method section). Of these, 36 were identified across 18 genes previously identified as involved in insecticide resistance or as belonging to a gene family possibly involved in insecticide metabolism^41^. These included two deletions in *cytochrome P450* genes: a 494 bp intronic deletion in *CYP307a1* was detected in an Ethiopian sample and a frameshift-causing deletion in *CYP6a1*, detected in a single Indian sample (SRR1529388). This deletion was one of two detected in the *CYP6a1* gene, where the other was a 3’ UTR variant found in both Indian colony and field samples. There were 2901 duplications originally found across 44 genes and 25 intergenic regions. Of these, 351 (12.1%) resulted in a frameshift variant. An 181 bp duplication event was identified in the *CYP9f2* candidate gene in one Indian colony sample (SRR1529388, see above) but was annotated as a non-coding transcript. Delly did not detect any Copy Number Variants (CNVs) in the chromosome 2 CYP6a cluster with elevated coverage identified in Supplementary Figure S3.

## Discussion

The invasion of *An. stephensi* into the HOA is a significant threat to malaria control and elimination efforts in sub-Saharan Africa. Understanding the origins of this invasive species and ongoing gene flow can provide greater insight into its emergence in this region and improve predictions of future spread. Here we explore whole genome sequence data of *An. stephensi* and demonstrate, using population structure analysis, that our Ethiopian field isolates from Awash Sebat Kilo, are genetically distinct from Indian and Pakistani insectary colony and field populations. Ancestry analysis indicated that each field population constituted a distinct ancestral population. The Indian field isolates, despite the large geographical distance between the regions (Bengaluru and Mangaluru, around 350 km), shared an ancestral population (K4) in the presence of several minor ones. The Indian colony samples were divided into two groups based on their origin (Walter Reid or laboratory-reared strains). All samples from Ethiopia inherited the k1 ancestry, which was present at a minor proportion in some field Indian samples and a single Pakistan SDA500 colony sample (intermediate form). This observation suggests a possible South Asian origin of the Ethiopian samples, as previously reported, however further comparison with genomic data from other geographical regions is necessary to enhance the accuracy of origin inference^2^. Previous studies using candidate gene analysis have identified high levels of genetic diversity across Ethiopian populations, implying either one large invasion incident or multiple smaller colonisation events^18,37^. The samples analysed here were collected in the same market town, which could have resulted from a population expansion in this area, resulting in the homogenous ancestry observed. The ancestry observed in Ethiopia might have been dominant in other areas in South Asia at the time of the introduction or introductions events. A similar relationship is observed between the Indian insectary-reared and field isolates, where the former was collected in 2016 in Chennai, India and has only k5 ancestral subpopulation. This is shared with 14 Indian field samples with the k1 Ethiopian ancestral subpopulation, suggestive of shared ancestry between the Indian populations, despite the large geographical distance between Chennai, Bengaluru, and Mangaluru. Other studies have indicated that *Anopheles* spp. population structure remains stable over time, and physical distance is a larger driver of genetic variation^42,43^. However, there are also cases where species dynamics change relatively quickly, leading to genomic changes, such as in response to increased insecticide exposure, as well as examples where physical distance and genetic distance deviate, indicative of processes like consecutive founder events^44–47^. It is clear that to better estimate the origins and dispersion of *An. stephensi*, a more comprehensive WGS dataset is needed from these and other native and recently invaded geographical regions, including India, Pakistan, Afghanistan, Saudi Arabia, Iran, as well as elsewhere in the Arabian Peninsula and HOA. One of this study’s key limitations is the abundance of colony samples analysed, which may introduce bias into the results of selection and genetic diversity tests. Colonies such as the Pakistani SDA500 strains have been laboratory reared for decades, and in these conditions, limited gene flow results in reduced genetic diversity, inbreeding, and increased linkage disequilibrium. The local field populations from the regions where the colony samples originated would likely exhibit far greater genetic diversity, which is not represented in the samples used here.

The *kdr* L1014F insecticide resistance mutation was detected at a low frequency in this Ethiopian population and was absent in the Indian field samples. This *kdr* mutation confers resistance to pyrethroids and DDT which has been found previously in Indian populations of *An. stephensi* collected in 2016, and in Afghan populations, collected in 2018^31,32^. The *kdr* L1014F mutation is absent in the Mangalore and Bangalore populations analysed here, despite evidence of extensive pyrethroid resistance in both these cities^3,48^. The low proportion of Ethiopian isolates with this SNP, along with its presence as a heterozygous genotype, implies it has recently arisen in this population. This is supported by a previous work in Awash, where no evidence of the *kdr* mutation was found in samples collected a year prior to those examined here^18^. We have previously identified this *kdr* L1014F mutation at low frequency in a set of samples from the same regions/year when using an amplicon assay^28^. The low allelic frequency of the resistance marker, in the context of phenotypic resistance in the sampling location, indicates mechanisms other than target site modifications are responsible for this observed phenotype.

The other known target site mutation identified in the Ethiopian isolates was *gaba* A296S, which confers resistance to dieldrin^27,29^. This substitution was also identified in the Indian field populations, highlighting its prevalence, despite the insecticide being banned since the 1990s. The presence of this mutation corroborates the historical shared ancestry of the isolates analysed here. The *gaba* V327I mutation was also identified, and has a strong association with the A296S alteration, with five Indian field and colony samples carrying both SNPs^49,50^. A further 22 putatively novel SNPs were identified in genes associated with insecticide resistance. Six of these SNPs appeared in two Pakistani colony samples (Ste32 and Ste58) known to be insecticide susceptible. Two of these SNPs occurred in the *ace-1* gene (N177D and V59A), and four in *GSTe2* (F196Y, C146S, H97A, and G66A). The remaining 16 missense SNPs occur in populations where the resistance status is unknown, so it is not possible to infer any impact on insecticide susceptibility. None of these missense SNPs, including those previously associated with insecticide resistance, had any evidence of ongoing selection using either the iHS or XP-EHH statistics.

Of the genes identified as having ongoing directional selection, five could be potentially involved in insecticide resistance. The *nAChR receptor subunit beta* was found under selection by both within (iHS) and between (XP-EHH) population analysis. The *nAChR receptor subunit alpha* was also identified using the iHS metric as being under ongoing selection in Indian and Ethiopian populations. These two subunits of the receptor are the result of splicing of the *nAChR* gene; the presence (alpha) or absence (beta) of two cysteines determines their type^51^. Mutations within *nAChR* have been reported to result in resistance to neonicotinoids, which are pesticides that mediate synaptic transmission via nAChR, resulting in insect mortality^52,53^. This type of pesticide usage has been reported to result in neonicotinoid resistance in other *Anopheles* species^54^. Worryingly, neonicotinoids are considered an alternative to pyrethroids, for vectors with high levels of resistance to pyrethroids^3,55–57^. In Ethiopia, the President’s Malaria Initiative have been using SumiShield, which contains a neonicotinoid, since 2021. However, this is after the collection of these samples, so it is unknown whether they had been exposed to the chemical^58^. In addition, significant directional selection was detected in glutamate-gated channel genes in the Pakistan colony isolates. Mutations in these loci have been associated with ivermectin resistance in *Drosophila*^59–61^. Ivermectin is an anti-parasitic/endectocide, often used in mass drug administrations, which kills both the *Plasmodium* parasite, and *Anopheles* spp. mosquito when they ingest the blood of a treated host^62–64^. Ivermectin has been trialled as a vector control method using mass drug administration to help reduce malaria cases^65^.

Distinct signals of selection in a *gaba* gene were identified in India field and Pakistan colony samples. Fipronil, an insecticide that targets gaba receptors, induces neurotoxicity, which may be linked to these selection patterns^66^. This phenylpyrazole has also been proposed as part of a One Health approach to vector control^67^. Similarly to ivermectin, fipronil can be used in mass drug administration, particularly in livestock to target zoophilic vectors like *An. stephensi*. This approach has been successfully trialled in several studies^68–70^. Resistance to fipronil has been reported in Iranian *An. stephensi* isolates, with both *kdr* and *rdl* mutations hypothesised to be involved^71^. Mutations within *gaba* receptors have been associated with reduced insecticide efficacy of fipronil, although this has not been observed in *Anopheles spp*^72–74^. Further surveillance of this gene could provide valuable insights into its potential involvement in fipronil resistance.

Another notable gene exhibiting strong directional selection by iHS was *CYP307a1*, identified in the Indian field populations. A 494 bp deletion was detected in the intronic region of this locus in one Ethiopian isolate, and may impact gene expression or result in alternative splicing^75^. *CYP307a1* is a member of the *cytochrome P450* gene family and has previously been linked to resistance to both DDT and pyrethroids in *An. funestus*^76,77^. Similarly, in other insect species (*Cydia pomonella*), the upregulation of *CYP307a1* has been associated with deltamethrin (pyrethroid) resistance^78^. Typically, *CYP307a1* is involved in ecdysteroid hormone biosynthesis, these hormones control mosquito behaviour, nervous system development, and reproduction^79^. This gene has not been confirmed to directly result in insecticide resistance but warrants further investigation.

Other structural variants were detected that resulted in amino acid alterations, including a 67 bp deletion in the *CYP6a1* gene, found in a single Indian colony sample. Additionally, a 3’ UTR variant in the same gene was detected in both Indian colony samples and field samples. Altered expression levels of *CYP6a1* have previously been associated with deltamethrin resistance in *D. melanogaster* and *C. pipiens*^80,81^. With the absence of *kdr* mutations in these Indian field samples, but phenotypic pyrethroid resistance reported near the collection sites, it is likely that other mechanisms contribute to resistance; such as metabolic resistance^3,23,34,82^. This *CYP6a1* gene identified here is not in the *CYP6a* cluster that was observed to have elevated coverage proportional to the genome median in Ethiopian isolates. To further investigate this gene cluster, read orientations and breakpoints would need to be identified to confirm whether this increased coverage was due to a copy number variation in the population.

A limitation of this study was the lack of phenotype data on insecticide response. Further investigations, using WGS or targeted amplicon sequencing, in tandem with susceptibility bioassays, are needed to investigate the impact of these mutations on insecticide response. The novel missense SNPs potentially linked with resistance should be used as targets in high-throughput molecular assays, to support surveillance and assist functional work to understand and validate underlying mechanisms associated with resistant phenotypes.

In conclusion, this study gives greater insight into the population genetics of cross-continental *An. stephensi*. Applications of WGS analysis to larger *An. stephensi* sample cohorts, across different geographical regions, will be key to understanding gene flow and identifying insecticide resistance markers. Such insights will enable public health authorities to make informed choices about vector surveillance and insecticide usage.

## Methods

### Mosquito collection and identification

*An. stephensi* mosquitoes were sourced from an LSHTM colony (Sind Kasur strain, SDA500, originally from Pakistan in 1982). Field samples were collected in Awash Sebat Kilo, Ethiopia (GPS coordinates *9.003009*,* 40.167630)*, between April and September 2019. The study protocol was conducted in accordance with relevant ethical guidelines and regulations, as described in the “Ethical Approval and Consent” section. The samples were collected in one of three ways: CDC mini light traps, aspiration from cattle shelters, and human landing collection. All mosquitoes were identified morphologically as *An. stephensi* before a multiplex qPCR with *ITS2* and *cox-1* genes was used for molecular confirmation^8,83,84^.

### DNA extraction

Mosquitoes were individually suspended in 1X phosphate buffered saline (PBS), before being mechanically lysed with a Tissue Ruptor II (Qiagen, Hilden, Germany) for 30 s, or until all body parts were no longer visible. DNA was extracted using the Qiagen Blood and Tissue kits, according to manufacturer’s instructions. The study protocol was conducted in accordance with relevant ethical guidelines and regulations, more information on this can be found in the “Ethical Approval and Consent” section. DNA concentrations for each sample were quantified using the Qubit 2.0 fluorimeter HS DNA kit (ThermoFisher). DNA was then stored at − 20 °C.

### Whole genome sequencing and bioinformatic analysis

The DNA of 27 *An. stephensi* isolates were sequenced on the Illumina MiSeq using 2 × 250 bp paired end configuration. Twenty-five of these isolates were collected in Awash Sebat Kilo, Ethiopia, and the remaining two were colony mosquitoes. Publicly available *An. stephensi* WGS data was included in the population analyses, this includes 21 field Indian samples (11 from Bangalore, and 10 from Mangalore)^85^. A total of 24 publicly colony samples were included in the analysis, 16 of which were Indian colony samples, and eight Pakistan colony samples. The raw WGS sequence data was first trimmed using trimmomatic software (version 0.39), before being aligned to the UCI_ANSTEP_V1.0 (*An. stephensi*) reference genome, using bwa-mem (version 0.7.17) software (with default parameters)^86–88^. Coverage statistics from the resulting bam files were calculated using samtools (version 1.9)^89^. Variants (SNPs and INDELs) were called and validated using GATK software (version 3.8) with the HaplotypeCaller^90^. Once individual VCF files had been generated, a multi-sample VCF was created using GATK’s GenomicsDBImport and GenotypeGVCFs function. The multi-sample VCF was then filtered to contain only chromosomal variants using bcftools (version 1.9), this package was also used to sort and normalise multi-allelic variant sites. Further filtering was conducted using vcftools (version 0.1.16), removing variants with a depth < 5 and more than 50% missingness^91^. Missingness refers to the percentage of missing data for each SNP site; each SNP must have at least 50% of samples with sufficient depth for a reliable SNP call. A total of 16,580,599 SNPs were initially identified and reduced to 15,533,476 after filtering.

### Identification of insecticide resistance associated SNPs

A bed file (Supplementary Table S6) containing genes associated with insecticide resistance was created based on a literature search. This search included the *para*,* gaba*, and *ace-1* genes associated with target site resistance, along with *cytochrome P450*’s, and esterase’s linked to metabolic-based resistance. The bed file included the 500 bp before the gene start codon, and 500 bp after the stop codon to identify any variants within the promoter or terminator regions. The bed file was then applied to the filtered multi-sample VCF using bcftools. The package snpEff was then used to annotate these variants, using a custom-built database from the UCI_ANSTEP_V1.0 gene feature file in gff format gff^86,92^.

### Population genetic analysis

A pairwise-genetic distance matrix was generated from the filtered multi-sample VCF file, using an in-house script^93^. This distance matrix was used as the basis for the generation of a neighbourhood joining tree, and principal component analyses generated in R using ape and qqman packages^94,95^. The resulting neighbourhood joining (NJ) tree was visualised and annotated using iTOL^96^. ADMIXTURE software (v1.3) was used to conduct admixture analysis^97^. PLINK package (version 1.90b6.21) was first used to convert the VCF file to a bed file for these analyses^98^. The optimum K value (estimated number of ancestral populations) was calculated by cross-validation of 1–10 dimensions of eigenvalue decay (k = 5). This value along with the bed file was used by ADMIXTURE software (version 1.3.0) to identify shared ancestral populations. The output was then visualised in R. To investigate genetic differentiation in *An. stephensi*, Fst was calculated using the Weir and Cockerham estimator between Ethiopian and Indian field populations, using vcftools and visualised in the R statistical tool. Nucleotide diversity was also calculated across the genome, using 100 kb windows. Genomic regions under directional selection, were detected with the R-based package rehh^99^. The Integrated haplotype statistic (iHS) was used to find selection within populations, a positive iHS value indicates selection for the ancestral (reference) allele, whilst a negative iHS score suggests selection for the derived (alternate) allele. Extended haplotype homozygosity (XP-EHH) was used to identify selection ongoing between populations, with a positive score suggesting selection in population A and a negative score indicating population B is under selection. In this instance, a score > 4.0 or <-4.0 is considered significant.

### Identification of structural variants

Two methods were utilised to detect copy number variants for this data set. First, a coverage-based method was used focussing on clusters of *CYP* genes identified in the genome (Supplementary Table S5). Sample coverage was averaged by collection location resulting in four populations: Ethiopia, Indian field, Indian colony, and Pakistani colony. Coverage for each population was normalised using Kernel-smoothing, and then plotted against the median genome coverage depth for that population.

Secondly, Delly software (version 0.7.6) was used to identify structural variants (SVs) > 60bp^100^. Delly uses multiple methods to identify these variants: read-pair analysis, split-read analysis, and de novo assembly, integrating the information from these three methodologies to validate variants and reduce false positives. For individual samples that had insertions and deletions, sample BCF format files were merged and filtered based on sample missingness (< 50%), followed by the removal of heterozygous calls. As described above, the population differentiation statistic F_ST_ was calculated using vcftools, to identify SVs unique to populations. To confirm any SVs occurring in genes associated with insecticide resistance, the bcftools software was used for visual inspection.

## Electronic supplementary material

Below is the link to the electronic supplementary material.

Supplementary Material 1

## Data Availability

All raw fastq files generated in this work for Ethiopian An. stephensi is publicly available (see PRJEB66077 for accession numbers, https://www.ncbi.nlm.nih.gov/bioproject/PRJEB66077/). Accession numbers for publicly available raw used in this study can be found in Supplementary Table S7. Analysis scripts are available at https://github.com/LSHTMPathogenSeqLab.
