# Supplementary material for: Genome wide population genetics and molecular surveillance of insecticide resistance in Anopheles stephensi mosquitoes from Awash Sebat Kilo in Ethiopia
Source: Sci Rep. 2025 May 12;15:16443. doi: 10.1038/s41598-025-95814-0 (PMC12069653; doi:10.1038/s41598-025-95814-0)
Supplement: Supplementary file 1 — Supplementary Material 1 [file 41598_2025_95814_MOESM1_ESM.docx]

**Supplementary Table S1. Average Nucleotide Diversity per Population**

| Population | π |
| --- | --- |
| Ethiopia | 0.189 |
| India Field | 0.181 |
| India Colony | 0.163 |
| Pakistan Colony | 0.176 |

**Supplementary Table S2.** Exonic Genomic Positions with an Fst of 1

| Chrom | Pos | Ref | Alt | Population |
| --- | --- | --- | --- | --- |
| NC_050201.1 | 3119508 | A | G | Ethiopia_PakistanCol |
| NC_050201.1 | 3718010 | C | A | IndiaCol_PakistanCol |
| NC_050201.1 | 3718012 | G | T | IndiaCol_PakistanCol |
| NC_050201.1 | 3791130 | G | T | IndiaWT_PakistanCol |
| NC_050201.1 | 3791317 | C | T | Ethiopia_PakistanCol |
| NC_050201.1 | 3791334 | C | T | Ethiopia_PakistanCol |
| NC_050201.1 | 3791334 | C | T | IndiaWT_PakistanCol |
| NC_050201.1 | 3902652 | A | T | Ethiopia_PakistanCol |
| NC_050201.1 | 3941791 | T | C | IndiaWT_PakistanCol |
| NC_050201.1 | 3942785 | T | C | Ethiopia_PakistanCol |
| NC_050201.1 | 4307500 | A | T | Ethiopia_PakistanCol |
| NC_050201.1 | 4311965 | A | G | Ethiopia_PakistanCol |
| NC_050201.1 | 4311965 | A | G | IndiaWT_PakistanCol |
| NC_050201.1 | 4311968 | C | A | Ethiopia_PakistanCol |
| NC_050201.1 | 4311968 | C | A | IndiaWT_PakistanCol |
| NC_050201.1 | 4988708 | C | T | Ethiopia_IndCol |
| NC_050201.1 | 5065527 | G | A | IndiaWT_PakistanCol |
| NC_050201.1 | 5734519 | T | G | Ethiopia_IndCol |
| NC_050201.1 | 5742090 | G | A | Ethiopia_IndCol |
| NC_050201.1 | 5742090 | G | A | IndiaCol_PakistanCol |
| NC_050201.1 | 6097418 | A | G | Ethiopia_IndCol |
| NC_050201.1 | 7039544 | A | G | Ethiopia_PakistanCol |
| NC_050201.1 | 7615454 | T | C | Ethiopia_PakistanCol |
| NC_050201.1 | 8078596 | T | C | Ethiopia_PakistanCol |
| NC_050201.1 | 8079343 | T | C | Ethiopia_PakistanCol |
| NC_050201.1 | 8162184 | G | A | Ethiopia_PakistanCol |
| NC_050201.1 | 8163033 | G | T | Ethiopia_PakistanCol |
| NC_050201.1 | 8163075 | C | T | Ethiopia_PakistanCol |
| NC_050201.1 | 8180979 | C | T | IndiaWT_PakistanCol |
| NC_050201.1 | 8194458 | G | A | IndiaWT_PakistanCol |
| NC_050201.1 | 8206010 | A | T | Ethiopia_PakistanCol |
| NC_050201.1 | 8375533 | C | T | Ethiopia_PakistanCol |
| NC_050201.1 | 8384181 | G | A | IndiaCol_PakistanCol |
| NC_050201.1 | 8614482 | A | T | IndiaWT_PakistanCol |
| NC_050201.1 | 8614523 | T | A | IndiaWT_PakistanCol |
| NC_050201.1 | 8615804 | A | G | Ethiopia_PakistanCol |
| NC_050201.1 | 9051868 | T | A | Ethiopia_PakistanCol |
| NC_050201.1 | 9051883 | C | T | Ethiopia_PakistanCol |
| NC_050201.1 | 9122655 | T | G | IndiaWT_PakistanCol |
| NC_050201.1 | 9122656 | A | G | IndiaWT_PakistanCol |
| NC_050201.1 | 9140498 | T | C | IndiaWT_PakistanCol |
| NC_050201.1 | 9140506 | C | T | IndiaWT_PakistanCol |
| NC_050201.1 | 9140997 | A | T | IndiaWT_PakistanCol |
| NC_050201.1 | 9141684 | A | T | IndiaWT_PakistanCol |
| NC_050201.1 | 9141695 | A | T | IndiaWT_PakistanCol |
| NC_050201.1 | 9141761 | C | T | IndiaWT_PakistanCol |
| NC_050201.1 | 9141763 | C | T | IndiaWT_PakistanCol |
| NC_050201.1 | 9141773 | C | A | IndiaWT_PakistanCol |
| NC_050201.1 | 9141820 | G | A | IndiaWT_PakistanCol |
| NC_050201.1 | 9141926 | G | A | IndiaWT_PakistanCol |
| NC_050201.1 | 9157464 | A | C | IndiaWT_PakistanCol |
| NC_050201.1 | 9190316 | T | A | IndiaWT_PakistanCol |
| NC_050201.1 | 9239863 | A | T | IndiaWT_PakistanCol |
| NC_050201.1 | 9243437 | C | T | IndiaWT_PakistanCol |
| NC_050201.1 | 9551835 | T | C | Ethiopia_PakistanCol |
| NC_050201.1 | 9553768 | G | C | Ethiopia_IndCol |
| NC_050201.1 | 9609233 | T | C | IndiaWT_PakistanCol |
| NC_050201.1 | 9609237 | C | T | IndiaWT_PakistanCol |
| NC_050201.1 | 9609527 | C | T | IndiaWT_PakistanCol |
| NC_050201.1 | 9768201 | C | A | Ethiopia_PakistanCol |
| NC_050201.1 | 9838585 | T | G | Ethiopia_PakistanCol |
| NC_050201.1 | 9838805 | C | G | Ethiopia_PakistanCol |
| NC_050201.1 | 9840440 | C | A | Ethiopia_PakistanCol |
| NC_050201.1 | 9897192 | A | C | IndiaCol_PakistanCol |
| NC_050201.1 | 10760763 | G | T | IndiaCol_PakistanCol |
| NC_050201.1 | 10778472 | A | T | IndiaWT_PakistanCol |
| NC_050201.1 | 10778582 | T | G | IndiaWT_PakistanCol |
| NC_050201.1 | 10782800 | T | A | Ethiopia_PakistanCol |
| NC_050201.1 | 11520626 | T | A | Ethiopia_PakistanCol |
| NC_050201.1 | 11520661 | C | T | Ethiopia_PakistanCol |
| NC_050201.1 | 11520687 | A | C | Ethiopia_PakistanCol |
| NC_050201.1 | 11520689 | T | C | Ethiopia_PakistanCol |
| NC_050201.1 | 11520714 | G | A | Ethiopia_PakistanCol |
| NC_050201.1 | 12110970 | G | T | IndiaCol_PakistanCol |
| NC_050201.1 | 12110970 | G | T | IndiaWT_PakistanCol |
| NC_050201.1 | 12110985 | A | G | IndiaCol_PakistanCol |
| NC_050201.1 | 12110985 | A | G | IndiaWT_PakistanCol |
| NC_050201.1 | 12111001 | A | C | IndiaWT_PakistanCol |
| NC_050201.1 | 12111003 | C | T | IndiaWT_PakistanCol |
| NC_050201.1 | 12111017 | C | G | IndiaWT_PakistanCol |
| NC_050201.1 | 12111020 | A | G | IndiaWT_PakistanCol |
| NC_050201.1 | 12612829 | C | T | IndiaCol_PakistanCol |
| NC_050201.1 | 12612829 | C | T | IndiaWT_PakistanCol |
| NC_050201.1 | 13221246 | A | T | IndiaWT_PakistanCol |
| NC_050201.1 | 13892546 | G | C | IndiaWT_PakistanCol |
| NC_050201.1 | 13892564 | A | G | IndiaWT_PakistanCol |
| NC_050201.1 | 13894630 | A | G | IndiaWT_PakistanCol |
| NC_050201.1 | 13894646 | T | A | IndiaWT_PakistanCol |
| NC_050201.1 | 13895385 | T | C | IndiaWT_PakistanCol |
| NC_050201.1 | 14482898 | C | A | Ethiopia_PakistanCol |
| NC_050201.1 | 14640254 | A | G | Ethiopia_IndCol |
| NC_050201.1 | 15463678 | T | C | Ethiopia_PakistanCol |
| NC_050201.1 | 15744217 | T | C | Ethiopia_PakistanCol |
| NC_050201.1 | 15744217 | T | C | IndiaCol_PakistanCol |
| NC_050201.1 | 15744217 | T | C | IndiaWT_PakistanCol |
| NC_050201.1 | 16515625 | C | G | Ethiopia_IndCol |
| NC_050201.1 | 16515625 | C | G | Ethiopia_PakistanCol |
| NC_050201.1 | 16545310 | A | G | IndiaWT_PakistanCol |
| NC_050201.1 | 17001554 | C | A | IndiaCol_PakistanCol |
| NC_050201.1 | 18274312 | T | C | IndiaCol_PakistanCol |
| NC_050201.1 | 19698896 | C | T | Ethiopia_PakistanCol |
| NC_050201.1 | 19698896 | C | T | IndiaCol_PakistanCol |
| NC_050201.1 | 19698896 | C | T | IndiaWT_PakistanCol |
| NC_050201.1 | 20989399 | G | A | IndiaCol_PakistanCol |
| NC_050201.1 | 20989399 | G | A | IndiaWT_PakistanCol |
| NC_050201.1 | 21333806 | C | A | Ethiopia_PakistanCol |
| NC_050201.1 | 21333806 | C | A | IndiaCol_PakistanCol |
| NC_050201.1 | 21333806 | C | A | IndiaWT_PakistanCol |
| NC_050201.1 | 21345179 | G | A | Ethiopia_PakistanCol |
| NC_050201.1 | 21345179 | G | A | IndiaCol_PakistanCol |
| NC_050201.1 | 21345179 | G | A | IndiaWT_PakistanCol |
| NC_050201.1 | 21413422 | C | T | Ethiopia_PakistanCol |
| NC_050201.1 | 21413422 | C | T | IndiaCol_PakistanCol |
| NC_050201.1 | 21413422 | C | T | IndiaWT_PakistanCol |
| NC_050202.1 | 3109410 | A | T | IndiaCol_PakistanCol |
| NC_050202.1 | 7482256 | C | T | IndiaCol_PakistanCol |
| NC_050202.1 | 9952151 | A | T | Ethiopia_PakistanCol |
| NC_050202.1 | 9952151 | A | T | IndiaWT_PakistanCol |
| NC_050202.1 | 11373952 | A | T | IndiaCol_PakistanCol |
| NC_050202.1 | 12194118 | G | A | Ethiopia_IndCol |
| NC_050202.1 | 21855742 | A | C | Ethiopia_IndCol |
| NC_050202.1 | 21855742 | A | C | Ethiopia_PakistanCol |
| NC_050202.1 | 22897925 | C | T | Ethiopia_PakistanCol |
| NC_050202.1 | 25122013 | T | C | IndiaCol_PakistanCol |
| NC_050202.1 | 25122019 | T | C | IndiaCol_PakistanCol |
| NC_050202.1 | 26119573 | A | G | IndiaCol_IndiaWT |
| NC_050202.1 | 26119573 | A | G | IndiaWT_PakistanCol |
| NC_050202.1 | 28051509 | C | T | Ethiopia_IndWT |
| NC_050202.1 | 28051509 | C | T | IndiaWT_PakistanCol |
| NC_050202.1 | 28051531 | G | T | Ethiopia_IndWT |
| NC_050202.1 | 28051531 | G | T | IndiaWT_PakistanCol |
| NC_050202.1 | 28098876 | G | A | Ethiopia_PakistanCol |
| NC_050202.1 | 28664257 | C | T | IndiaCol_PakistanCol |
| NC_050202.1 | 28669808 | G | A | IndiaCol_PakistanCol |
| NC_050202.1 | 28818901 | G | A | IndiaCol_PakistanCol |
| NC_050202.1 | 28824869 | G | A | IndiaWT_PakistanCol |
| NC_050202.1 | 31076030 | T | C | IndiaCol_PakistanCol |
| NC_050202.1 | 31080402 | G | C | Ethiopia_PakistanCol |
| NC_050202.1 | 31080402 | G | C | IndiaCol_PakistanCol |
| NC_050202.1 | 31080402 | G | C | IndiaWT_PakistanCol |
| NC_050202.1 | 31080439 | C | T | IndiaWT_PakistanCol |
| NC_050202.1 | 31081200 | G | A | IndiaCol_PakistanCol |
| NC_050202.1 | 31081648 | A | G | IndiaWT_PakistanCol |
| NC_050202.1 | 31081680 | A | C | IndiaCol_PakistanCol |
| NC_050202.1 | 31081680 | A | C | IndiaWT_PakistanCol |
| NC_050202.1 | 31081692 | G | A | IndiaWT_PakistanCol |
| NC_050202.1 | 31083166 | G | A | Ethiopia_PakistanCol |
| NC_050202.1 | 31083166 | G | A | IndiaCol_PakistanCol |
| NC_050202.1 | 31827761 | A | G | Ethiopia_PakistanCol |
| NC_050202.1 | 31827761 | A | G | IndiaWT_PakistanCol |
| NC_050202.1 | 31879735 | T | A | IndiaCol_PakistanCol |
| NC_050202.1 | 31882580 | G | T | IndiaCol_PakistanCol |
| NC_050202.1 | 31894673 | G | T | IndiaCol_PakistanCol |
| NC_050202.1 | 31897906 | G | A | Ethiopia_PakistanCol |
| NC_050202.1 | 32153812 | G | T | IndiaWT_PakistanCol |
| NC_050202.1 | 32456321 | A | T | IndiaCol_PakistanCol |
| NC_050202.1 | 33450308 | G | A | Ethiopia_IndWT |
| NC_050202.1 | 33450308 | G | A | IndiaWT_PakistanCol |
| NC_050202.1 | 33450309 | G | C | Ethiopia_IndWT |
| NC_050202.1 | 33450309 | G | C | IndiaWT_PakistanCol |
| NC_050202.1 | 37755975 | G | T | IndiaCol_PakistanCol |
| NC_050202.1 | 37755975 | G | T | IndiaWT_PakistanCol |
| NC_050202.1 | 46618307 | A | C | Ethiopia_PakistanCol |
| NC_050202.1 | 46618307 | A | C | IndiaWT_PakistanCol |
| NC_050202.1 | 47046531 | T | A | Ethiopia_IndCol |
| NC_050202.1 | 47046531 | T | A | Ethiopia_PakistanCol |
| NC_050202.1 | 48149481 | G | C | Ethiopia_PakistanCol |
| NC_050202.1 | 48241117 | G | T | IndiaCol_PakistanCol |
| NC_050202.1 | 48410606 | C | A | IndiaCol_PakistanCol |
| NC_050202.1 | 48410943 | G | A | IndiaCol_PakistanCol |
| NC_050202.1 | 48410943 | G | A | IndiaWT_PakistanCol |
| NC_050202.1 | 48411001 | G | C | IndiaCol_PakistanCol |
| NC_050202.1 | 48412390 | A | G | Ethiopia_PakistanCol |
| NC_050202.1 | 48412390 | A | G | IndiaWT_PakistanCol |
| NC_050202.1 | 48412647 | G | A | Ethiopia_PakistanCol |
| NC_050202.1 | 48438259 | A | T | Ethiopia_PakistanCol |
| NC_050202.1 | 48438259 | A | T | IndiaCol_PakistanCol |
| NC_050202.1 | 48438259 | A | T | IndiaWT_PakistanCol |
| NC_050202.1 | 48438260 | A | C | Ethiopia_PakistanCol |
| NC_050202.1 | 48438260 | A | C | IndiaCol_PakistanCol |
| NC_050202.1 | 48438260 | A | C | IndiaWT_PakistanCol |
| NC_050202.1 | 48469238 | C | A | Ethiopia_PakistanCol |
| NC_050202.1 | 48469238 | C | A | IndiaCol_PakistanCol |
| NC_050202.1 | 48469238 | C | A | IndiaWT_PakistanCol |
| NC_050202.1 | 48469289 | G | A | Ethiopia_PakistanCol |
| NC_050202.1 | 48469289 | G | A | IndiaCol_PakistanCol |
| NC_050202.1 | 48469301 | G | T | Ethiopia_PakistanCol |
| NC_050202.1 | 48469301 | G | T | IndiaCol_PakistanCol |
| NC_050202.1 | 48469334 | T | A | Ethiopia_PakistanCol |
| NC_050202.1 | 48469334 | T | A | IndiaCol_PakistanCol |
| NC_050202.1 | 48469349 | T | A | IndiaCol_PakistanCol |
| NC_050202.1 | 48469372 | A | G | Ethiopia_PakistanCol |
| NC_050202.1 | 48469372 | A | G | IndiaCol_PakistanCol |
| NC_050202.1 | 48470798 | A | T | Ethiopia_PakistanCol |
| NC_050202.1 | 48470798 | A | T | IndiaCol_PakistanCol |
| NC_050202.1 | 48482107 | A | T | IndiaCol_PakistanCol |
| NC_050202.1 | 48489565 | T | A | Ethiopia_PakistanCol |
| NC_050202.1 | 48489565 | T | A | IndiaCol_PakistanCol |
| NC_050202.1 | 48489565 | T | A | IndiaWT_PakistanCol |
| NC_050202.1 | 48490016 | T | C | IndiaCol_PakistanCol |
| NC_050202.1 | 48490016 | T | C | IndiaWT_PakistanCol |
| NC_050202.1 | 48490030 | C | T | IndiaCol_PakistanCol |
| NC_050202.1 | 48490030 | C | T | IndiaWT_PakistanCol |
| NC_050202.1 | 48674035 | A | G | IndiaCol_PakistanCol |
| NC_050202.1 | 49095962 | A | T | IndiaCol_PakistanCol |
| NC_050202.1 | 50561415 | A | G | IndiaCol_PakistanCol |
| NC_050202.1 | 51959061 | A | C | Ethiopia_PakistanCol |
| NC_050202.1 | 52214152 | A | T | Ethiopia_PakistanCol |
| NC_050202.1 | 52214156 | G | A | Ethiopia_PakistanCol |
| NC_050202.1 | 52214158 | C | T | Ethiopia_PakistanCol |
| NC_050202.1 | 52214163 | A | G | Ethiopia_PakistanCol |
| NC_050202.1 | 52569315 | T | G | Ethiopia_PakistanCol |
| NC_050202.1 | 52569315 | T | G | IndiaWT_PakistanCol |
| NC_050202.1 | 52569318 | T | A | Ethiopia_PakistanCol |
| NC_050202.1 | 52569318 | T | A | IndiaWT_PakistanCol |
| NC_050202.1 | 53430051 | A | G | Ethiopia_PakistanCol |
| NC_050202.1 | 53430392 | C | A | Ethiopia_PakistanCol |
| NC_050202.1 | 53430397 | T | C | Ethiopia_PakistanCol |
| NC_050202.1 | 53881798 | G | A | Ethiopia_PakistanCol |
| NC_050202.1 | 53881798 | G | A | IndiaCol_PakistanCol |
| NC_050202.1 | 53881798 | G | A | IndiaWT_PakistanCol |
| NC_050202.1 | 53883345 | A | G | IndiaCol_IndiaWT |
| NC_050202.1 | 57028554 | G | A | IndiaWT_PakistanCol |
| NC_050202.1 | 57870459 | T | G | IndiaCol_IndiaWT |
| NC_050202.1 | 57870459 | T | G | IndiaCol_PakistanCol |
| NC_050202.1 | 59438512 | C | A | Ethiopia_PakistanCol |
| NC_050202.1 | 59438515 | G | A | Ethiopia_PakistanCol |
| NC_050202.1 | 60511219 | C | T | IndiaWT_PakistanCol |
| NC_050202.1 | 60511262 | G | A | Ethiopia_PakistanCol |
| NC_050202.1 | 60511262 | G | A | IndiaCol_PakistanCol |
| NC_050202.1 | 60511262 | G | A | IndiaWT_PakistanCol |
| NC_050202.1 | 60513095 | G | T | Ethiopia_PakistanCol |
| NC_050202.1 | 60513095 | G | T | IndiaWT_PakistanCol |
| NC_050202.1 | 60513112 | C | G | Ethiopia_PakistanCol |
| NC_050202.1 | 60513112 | C | G | IndiaWT_PakistanCol |
| NC_050202.1 | 60513114 | G | C | Ethiopia_PakistanCol |
| NC_050202.1 | 60513114 | G | C | IndiaWT_PakistanCol |
| NC_050202.1 | 60513267 | T | C | IndiaCol_PakistanCol |
| NC_050202.1 | 60513269 | T | A | IndiaCol_PakistanCol |
| NC_050202.1 | 60517135 | C | A | Ethiopia_PakistanCol |
| NC_050202.1 | 60518174 | G | C | Ethiopia_PakistanCol |
| NC_050202.1 | 60518174 | G | C | IndiaCol_PakistanCol |
| NC_050202.1 | 60518174 | G | C | IndiaWT_PakistanCol |
| NC_050202.1 | 60518340 | T | G | Ethiopia_PakistanCol |
| NC_050202.1 | 60518349 | A | C | Ethiopia_PakistanCol |
| NC_050202.1 | 60518349 | A | C | IndiaCol_PakistanCol |
| NC_050202.1 | 60518349 | A | C | IndiaWT_PakistanCol |
| NC_050202.1 | 60518355 | A | T | Ethiopia_PakistanCol |
| NC_050202.1 | 60518355 | A | T | IndiaCol_PakistanCol |
| NC_050202.1 | 60518495 | A | C | Ethiopia_PakistanCol |
| NC_050202.1 | 60797317 | T | C | Ethiopia_PakistanCol |
| NC_050202.1 | 61297967 | C | T | Ethiopia_PakistanCol |
| NC_050202.1 | 62310580 | A | G | Ethiopia_PakistanCol |
| NC_050202.1 | 62311588 | C | A | Ethiopia_PakistanCol |
| NC_050202.1 | 62521853 | G | A | Ethiopia_PakistanCol |
| NC_050202.1 | 62589377 | G | A | Ethiopia_PakistanCol |
| NC_050202.1 | 63662538 | A | T | Ethiopia_PakistanCol |
| NC_050202.1 | 63662619 | C | T | Ethiopia_PakistanCol |
| NC_050202.1 | 69454519 | A | G | Ethiopia_PakistanCol |
| NC_050202.1 | 69454526 | G | T | Ethiopia_PakistanCol |
| NC_050202.1 | 70224844 | G | T | Ethiopia_PakistanCol |
| NC_050202.1 | 74673525 | G | C | Ethiopia_PakistanCol |
| NC_050202.1 | 74673525 | G | C | IndiaCol_PakistanCol |
| NC_050202.1 | 74673525 | G | C | IndiaWT_PakistanCol |
| NC_050202.1 | 74673550 | G | C | IndiaCol_PakistanCol |
| NC_050202.1 | 74673550 | G | C | IndiaWT_PakistanCol |
| NC_050202.1 | 74673570 | G | A | Ethiopia_PakistanCol |
| NC_050202.1 | 74673570 | G | A | IndiaCol_PakistanCol |
| NC_050202.1 | 74673570 | G | A | IndiaWT_PakistanCol |
| NC_050202.1 | 74673574 | A | T | Ethiopia_PakistanCol |
| NC_050202.1 | 74673574 | A | T | IndiaCol_PakistanCol |
| NC_050202.1 | 74673574 | A | T | IndiaWT_PakistanCol |
| NC_050202.1 | 74673578 | C | T | Ethiopia_PakistanCol |
| NC_050202.1 | 74673578 | C | T | IndiaCol_PakistanCol |
| NC_050202.1 | 74673578 | C | T | IndiaWT_PakistanCol |
| NC_050202.1 | 74673585 | A | G | Ethiopia_PakistanCol |
| NC_050202.1 | 74673585 | A | G | IndiaCol_PakistanCol |
| NC_050202.1 | 74673585 | A | G | IndiaWT_PakistanCol |
| NC_050202.1 | 74673752 | G | A | IndiaCol_PakistanCol |
| NC_050202.1 | 74673779 | A | T | IndiaCol_PakistanCol |
| NC_050202.1 | 74673779 | A | T | IndiaWT_PakistanCol |
| NC_050202.1 | 74673785 | T | C | IndiaCol_PakistanCol |
| NC_050202.1 | 74673785 | T | C | IndiaWT_PakistanCol |
| NC_050202.1 | 74673809 | C | T | IndiaCol_PakistanCol |
| NC_050202.1 | 76662754 | T | A | IndiaCol_PakistanCol |
| NC_050202.1 | 76681957 | T | C | IndiaCol_PakistanCol |
| NC_050202.1 | 76690626 | C | T | Ethiopia_IndWT |
| NC_050202.1 | 76690626 | C | T | IndiaWT_PakistanCol |
| NC_050202.1 | 76691902 | T | C | IndiaCol_PakistanCol |
| NC_050202.1 | 76691924 | G | C | IndiaCol_PakistanCol |
| NC_050202.1 | 76696583 | C | T | IndiaCol_PakistanCol |
| NC_050202.1 | 76696583 | C | T | IndiaWT_PakistanCol |
| NC_050202.1 | 76705411 | C | T | IndiaCol_PakistanCol |
| NC_050202.1 | 76705411 | C | T | IndiaWT_PakistanCol |
| NC_050202.1 | 76708309 | T | A | IndiaCol_PakistanCol |
| NC_050202.1 | 76711139 | A | C | IndiaCol_PakistanCol |
| NC_050202.1 | 76844191 | C | T | IndiaCol_PakistanCol |
| NC_050202.1 | 76900336 | C | T | Ethiopia_PakistanCol |
| NC_050202.1 | 76900336 | C | T | IndiaWT_PakistanCol |
| NC_050202.1 | 76900339 | G | A | Ethiopia_PakistanCol |
| NC_050202.1 | 76900339 | G | A | IndiaCol_PakistanCol |
| NC_050202.1 | 76900339 | G | A | IndiaWT_PakistanCol |
| NC_050202.1 | 76900434 | G | A | Ethiopia_PakistanCol |
| NC_050202.1 | 76900434 | G | A | IndiaWT_PakistanCol |
| NC_050202.1 | 76900500 | G | A | Ethiopia_PakistanCol |
| NC_050202.1 | 76900500 | G | A | IndiaWT_PakistanCol |
| NC_050202.1 | 79918267 | T | C | Ethiopia_PakistanCol |
| NC_050202.1 | 79961413 | A | G | IndiaWT_PakistanCol |
| NC_050202.1 | 81635957 | T | C | Ethiopia_PakistanCol |
| NC_050202.1 | 81747896 | T | C | IndiaCol_PakistanCol |
| NC_050202.1 | 81747908 | T | C | IndiaCol_PakistanCol |
| NC_050202.1 | 82407720 | A | G | Ethiopia_PakistanCol |
| NC_050202.1 | 82408207 | G | C | Ethiopia_PakistanCol |
| NC_050202.1 | 82409362 | G | A | Ethiopia_PakistanCol |
| NC_050202.1 | 82779862 | G | A | IndiaCol_PakistanCol |
| NC_050202.1 | 82874440 | A | T | IndiaCol_PakistanCol |
| NC_050202.1 | 82874647 | T | C | Ethiopia_IndCol |
| NC_050202.1 | 82874647 | T | C | IndiaCol_PakistanCol |
| NC_050202.1 | 82879048 | G | T | Ethiopia_PakistanCol |
| NC_050202.1 | 82879048 | G | T | IndiaCol_PakistanCol |
| NC_050202.1 | 82879048 | G | T | IndiaWT_PakistanCol |
| NC_050202.1 | 82909566 | T | C | IndiaCol_PakistanCol |
| NC_050202.1 | 83096170 | A | G | IndiaWT_PakistanCol |
| NC_050202.1 | 83737074 | A | C | Ethiopia_PakistanCol |
| NC_050202.1 | 83737074 | A | C | IndiaWT_PakistanCol |
| NC_050202.1 | 83737080 | T | C | Ethiopia_PakistanCol |
| NC_050202.1 | 83737080 | T | C | IndiaWT_PakistanCol |
| NC_050202.1 | 83737097 | A | G | Ethiopia_PakistanCol |
| NC_050202.1 | 83737097 | A | G | IndiaWT_PakistanCol |
| NC_050202.1 | 83737128 | C | T | Ethiopia_PakistanCol |
| NC_050202.1 | 83737128 | C | T | IndiaWT_PakistanCol |
| NC_050202.1 | 83737185 | T | C | Ethiopia_PakistanCol |
| NC_050202.1 | 84269167 | G | T | IndiaWT_PakistanCol |
| NC_050202.1 | 84782288 | T | G | Ethiopia_IndCol |
| NC_050202.1 | 85597280 | G | A | Ethiopia_IndCol |
| NC_050202.1 | 85597292 | A | C | Ethiopia_IndCol |
| NC_050202.1 | 85715908 | C | G | Ethiopia_IndCol |
| NC_050202.1 | 85715908 | C | A | Ethiopia_IndCol |
| NC_050202.1 | 86513153 | A | G | Ethiopia_PakistanCol |
| NC_050202.1 | 86601291 | T | C | Ethiopia_PakistanCol |
| NC_050202.1 | 86987640 | G | A | IndiaWT_PakistanCol |
| NC_050202.1 | 86987679 | T | C | IndiaWT_PakistanCol |
| NC_050202.1 | 87521490 | G | T | Ethiopia_PakistanCol |
| NC_050202.1 | 87521491 | G | T | Ethiopia_PakistanCol |
| NC_050202.1 | 87523051 | C | T | Ethiopia_PakistanCol |
| NC_050202.1 | 88016796 | C | T | IndiaCol_PakistanCol |
| NC_050202.1 | 88016796 | C | T | IndiaWT_PakistanCol |
| NC_050202.1 | 88115085 | G | A | IndiaWT_PakistanCol |
| NC_050202.1 | 88115101 | T | A | IndiaWT_PakistanCol |
| NC_050202.1 | 88115103 | G | C | IndiaCol_PakistanCol |
| NC_050202.1 | 88115103 | G | C | IndiaWT_PakistanCol |
| NC_050202.1 | 88128120 | A | G | Ethiopia_PakistanCol |
| NC_050202.1 | 88400408 | G | A | IndiaCol_PakistanCol |
| NC_050202.1 | 88400408 | G | A | IndiaWT_PakistanCol |
| NC_050202.1 | 88407741 | T | C | IndiaCol_PakistanCol |
| NC_050202.1 | 88407880 | C | T | Ethiopia_PakistanCol |
| NC_050202.1 | 88407880 | C | T | IndiaCol_PakistanCol |
| NC_050202.1 | 88407880 | C | T | IndiaWT_PakistanCol |
| NC_050202.1 | 88411168 | G | A | IndiaCol_PakistanCol |
| NC_050202.1 | 88425807 | T | G | Ethiopia_PakistanCol |
| NC_050202.1 | 88425807 | T | G | IndiaWT_PakistanCol |
| NC_050202.1 | 89379413 | G | A | Ethiopia_PakistanCol |
| NC_050202.1 | 89379686 | A | G | Ethiopia_PakistanCol |
| NC_050202.1 | 92529285 | G | A | Ethiopia_PakistanCol |
| NC_050202.1 | 92529285 | G | A | IndiaCol_PakistanCol |
| NC_050203.1 | 604355 | T | G | IndiaCol_PakistanCol |
| NC_050203.1 | 604373 | C | T | IndiaCol_PakistanCol |
| NC_050203.1 | 706261 | G | A | IndiaCol_PakistanCol |
| NC_050203.1 | 708084 | T | C | IndiaCol_PakistanCol |
| NC_050203.1 | 742749 | A | T | IndiaCol_IndiaWT |
| NC_050203.1 | 1478395 | T | C | IndiaWT_PakistanCol |
| NC_050203.1 | 2953328 | A | T | IndiaCol_PakistanCol |
| NC_050203.1 | 2953368 | T | G | IndiaCol_PakistanCol |
| NC_050203.1 | 3044395 | T | C | IndiaCol_PakistanCol |
| NC_050203.1 | 3515754 | G | A | IndiaCol_PakistanCol |
| NC_050203.1 | 3515769 | G | A | IndiaCol_PakistanCol |
| NC_050203.1 | 3701348 | C | T | IndiaWT_PakistanCol |
| NC_050203.1 | 6931642 | A | G | IndiaWT_PakistanCol |
| NC_050203.1 | 11162190 | T | A | Ethiopia_PakistanCol |
| NC_050203.1 | 11246922 | C | T | IndiaCol_PakistanCol |
| NC_050203.1 | 12203048 | A | G | Ethiopia_PakistanCol |
| NC_050203.1 | 12203054 | A | C | Ethiopia_PakistanCol |
| NC_050203.1 | 12220079 | T | C | Ethiopia_PakistanCol |
| NC_050203.1 | 13119125 | A | C | IndiaWT_PakistanCol |
| NC_050203.1 | 13864464 | A | G | Ethiopia_PakistanCol |
| NC_050203.1 | 15864890 | T | A | Ethiopia_PakistanCol |
| NC_050203.1 | 16718911 | G | C | IndiaWT_PakistanCol |
| NC_050203.1 | 17891968 | G | C | IndiaCol_PakistanCol |
| NC_050203.1 | 18127179 | C | A | Ethiopia_PakistanCol |
| NC_050203.1 | 18181308 | G | T | IndiaCol_PakistanCol |
| NC_050203.1 | 18540033 | T | A | Ethiopia_PakistanCol |
| NC_050203.1 | 18540301 | T | C | Ethiopia_PakistanCol |
| NC_050203.1 | 18540301 | T | C | IndiaCol_PakistanCol |
| NC_050203.1 | 18547474 | A | G | IndiaWT_PakistanCol |
| NC_050203.1 | 18547477 | C | A | IndiaWT_PakistanCol |
| NC_050203.1 | 18550892 | T | C | Ethiopia_PakistanCol |
| NC_050203.1 | 18550892 | T | C | IndiaWT_PakistanCol |
| NC_050203.1 | 18552739 | A | G | Ethiopia_PakistanCol |
| NC_050203.1 | 18552791 | A | T | Ethiopia_PakistanCol |
| NC_050203.1 | 18552843 | A | G | Ethiopia_PakistanCol |
| NC_050203.1 | 18552917 | C | T | Ethiopia_PakistanCol |
| NC_050203.1 | 18552936 | A | G | Ethiopia_PakistanCol |
| NC_050203.1 | 18562034 | C | T | Ethiopia_PakistanCol |
| NC_050203.1 | 18562053 | G | A | Ethiopia_PakistanCol |
| NC_050203.1 | 18563626 | G | A | Ethiopia_PakistanCol |
| NC_050203.1 | 18563656 | C | T | Ethiopia_PakistanCol |
| NC_050203.1 | 18578026 | C | A | Ethiopia_PakistanCol |
| NC_050203.1 | 18578094 | A | G | Ethiopia_PakistanCol |
| NC_050203.1 | 18578165 | T | G | Ethiopia_PakistanCol |
| NC_050203.1 | 18578396 | G | A | Ethiopia_PakistanCol |
| NC_050203.1 | 18578401 | G | A | Ethiopia_PakistanCol |
| NC_050203.1 | 18578401 | G | T | Ethiopia_PakistanCol |
| NC_050203.1 | 18581682 | C | T | Ethiopia_PakistanCol |
| NC_050203.1 | 18581708 | C | A | Ethiopia_PakistanCol |
| NC_050203.1 | 18582285 | A | T | Ethiopia_PakistanCol |
| NC_050203.1 | 18786650 | A | G | Ethiopia_PakistanCol |
| NC_050203.1 | 18786650 | A | C | Ethiopia_PakistanCol |
| NC_050203.1 | 18786650 | A | G | IndiaCol_PakistanCol |
| NC_050203.1 | 18786650 | A | C | IndiaCol_PakistanCol |
| NC_050203.1 | 18786746 | G | A | Ethiopia_PakistanCol |
| NC_050203.1 | 18786746 | G | A | IndiaWT_PakistanCol |
| NC_050203.1 | 18786856 | A | G | Ethiopia_PakistanCol |
| NC_050203.1 | 18786950 | G | A | Ethiopia_PakistanCol |
| NC_050203.1 | 18786950 | G | A | IndiaWT_PakistanCol |
| NC_050203.1 | 18786994 | G | A | IndiaWT_PakistanCol |
| NC_050203.1 | 18787153 | T | C | IndiaWT_PakistanCol |
| NC_050203.1 | 18788394 | T | A | Ethiopia_PakistanCol |
| NC_050203.1 | 18788395 | G | T | Ethiopia_PakistanCol |
| NC_050203.1 | 18788397 | C | T | Ethiopia_PakistanCol |
| NC_050203.1 | 18788466 | G | A | Ethiopia_PakistanCol |
| NC_050203.1 | 18788518 | T | C | Ethiopia_PakistanCol |
| NC_050203.1 | 18790676 | G | C | Ethiopia_PakistanCol |
| NC_050203.1 | 18790727 | T | G | Ethiopia_PakistanCol |
| NC_050203.1 | 18790973 | A | G | Ethiopia_PakistanCol |
| NC_050203.1 | 18791084 | A | G | Ethiopia_PakistanCol |
| NC_050203.1 | 18791279 | C | T | Ethiopia_PakistanCol |
| NC_050203.1 | 18791291 | A | C | Ethiopia_PakistanCol |
| NC_050203.1 | 18793024 | C | T | Ethiopia_PakistanCol |
| NC_050203.1 | 18793064 | C | G | Ethiopia_PakistanCol |
| NC_050203.1 | 18793160 | G | T | Ethiopia_PakistanCol |
| NC_050203.1 | 18793657 | G | A | Ethiopia_PakistanCol |
| NC_050203.1 | 18793698 | T | G | Ethiopia_PakistanCol |
| NC_050203.1 | 18794800 | T | C | Ethiopia_PakistanCol |
| NC_050203.1 | 18795044 | C | T | Ethiopia_PakistanCol |
| NC_050203.1 | 18795053 | G | A | Ethiopia_PakistanCol |
| NC_050203.1 | 18795907 | C | T | Ethiopia_PakistanCol |
| NC_050203.1 | 18795918 | T | C | Ethiopia_PakistanCol |
| NC_050203.1 | 18795922 | G | A | Ethiopia_PakistanCol |
| NC_050203.1 | 18795922 | G | A | IndiaCol_PakistanCol |
| NC_050203.1 | 18797140 | C | T | Ethiopia_PakistanCol |
| NC_050203.1 | 18799709 | C | T | Ethiopia_PakistanCol |
| NC_050203.1 | 18912198 | G | C | Ethiopia_PakistanCol |
| NC_050203.1 | 18945403 | T | A | Ethiopia_PakistanCol |
| NC_050203.1 | 18946222 | G | A | Ethiopia_PakistanCol |
| NC_050203.1 | 19940018 | G | C | IndiaCol_PakistanCol |
| NC_050203.1 | 19940018 | G | C | IndiaWT_PakistanCol |
| NC_050203.1 | 19944343 | G | A | IndiaCol_PakistanCol |
| NC_050203.1 | 19949435 | T | C | Ethiopia_PakistanCol |
| NC_050203.1 | 19949435 | T | C | IndiaCol_PakistanCol |
| NC_050203.1 | 19962805 | G | A | Ethiopia_PakistanCol |
| NC_050203.1 | 19962805 | G | A | IndiaWT_PakistanCol |
| NC_050203.1 | 19964732 | A | G | Ethiopia_PakistanCol |
| NC_050203.1 | 19973071 | T | A | IndiaCol_PakistanCol |
| NC_050203.1 | 19973071 | T | A | IndiaWT_PakistanCol |
| NC_050203.1 | 19977374 | T | G | Ethiopia_IndCol |
| NC_050203.1 | 20095317 | A | G | IndiaCol_PakistanCol |
| NC_050203.1 | 20095390 | G | A | IndiaCol_PakistanCol |
| NC_050203.1 | 20095449 | C | T | IndiaCol_PakistanCol |
| NC_050203.1 | 20095449 | C | T | IndiaWT_PakistanCol |
| NC_050203.1 | 20095475 | G | A | IndiaCol_PakistanCol |
| NC_050203.1 | 20095896 | T | C | IndiaCol_PakistanCol |
| NC_050203.1 | 20096565 | A | G | IndiaCol_PakistanCol |
| NC_050203.1 | 20096600 | A | C | IndiaCol_PakistanCol |
| NC_050203.1 | 20096600 | A | G | IndiaCol_PakistanCol |
| NC_050203.1 | 20096616 | A | G | IndiaCol_PakistanCol |
| NC_050203.1 | 20097447 | C | A | IndiaWT_PakistanCol |
| NC_050203.1 | 20099840 | G | A | IndiaCol_PakistanCol |
| NC_050203.1 | 20099850 | A | G | IndiaCol_PakistanCol |
| NC_050203.1 | 20099862 | G | A | IndiaCol_PakistanCol |
| NC_050203.1 | 20099963 | T | C | Ethiopia_IndCol |
| NC_050203.1 | 20099963 | T | C | IndiaCol_IndiaWT |
| NC_050203.1 | 20101356 | A | C | IndiaCol_PakistanCol |
| NC_050203.1 | 20107984 | T | G | IndiaCol_PakistanCol |
| NC_050203.1 | 20108736 | C | T | Ethiopia_PakistanCol |
| NC_050203.1 | 20121958 | C | T | IndiaCol_PakistanCol |
| NC_050203.1 | 20121961 | C | A | IndiaCol_PakistanCol |
| NC_050203.1 | 20121961 | C | A | IndiaWT_PakistanCol |
| NC_050203.1 | 20217354 | G | T | IndiaCol_PakistanCol |
| NC_050203.1 | 20217366 | T | C | IndiaCol_PakistanCol |
| NC_050203.1 | 20224373 | T | C | Ethiopia_PakistanCol |
| NC_050203.1 | 21621917 | C | A | Ethiopia_PakistanCol |
| NC_050203.1 | 24312322 | G | A | Ethiopia_PakistanCol |
| NC_050203.1 | 24312351 | G | A | Ethiopia_PakistanCol |
| NC_050203.1 | 24337555 | A | G | Ethiopia_PakistanCol |
| NC_050203.1 | 24339996 | C | A | Ethiopia_IndCol |
| NC_050203.1 | 24351368 | A | G | Ethiopia_PakistanCol |
| NC_050203.1 | 24351368 | A | G | IndiaWT_PakistanCol |
| NC_050203.1 | 24899868 | T | G | Ethiopia_PakistanCol |
| NC_050203.1 | 26171586 | G | A | IndiaCol_PakistanCol |
| NC_050203.1 | 26171587 | T | A | IndiaCol_PakistanCol |
| NC_050203.1 | 26777797 | A | C | Ethiopia_PakistanCol |
| NC_050203.1 | 29293653 | A | C | Ethiopia_PakistanCol |
| NC_050203.1 | 29425578 | C | T | Ethiopia_PakistanCol |
| NC_050203.1 | 29425578 | C | T | IndiaCol_PakistanCol |
| NC_050203.1 | 29425578 | C | T | IndiaWT_PakistanCol |
| NC_050203.1 | 29425622 | C | T | IndiaCol_PakistanCol |
| NC_050203.1 | 29425622 | C | T | IndiaWT_PakistanCol |
| NC_050203.1 | 29425941 | A | G | IndiaCol_PakistanCol |
| NC_050203.1 | 29429866 | T | C | Ethiopia_PakistanCol |
| NC_050203.1 | 29430362 | C | T | Ethiopia_PakistanCol |
| NC_050203.1 | 29430401 | A | T | Ethiopia_PakistanCol |
| NC_050203.1 | 29430470 | G | A | Ethiopia_PakistanCol |
| NC_050203.1 | 29430655 | C | G | Ethiopia_PakistanCol |
| NC_050203.1 | 29430676 | A | C | Ethiopia_PakistanCol |
| NC_050203.1 | 29478627 | T | C | Ethiopia_IndCol |
| NC_050203.1 | 29503375 | G | A | Ethiopia_PakistanCol |
| NC_050203.1 | 29503577 | G | A | IndiaCol_PakistanCol |
| NC_050203.1 | 29833425 | A | G | IndiaCol_IndiaWT |
| NC_050203.1 | 29837355 | A | G | IndiaWT_PakistanCol |
| NC_050203.1 | 32063894 | G | A | IndiaCol_PakistanCol |
| NC_050203.1 | 32610532 | T | C | Ethiopia_IndCol |
| NC_050203.1 | 32611721 | T | G | Ethiopia_PakistanCol |
| NC_050203.1 | 32611722 | C | T | Ethiopia_PakistanCol |
| NC_050203.1 | 32611728 | A | G | Ethiopia_PakistanCol |
| NC_050203.1 | 32611747 | A | G | Ethiopia_PakistanCol |
| NC_050203.1 | 32611914 | A | T | Ethiopia_PakistanCol |
| NC_050203.1 | 32611914 | A | T | IndiaWT_PakistanCol |
| NC_050203.1 | 32611918 | A | T | Ethiopia_IndCol |
| NC_050203.1 | 32611918 | A | T | Ethiopia_PakistanCol |
| NC_050203.1 | 32611969 | T | C | Ethiopia_PakistanCol |
| NC_050203.1 | 32995806 | T | A | IndiaCol_PakistanCol |
| NC_050203.1 | 32995889 | G | A | Ethiopia_PakistanCol |
| NC_050203.1 | 32995889 | G | A | IndiaCol_PakistanCol |
| NC_050203.1 | 33203463 | T | G | IndiaCol_PakistanCol |
| NC_050203.1 | 33205732 | G | A | Ethiopia_PakistanCol |
| NC_050203.1 | 33205732 | G | A | IndiaCol_PakistanCol |
| NC_050203.1 | 33205732 | G | A | IndiaWT_PakistanCol |
| NC_050203.1 | 33206482 | G | T | Ethiopia_PakistanCol |
| NC_050203.1 | 33222011 | C | T | IndiaCol_PakistanCol |
| NC_050203.1 | 33222011 | C | T | IndiaWT_PakistanCol |
| NC_050203.1 | 33222018 | C | T | IndiaCol_PakistanCol |
| NC_050203.1 | 33222018 | C | T | IndiaWT_PakistanCol |
| NC_050203.1 | 33222637 | C | T | IndiaCol_PakistanCol |
| NC_050203.1 | 33223287 | C | T | IndiaCol_PakistanCol |
| NC_050203.1 | 33621784 | C | T | Ethiopia_IndCol |
| NC_050203.1 | 33621784 | C | T | IndiaCol_PakistanCol |
| NC_050203.1 | 38529303 | G | T | IndiaCol_PakistanCol |
| NC_050203.1 | 38529303 | G | T | IndiaWT_PakistanCol |
| NC_050203.1 | 40659990 | T | C | IndiaCol_PakistanCol |
| NC_050203.1 | 42592751 | T | G | IndiaCol_IndiaWT |
| NC_050203.1 | 42592751 | T | G | IndiaCol_PakistanCol |
| NC_050203.1 | 43661492 | C | T | IndiaWT_PakistanCol |
| NC_050203.1 | 43856722 | T | C | IndiaWT_PakistanCol |
| NC_050203.1 | 46697700 | A | C | Ethiopia_PakistanCol |
| NC_050203.1 | 46697700 | A | C | IndiaWT_PakistanCol |
| NC_050203.1 | 46697720 | T | C | Ethiopia_PakistanCol |
| NC_050203.1 | 46705456 | A | T | Ethiopia_IndCol |
| NC_050203.1 | 49272400 | A | G | Ethiopia_PakistanCol |
| NC_050203.1 | 49272400 | A | G | IndiaCol_PakistanCol |
| NC_050203.1 | 49272400 | A | G | IndiaWT_PakistanCol |
| NC_050203.1 | 50592571 | A | G | IndiaCol_PakistanCol |
| NC_050203.1 | 56801537 | A | T | IndiaCol_PakistanCol |
| NC_050203.1 | 57599805 | A | G | IndiaCol_IndiaWT |
| NC_050203.1 | 58568059 | G | A | IndiaCol_PakistanCol |
| NC_050203.1 | 58568059 | G | A | IndiaWT_PakistanCol |
| NC_050203.1 | 58568061 | A | G | IndiaCol_PakistanCol |
| NC_050203.1 | 58568061 | A | G | IndiaWT_PakistanCol |
| NC_050203.1 | 58994177 | T | A | Ethiopia_IndCol |
| NC_050203.1 | 58994177 | T | G | Ethiopia_IndCol |
| NC_050203.1 | 59349278 | T | C | Ethiopia_PakistanCol |
| NC_050203.1 | 59349278 | T | C | IndiaCol_PakistanCol |
| NC_050203.1 | 59349278 | T | C | IndiaWT_PakistanCol |
| NC_050203.1 | 59354467 | G | A | IndiaCol_PakistanCol |
| NC_050203.1 | 59354686 | A | G | IndiaCol_PakistanCol |
| NC_050203.1 | 59354721 | A | G | IndiaCol_PakistanCol |
| NC_050203.1 | 59354791 | C | G | IndiaCol_PakistanCol |
| NC_050203.1 | 59354791 | C | A | IndiaCol_PakistanCol |
| NC_050203.1 | 59355281 | A | C | IndiaCol_PakistanCol |
| NC_050203.1 | 59362200 | T | A | IndiaCol_PakistanCol |
| NC_050203.1 | 59389529 | A | T | Ethiopia_IndCol |
| NC_050203.1 | 59389529 | A | T | IndiaCol_IndiaWT |
| NC_050203.1 | 59389529 | A | T | IndiaCol_PakistanCol |
| NC_050203.1 | 59389616 | C | T | Ethiopia_IndCol |
| NC_050203.1 | 59389616 | C | T | IndiaCol_IndiaWT |
| NC_050203.1 | 60172336 | T | G | Ethiopia_IndWT |
| NC_050203.1 | 60172336 | T | G | IndiaWT_PakistanCol |
| NC_050203.1 | 60172343 | C | T | Ethiopia_IndWT |
| NC_050203.1 | 60172343 | C | T | IndiaWT_PakistanCol |
| NC_050203.1 | 60172344 | G | T | Ethiopia_IndWT |
| NC_050203.1 | 60172344 | G | T | IndiaWT_PakistanCol |
| NC_050203.1 | 63410054 | A | G | Ethiopia_PakistanCol |
| NC_050203.1 | 67421642 | G | A | Ethiopia_PakistanCol |
| NC_050203.1 | 71554574 | T | A | Ethiopia_IndCol |
| NC_050203.1 | 71554574 | T | A | IndiaCol_PakistanCol |
| NC_050203.1 | 71554593 | G | T | IndiaCol_PakistanCol |
| NC_050203.1 | 72066486 | C | T | IndiaWT_PakistanCol |
| NC_050203.1 | 72068438 | G | A | IndiaWT_PakistanCol |
| NC_050203.1 | 72068483 | G | A | IndiaWT_PakistanCol |
| NC_050203.1 | 72068498 | C | T | IndiaWT_PakistanCol |
| NC_050203.1 | 72767844 | C | A | Ethiopia_PakistanCol |
| NC_050203.1 | 77047273 | T | C | Ethiopia_PakistanCol |
| NC_050203.1 | 77363620 | C | G | Ethiopia_PakistanCol |
| NC_050203.1 | 78782394 | A | C | Ethiopia_PakistanCol |
| NC_050203.1 | 80455444 | C | A | IndiaWT_PakistanCol |
| NC_050203.1 | 80455480 | C | T | IndiaWT_PakistanCol |
| NC_050203.1 | 80455522 | T | A | IndiaWT_PakistanCol |
| NC_050203.1 | 80455540 | T | G | IndiaWT_PakistanCol |
| NC_050203.1 | 80455544 | C | T | IndiaWT_PakistanCol |
| NC_050203.1 | 81339921 | G | C | Ethiopia_PakistanCol |
| NC_050203.1 | 81339921 | G | C | IndiaWT_PakistanCol |
| NC_050203.1 | 81851144 | G | A | IndiaCol_PakistanCol |
| NC_050203.1 | 81851162 | C | T | IndiaCol_PakistanCol |
| NC_050203.1 | 81905852 | C | T | IndiaWT_PakistanCol |
| NC_050203.1 | 81958711 | C | T | Ethiopia_PakistanCol |
| NC_050203.1 | 81961716 | A | G | Ethiopia_IndCol |
| NC_050203.1 | 81966982 | A | G | Ethiopia_PakistanCol |
| NC_050203.1 | 81966982 | A | G | IndiaCol_PakistanCol |
| NC_050203.1 | 81973606 | C | T | IndiaCol_PakistanCol |
| NC_050203.1 | 81979712 | G | A | IndiaCol_PakistanCol |
| NC_050203.1 | 81979715 | C | A | IndiaCol_PakistanCol |
| NC_050203.1 | 81979721 | T | C | IndiaCol_PakistanCol |
| NC_050203.1 | 82147505 | T | C | Ethiopia_PakistanCol |
| NC_050203.1 | 82147505 | T | C | IndiaCol_PakistanCol |
| NC_050203.1 | 82147505 | T | C | IndiaWT_PakistanCol |
| NC_050203.1 | 82147614 | A | C | Ethiopia_PakistanCol |
| NC_050203.1 | 82147614 | A | C | IndiaCol_PakistanCol |
| NC_050203.1 | 82147614 | A | C | IndiaWT_PakistanCol |
| NC_050203.1 | 82147620 | A | G | Ethiopia_PakistanCol |
| NC_050203.1 | 82147620 | A | G | IndiaCol_PakistanCol |
| NC_050203.1 | 82147620 | A | G | IndiaWT_PakistanCol |
| NC_050203.1 | 82147740 | C | G | IndiaCol_PakistanCol |
| NC_050203.1 | 82148774 | T | A | IndiaCol_PakistanCol |
| NC_050203.1 | 82148798 | C | T | IndiaCol_PakistanCol |
| NC_050203.1 | 82148798 | C | T | IndiaWT_PakistanCol |
| NC_050203.1 | 82148938 | G | A | IndiaCol_PakistanCol |
| NC_050203.1 | 82148938 | G | A | IndiaWT_PakistanCol |
| NC_050203.1 | 82151212 | C | A | IndiaCol_PakistanCol |
| NC_050203.1 | 82151212 | C | A | IndiaWT_PakistanCol |
| NC_050203.1 | 82151783 | C | A | IndiaCol_PakistanCol |
| NC_050203.1 | 82151798 | A | T | IndiaCol_PakistanCol |
| NC_050203.1 | 82165342 | A | T | Ethiopia_PakistanCol |
| NC_050203.1 | 82810240 | T | C | IndiaCol_PakistanCol |
| NC_050203.1 | 84384411 | A | G | IndiaWT_PakistanCol |
| NC_050203.1 | 85535111 | C | T | Ethiopia_PakistanCol |
| NC_050203.1 | 85642885 | A | C | IndiaCol_PakistanCol |
| NC_050203.1 | 85643396 | C | T | IndiaCol_PakistanCol |
| NC_050203.1 | 85643411 | T | A | IndiaCol_PakistanCol |
| NC_050203.1 | 85643459 | T | A | IndiaCol_PakistanCol |
| NC_050203.1 | 85643526 | A | C | IndiaCol_PakistanCol |
| NC_050203.1 | 85643528 | T | A | IndiaCol_PakistanCol |
| NC_050203.1 | 85643561 | A | G | IndiaCol_PakistanCol |
| NC_050203.1 | 85643578 | A | T | IndiaCol_PakistanCol |
| NC_050203.1 | 85643590 | C | A | IndiaCol_PakistanCol |
| NC_050203.1 | 85643591 | C | A | IndiaCol_PakistanCol |
| NC_050203.1 | 85643591 | C | G | IndiaCol_PakistanCol |
| NC_050203.1 | 85643594 | G | A | IndiaCol_PakistanCol |
| NC_050203.1 | 85643598 | C | T | IndiaCol_PakistanCol |
| NC_050203.1 | 85643601 | T | A | IndiaCol_PakistanCol |
| NC_050203.1 | 85661836 | A | C | IndiaCol_PakistanCol |
| NC_050203.1 | 85661932 | C | A | IndiaCol_PakistanCol |
| NC_050203.1 | 85661936 | T | C | IndiaCol_PakistanCol |
| NC_050203.1 | 85662228 | T | A | IndiaCol_PakistanCol |
| NC_050203.1 | 86450484 | A | G | IndiaCol_PakistanCol |
| NC_050203.1 | 86461173 | G | C | IndiaCol_PakistanCol |
| NC_050203.1 | 86608239 | T | A | Ethiopia_IndCol |
| NC_050203.1 | 87780569 | T | A | Ethiopia_IndCol |
| NC_050203.1 | 87780569 | T | A | IndiaCol_PakistanCol |
| NC_050203.1 | 87781274 | G | A | Ethiopia_IndCol |
| NC_050203.1 | 87781274 | G | A | IndiaCol_PakistanCol |

**Supplementary Table S3.** Genomic positions with significant XP-EHH scores (>4.0)

| Chromosome | Position | XPEHH  Score | Reference allele | Alternate Allele | Gene name | Population comparison |
| --- | --- | --- | --- | --- | --- | --- |
| 50201 | 9465180 | -5.005665328 | A | C | sodium channel protein Nach | Ethiopia\|India Wildtype |
| 50201 | 9465180 | 5.005665328 | A | C | sodium channel protein Nach | Ethiopia\|India Wildtype |
| 50201 | 9465189 | -4.444481662 | G | A | sodium channel protein Nach | Ethiopia\|India Wildtype |
| 50201 | 9465189 | 4.444481662 | G | A | sodium channel protein Nach | Ethiopia\|India Wildtype |
| 50201 | 9467621 | -4.616753578 | T | G | sodium channel protein Nach | Ethiopia\|India Wildtype |
| 50201 | 9467621 | 4.616753578 | T | G | sodium channel protein Nach | Ethiopia\|India Wildtype |
| 50201 | 9480436 | -4.458442882 | A | G | sodium channel protein Nach | Ethiopia\|India Wildtype |
| 50201 | 9480436 | 4.458442882 | A | G | sodium channel protein Nach | Ethiopia\|India Wildtype |
| 50201 | 9480437 | -4.458442882 | A | C | sodium channel protein Nach | Ethiopia\|India Wildtype |
| 50201 | 9480437 | 4.458442882 | A | C | sodium channel protein Nach | Ethiopia\|India Wildtype |
| 50201 | 13100960 | -4.709717919 | T | G | AF4/FMR2 family member lilli-like | India Colony\|Ethiopia |
| 50201 | 13100960 | 4.709717919 | T | G | AF4/FMR2 family member lilli-like | India Colony\|Ethiopia |
| 50202 | 1055305 | -5.113814473 | C | A | POU domain, class 6, transcription factor 2 | India Colony\|India Wildtype |
| 50202 | 1055305 | 5.113814473 | C | A | POU domain, class 6, transcription factor 2 | India Colony\|India Wildtype |
| 50202 | 2083985 | -4.55636738 | C | T | endoribonuclease Dicer | Ethiopia\|India Wildtype |
| 50202 | 2083985 | 4.55636738 | C | T | endoribonuclease Dicer | Ethiopia\|India Wildtype |
| 50202 | 4742060 | 4.599331789 | C | A | uncharacterized LOC118508163 | Ethiopia\|India Wildtype |
| 50202 | 4742060 | -4.599331789 | C | A | uncharacterized LOC118508163 | Ethiopia\|India Wildtype |
| 50202 | 5592348 | -4.530278196 | T | A | limbic system-associated membrane protein | India Colony\|Ethiopia |
| 50202 | 5592348 | 4.530278196 | T | A | limbic system-associated membrane protein | India Colony\|Ethiopia |
| 50202 | 12062186 | -4.727903879 | G | T | speract receptor | India Colony\|India Wildtype |
| 50202 | 12062186 | 4.727903879 | G | T | speract receptor | India Colony\|India Wildtype |
| 50202 | 12064274 | -4.521993755 | C | T | speract receptor | India Colony\|India Wildtype |
| 50202 | 12064274 | 4.521993755 | C | T | speract receptor | India Colony\|India Wildtype |
| 50202 | 12064324 | -5.091355371 | C | T | speract receptor | India Colony\|India Wildtype |
| 50202 | 12064324 | 5.091355371 | C | T | speract receptor | India Colony\|India Wildtype |
| 50202 | 17196920 | -4.762553453 | T | G | meiotic recombination protein SPO11 | Ethiopia\|India Wildtype |
| 50202 | 17196920 | 4.762553453 | T | G | meiotic recombination protein SPO11 | Ethiopia\|India Wildtype |
| 50202 | 21207678 | -4.516864651 | C | A | ankyrin repeat domain-containing protein 17 | India Colony\|India Wildtype |
| 50202 | 21207678 | 4.516864651 | C | A | ankyrin repeat domain-containing protein 17 | India Colony\|India Wildtype |
| 50202 | 21207681 | -5.094011005 | C | T | ankyrin repeat domain-containing protein 17 | India Colony\|India Wildtype |
| 50202 | 21207681 | 5.094011005 | C | T | ankyrin repeat domain-containing protein 17 | India Colony\|India Wildtype |
| 50202 | 21356934 | -4.510156561 | G | A | ankyrin repeat domain-containing protein 17 | India Colony\|India Wildtype |
| 50202 | 21356934 | 4.510156561 | G | A | ankyrin repeat domain-containing protein 17 | India Colony\|India Wildtype |
| 50202 | 21385853 | -5.059016074 | A | G | ankyrin repeat domain-containing protein 17 | India Colony\|India Wildtype |
| 50202 | 21385853 | 5.059016074 | A | G | ankyrin repeat domain-containing protein 17 | India Colony\|India Wildtype |
| 50202 | 25400970 | -4.421035661 | T | C | protein germ cell-less | India Colony\|India Wildtype |
| 50202 | 25400970 | 4.421035661 | T | C | protein germ cell-less | India Colony\|India Wildtype |
| 50202 | 27087057 | -4.537178445 | G | A | growth factor receptor-bound protein 2 | India Colony\|India Wildtype |
| 50202 | 27087057 | 4.537178445 | G | A | growth factor receptor-bound protein 2 | India Colony\|India Wildtype |
| 50202 | 35316698 | -4.497163664 | G | C | uncharacterized LOC118506318 | India Colony\|India Wildtype |
| 50202 | 35316698 | 4.497163664 | G | C | uncharacterized LOC118506318 | India Colony\|India Wildtype |
| 50202 | 36554735 | -4.670777572 | A | T | N-acetylgalactosaminyltransferase 4-like | India Colony\|India Wildtype |
| 50202 | 36554735 | 4.670777572 | A | T | N-acetylgalactosaminyltransferase 4-like | India Colony\|India Wildtype |
| 50202 | 36622449 | -4.425557617 | A | C | protein gooseberry-neuro | India Colony\|India Wildtype |
| 50202 | 36622449 | 4.425557617 | A | C | protein gooseberry-neuro | India Colony\|India Wildtype |
| 50202 | 36622455 | -4.464974184 | C | G | protein gooseberry-neuro | India Colony\|India Wildtype |
| 50202 | 36622455 | 4.464974184 | C | G | protein gooseberry-neuro | India Colony\|India Wildtype |
| 50202 | 37475265 | -4.453004824 | G | T | F-box/SPRY domain-containing protein 1 | India Colony\|India Wildtype |
| 50202 | 37475265 | 4.453004824 | G | T | F-box/SPRY domain-containing protein 1 | India Colony\|India Wildtype |
| 50202 | 38473284 | -4.708242107 | C | T | homeobox protein abdominal-B-like | India Colony\|India Wildtype |
| 50202 | 38473284 | 4.708242107 | C | T | homeobox protein abdominal-B-like | India Colony\|India Wildtype |
| 50202 | 39404435 | -4.555763217 | T | C | uncharacterized LOC118507833 | India Colony\|India Wildtype |
| 50202 | 39404435 | 4.555763217 | T | C | uncharacterized LOC118507833 | India Colony\|India Wildtype |
| 50202 | 39818059 | -4.595908924 | C | T | uncharacterized LOC118507877 | India Colony\|India Wildtype |
| 50202 | 39818059 | 4.595908924 | C | T | uncharacterized LOC118507877 | India Colony\|India Wildtype |
| 50202 | 41527499 | -4.41754895 | C | T | dopamine D2-like recepto | India Colony\|India Wildtype |
| 50202 | 41527499 | 4.41754895 | C | T | dopamine D2-like recepto | India Colony\|India Wildtype |
| 50202 | 41861815 | -4.796144402 | T | C | lethal(2) giant larvae protein | India Colony\|India Wildtype |
| 50202 | 41861815 | 4.796144402 | T | C | lethal(2) giant larvae protein | India Colony\|India Wildtype |
| 50202 | 48706614 | -4.800396475 | C | G | centrosomin | India Colony\|India Wildtype |
| 50202 | 48706614 | 4.800396475 | C | G | centrosomin | India Colony\|India Wildtype |
| 50202 | 52988816 | -4.641803851 | T | A | cell wall protein AWA1 | India Colony\|India Wildtype |
| 50202 | 52988816 | 4.641803851 | T | A | cell wall protein AWA1 | India Colony\|India Wildtype |
| 50202 | 52996720 | -4.562666071 | G | A | cell wall protein AWA1 | India Colony\|India Wildtype |
| 50202 | 52996720 | 4.562666071 | G | A | cell wall protein AWA1 | India Colony\|India Wildtype |
| 50202 | 53039951 | -5.773605556 | C | A | cell wall protein AWA1 | India Colony\|India Wildtype |
| 50202 | 53039951 | 5.773605556 | C | A | cell wall protein AWA1 | India Colony\|India Wildtype |
| 50202 | 53047183 | -4.441675247 | C | T | cell wall protein AWA1 | India Colony\|India Wildtype |
| 50202 | 53047183 | 4.441675247 | C | T | cell wall protein AWA1 | India Colony\|India Wildtype |
| 50202 | 59287030 | -4.516720667 | C | A | nicotinamidase | India Colony\|Ethiopia |
| 50202 | 59287030 | 4.516720667 | C | A | nicotinamidase | India Colony\|Ethiopia |
| 50202 | 59667235 | -4.431415707 | A | G | cubilin | India Colony\|Ethiopia |
| 50202 | 59667235 | 4.431415707 | A | G | cubilin | India Colony\|Ethiopia |
| 50202 | 60835853 | -4.475126562 | C | T | pre-mRNA 3' end processing protein WDR33 | India Colony\|Ethiopia |
| 50202 | 60835853 | 4.475126562 | C | T | pre-mRNA 3' end processing protein WDR33 | India Colony\|Ethiopia |
| 50202 | 61257455 | -4.509738748 | T | C | TBC1 domain family member whacked | India Colony\|India Wildtype |
| 50202 | 61257455 | 4.509738748 | T | C | TBC1 domain family member whacked | India Colony\|India Wildtype |
| 50202 | 61257491 | -5.300927412 | G | A | TBC1 domain family member whacked | India Colony\|India Wildtype |
| 50202 | 61257491 | 5.300927412 | G | A | TBC1 domain family member whacked | India Colony\|India Wildtype |
| 50202 | 61259531 | -4.517148456 | T | G | TBC1 domain family member whacked | India Colony\|India Wildtype |
| 50202 | 61259531 | 4.517148456 | T | G | TBC1 domain family member whacked | India Colony\|India Wildtype |
| 50202 | 68720664 | -5.304395585 | G | A | acetylcholine receptor subunit beta-like 2 | Ethiopia\|India Wildtype |
| 50202 | 68720664 | 5.304395585 | G | A | acetylcholine receptor subunit beta-like 2 | Ethiopia\|India Wildtype |
| 50202 | 68720666 | -5.304395585 | A | G | acetylcholine receptor subunit beta-like 2 | Ethiopia\|India Wildtype |
| 50202 | 68720666 | 5.304395585 | A | G | acetylcholine receptor subunit beta-like 2 | Ethiopia\|India Wildtype |
| 50202 | 76811040 | -4.434506507 | T | C | hemicentin-1 | India Colony\|Ethiopia |
| 50202 | 76811040 | -5.089076007 | T | C | hemicentin-1 | India Colony\|India Wildtype |
| 50202 | 76811040 | 4.434506507 | T | C | hemicentin-1 | India Colony\|Ethiopia |
| 50202 | 76811040 | 5.089076007 | T | C | hemicentin-1 | India Colony\|India Wildtype |
| 50202 | 76811043 | -5.495219203 | C | T | hemicentin-1 | India Colony\|Ethiopia |
| 50202 | 76811043 | -6.217604889 | C | T | hemicentin-1 | India Colony\|India Wildtype |
| 50202 | 76811043 | 5.495219203 | C | T | hemicentin-1 | India Colony\|Ethiopia |
| 50202 | 76811043 | 6.217604889 | C | T | hemicentin-1 | India Colony\|India Wildtype |
| 50202 | 76811044 | -5.394962388 | G | A | hemicentin-1 | India Colony\|Ethiopia |
| 50202 | 76811044 | -6.110186512 | G | A | hemicentin-1 | India Colony\|India Wildtype |
| 50202 | 76811044 | 5.394962388 | G | A | hemicentin-1 | India Colony\|Ethiopia |
| 50202 | 76811044 | 6.110186512 | G | A | hemicentin-1 | India Colony\|India Wildtype |
| 50202 | 76811048 | -5.97789318 | C | T | hemicentin-1 | India Colony\|India Wildtype |
| 50202 | 76811048 | 5.97789318 | C | T | hemicentin-1 | India Colony\|India Wildtype |
| 50202 | 76811059 | -4.8361411 | A | G | hemicentin-1 | India Colony\|India Wildtype |
| 50202 | 76811059 | 4.8361411 | A | G | hemicentin-1 | India Colony\|India Wildtype |
| 50202 | 76811061 | -4.927425796 | T | G | hemicentin-1 | India Colony\|India Wildtype |
| 50202 | 76811061 | 4.927425796 | T | G | hemicentin-1 | India Colony\|India Wildtype |
| 50202 | 78746627 | -4.445383926 | G | T | putative transcription factor capicua | India Colony\|India Wildtype |
| 50202 | 78746627 | 4.445383926 | G | T | putative transcription factor capicua | India Colony\|India Wildtype |
| 50202 | 78964825 | -4.637697645 | G | T | serine/threonine-protein phosphatase PP1-beta catalytic subuni | India Colony\|Ethiopia |
| 50202 | 78964825 | 4.637697645 | G | T | serine/threonine-protein phosphatase PP1-beta catalytic subuni | India Colony\|Ethiopia |
| 50202 | 80145364 | -4.551285507 | G | T | Kv channel-interacting protein 1 | Ethiopia\|India Wildtype |
| 50202 | 80145364 | 4.551285507 | G | T | Kv channel-interacting protein 1 | Ethiopia\|India Wildtype |
| 50202 | 80281624 | -5.668252926 | C | A | angiopoietin-related protein 1-like | India Colony\|India Wildtype |
| 50202 | 80281624 | 5.668252926 | C | A | angiopoietin-related protein 1-like | India Colony\|India Wildtype |
| 50202 | 80281625 | -5.755461491 | G | A | angiopoietin-related protein 1-like | India Colony\|India Wildtype |
| 50202 | 80281625 | 5.755461491 | G | A | angiopoietin-related protein 1-like | India Colony\|India Wildtype |
| 50202 | 80281630 | -5.180678507 | G | T | angiopoietin-related protein 1-like | India Colony\|India Wildtype |
| 50202 | 80281630 | 5.180678507 | G | T | angiopoietin-related protein 1-like | India Colony\|India Wildtype |
| 50202 | 80295481 | -4.573458762 | C | T | connectin-like | India Colony\|India Wildtype |
| 50202 | 80295481 | 4.573458762 | C | T | connectin-like | India Colony\|India Wildtype |
| 50202 | 80374058 | -4.830799954 | A | C | connectin-like | India Colony\|India Wildtype |
| 50202 | 80374058 | 4.830799954 | A | C | connectin-like | India Colony\|India Wildtype |
| 50202 | 80374072 | -4.462623292 | A | T | connectin-like | India Colony\|India Wildtype |
| 50202 | 80374072 | 4.462623292 | A | T | connectin-like | India Colony\|India Wildtype |
| 50202 | 80377015 | -4.552850054 | T | C | connectin-like | India Colony\|India Wildtype |
| 50202 | 80377015 | 4.552850054 | T | C | connectin-like | India Colony\|India Wildtype |
| 50202 | 80377018 | -4.55744321 | T | G | connectin-like | India Colony\|India Wildtype |
| 50202 | 80377018 | 4.55744321 | T | G | connectin-like | India Colony\|India Wildtype |
| 50202 | 80378945 | -4.680565057 | T | G | connectin-like | India Colony\|India Wildtype |
| 50202 | 80378945 | 4.680565057 | T | G | connectin-like | India Colony\|India Wildtype |
| 50202 | 80378948 | -4.612051821 | A | T | connectin-like | India Colony\|India Wildtype |
| 50202 | 80378948 | 4.612051821 | A | T | connectin-like | India Colony\|India Wildtype |
| 50202 | 80385412 | -4.441177488 | A | G | connectin-like | India Colony\|India Wildtype |
| 50202 | 80385412 | 4.441177488 | A | G | connectin-like | India Colony\|India Wildtype |
| 50202 | 85249950 | -4.441874871 | A | T | CCA tRNA nucleotidyltransferase 1, mitochondrial | India Colony\|Ethiopia |
| 50202 | 85249950 | 4.441874871 | A | T | CCA tRNA nucleotidyltransferase 1, mitochondrial | India Colony\|Ethiopia |
| 50202 | 86768223 | -4.816762081 | A | T | coiled-coil domain-containing protein lobo | India Colony\|India Wildtype |
| 50202 | 86768223 | 4.816762081 | A | T | coiled-coil domain-containing protein lobo | India Colony\|India Wildtype |
| 50202 | 86768493 | -4.595960863 | A | G | coiled-coil domain-containing protein lobo | India Colony\|India Wildtype |
| 50202 | 86768493 | 4.595960863 | A | G | coiled-coil domain-containing protein lobo | India Colony\|India Wildtype |
| 50202 | 86771047 | -4.601367228 | A | G | coiled-coil domain-containing protein lobo | India Colony\|India Wildtype |
| 50202 | 86771047 | 4.601367228 | A | G | coiled-coil domain-containing protein lobo | India Colony\|India Wildtype |
| 50202 | 86771072 | -4.674419435 | T | A | coiled-coil domain-containing protein lobo | India Colony\|India Wildtype |
| 50202 | 86771072 | 4.674419435 | T | A | coiled-coil domain-containing protein lobo | India Colony\|India Wildtype |
| 50202 | 86771101 | -4.556824774 | T | C | coiled-coil domain-containing protein lobo | India Colony\|India Wildtype |
| 50202 | 86771101 | 4.556824774 | T | C | coiled-coil domain-containing protein lobo | India Colony\|India Wildtype |
| 50202 | 86771140 | -4.560784184 | C | A | coiled-coil domain-containing protein lobo | India Colony\|India Wildtype |
| 50202 | 86771140 | 4.560784184 | C | A | coiled-coil domain-containing protein lobo | India Colony\|India Wildtype |
| 50202 | 90739286 | -4.545661488 | A | T | E3 ubiquitin-protein ligase TRIM33 | India Colony\|India Wildtype |
| 50202 | 90739286 | 4.545661488 | A | T | E3 ubiquitin-protein ligase TRIM33 | India Colony\|India Wildtype |
| 50203 | 53153112 | -4.817793589 | C | A | 1-phosphatidylinositol 4,5-bisphosphate phosphodiesterase classes I and II | India Colony\|Ethiopia |
| 50203 | 53153113 | -4.542803026 | T | C | 1-phosphatidylinositol 4,5-bisphosphate phosphodiesterase classes I and II | India Colony\|Ethiopia |
| 50203 | 53153112 | 4.817793589 | C | A | 1-phosphatidylinositol 4,5-bisphosphate phosphodiesterase classes I and II | India Colony\|Ethiopia |
| 50203 | 53153113 | 4.542803026 | T | C | 1-phosphatidylinositol 4,5-bisphosphate phosphodiesterase classes I and II | India Colony\|Ethiopia |
| 50203 | 31749273 | -4.569502324 | C | T | potassium voltage-gated channel subfamily KQT member 1-like | India Colony\|India Wildtype |
| 50203 | 31749273 | 4.569502324 | C | T | potassium voltage-gated channel subfamily KQT member 1-like | India Colony\|India Wildtype |
| 50203 | 31873994 | -4.577619538 | A | G | potassium voltage-gated channel subfamily KQT member 1-like | India Colony\|India Wildtype |
| 50203 | 31874076 | -4.909721832 | G | A | potassium voltage-gated channel subfamily KQT member 1-like | India Colony\|India Wildtype |
| 50203 | 31890671 | -4.710033064 | G | A | potassium voltage-gated channel subfamily KQT member 1-like | India Colony\|India Wildtype |
| 50203 | 31873994 | 4.577619538 | A | G | potassium voltage-gated channel subfamily KQT member 1-like | India Colony\|India Wildtype |
| 50203 | 31874076 | 4.909721832 | G | A | potassium voltage-gated channel subfamily KQT member 1-like | India Colony\|India Wildtype |
| 50203 | 31890671 | 4.710033064 | G | A | potassium voltage-gated channel subfamily KQT member 1-like | India Colony\|India Wildtype |
| 50203 | 36632733 | -4.451344621 | A | G | hillarin | India Colony\|India Wildtype |
| 50203 | 36632739 | -4.427059512 | T | C | hillarin | India Colony\|India Wildtype |
| 50203 | 36632788 | -4.709684319 | G | A | hillarin | India Colony\|India Wildtype |
| 50203 | 36632821 | -4.433101043 | G | A | hillarin | India Colony\|India Wildtype |
| 50203 | 36632823 | -4.531065587 | A | G | hillarin | India Colony\|India Wildtype |
| 50203 | 36632827 | -4.446273504 | G | A | hillarin | India Colony\|India Wildtype |
| 50203 | 36632733 | 4.451344621 | A | G | hillarin | India Colony\|India Wildtype |
| 50203 | 36632739 | 4.427059512 | T | C | hillarin | India Colony\|India Wildtype |
| 50203 | 36632788 | 4.709684319 | G | A | hillarin | India Colony\|India Wildtype |
| 50203 | 36632821 | 4.433101043 | G | A | hillarin | India Colony\|India Wildtype |
| 50203 | 36632823 | 4.531065587 | A | G | hillarin | India Colony\|India Wildtype |
| 50203 | 36632827 | 4.446273504 | G | A | hillarin | India Colony\|India Wildtype |
| 50203 | 37105747 | -4.463267349 | A | G | ankyrin repeat domain-containing protein SOWAHB | India Colony\|Ethiopia |
| 50203 | 37127258 | -4.53341508 | G | A | ankyrin repeat domain-containing protein SOWAHB | India Colony\|India Wildtype |
| 50203 | 37105747 | 4.463267349 | A | G | ankyrin repeat domain-containing protein SOWAHB | India Colony\|Ethiopia |
| 50203 | 37127258 | 4.53341508 | G | A | ankyrin repeat domain-containing protein SOWAHB | India Colony\|India Wildtype |
| 50203 | 36445500 | -4.575575657 | A | T | uncharacterized LOC118509705 ( | India Colony\|Ethiopia |
| 50203 | 36445500 | 4.575575657 | A | T | uncharacterized LOC118509705 ( | India Colony\|Ethiopia |
| 50203 | 28008767 | -5.283229508 | C | T | uncharacterized LOC118510096 | India Colony\|India Wildtype |
| 50203 | 28008767 | 5.283229508 | C | T | uncharacterized LOC118510096 | India Colony\|India Wildtype |
| 50203 | 5005839 | -4.459142803 | A | C | DNA N6-methyl adenine demethylase | India Colony\|Ethiopia |
| 50203 | 5005845 | -4.460652716 | G | T | DNA N6-methyl adenine demethylase | India Colony\|Ethiopia |
| 50203 | 5005839 | 4.459142803 | A | C | DNA N6-methyl adenine demethylase | India Colony\|Ethiopia |
| 50203 | 5005845 | 4.460652716 | G | T | DNA N6-methyl adenine demethylase | India Colony\|Ethiopia |
| 50203 | 7684853 | -4.521264918 | T | A | neogenin | India Colony\|India Wildtype |
| 50203 | 7684853 | 4.521264918 | T | A | neogenin | India Colony\|India Wildtype |
| 50203 | 7808325 | -4.695531329 | G | A | uncharacterized LOC118510486 | India Colony\|India Wildtype |
| 50203 | 7808325 | 4.695531329 | G | A | uncharacterized LOC118510486 | India Colony\|India Wildtype |
| 50203 | 7822198 | -4.437732051 | C | T | putative mediator of RNA polymerase II transcription subunit 26 | India Colony\|Ethiopia |
| 50203 | 7822268 | -4.681329214 | G | C | putative mediator of RNA polymerase II transcription subunit 26 | India Colony\|Ethiopia |
| 50203 | 7822328 | -4.458668577 | C | A | putative mediator of RNA polymerase II transcription subunit 26 | India Colony\|Ethiopia |
| 50203 | 7822268 | -4.852902721 | G | C | putative mediator of RNA polymerase II transcription subunit 26 | India Colony\|India Wildtype |
| 50203 | 7822328 | -4.613152098 | C | A | putative mediator of RNA polymerase II transcription subunit 26 | India Colony\|India Wildtype |
| 50203 | 7832999 | -4.542523371 | C | A | putative mediator of RNA polymerase II transcription subunit 26 | India Colony\|India Wildtype |
| 50203 | 7822198 | 4.437732051 | C | T | putative mediator of RNA polymerase II transcription subunit 26 | India Colony\|Ethiopia |
| 50203 | 7822268 | 4.681329214 | G | C | putative mediator of RNA polymerase II transcription subunit 26 | India Colony\|Ethiopia |
| 50203 | 7822328 | 4.458668577 | C | A | putative mediator of RNA polymerase II transcription subunit 26 | India Colony\|Ethiopia |
| 50203 | 7822268 | 4.852902721 | G | C | putative mediator of RNA polymerase II transcription subunit 26 | India Colony\|India Wildtype |
| 50203 | 7822328 | 4.613152098 | C | A | putative mediator of RNA polymerase II transcription subunit 26 | India Colony\|India Wildtype |
| 50203 | 7832999 | 4.542523371 | C | A | putative mediator of RNA polymerase II transcription subunit 26 | India Colony\|India Wildtype |
| 50203 | 7853974 | -4.435484555 | T | A | ATPase family AAA domain-containing protein 5 | India Colony\|India Wildtype |
| 50203 | 7853974 | 4.435484555 | T | A | ATPase family AAA domain-containing protein 5 | India Colony\|India Wildtype |
| 50203 | 7858613 | -4.653240286 | G | A | cytochrome b5-like | India Colony\|India Wildtype |
| 50203 | 7858613 | 4.653240286 | G | A | cytochrome b5-like | India Colony\|India Wildtype |
| 50203 | 8485166 | -5.763886396 | T | G | glutamate receptor 1-like | India Colony\|India Wildtype |
| 50203 | 8485168 | -5.90966027 | A | G | glutamate receptor 1-like | India Colony\|India Wildtype |
| 50203 | 8485172 | -5.412882141 | T | G | glutamate receptor 1-like | India Colony\|India Wildtype |
| 50203 | 8485166 | 5.763886396 | T | G | glutamate receptor 1-like | India Colony\|India Wildtype |
| 50203 | 8485168 | 5.90966027 | A | G | glutamate receptor 1-like | India Colony\|India Wildtype |
| 50203 | 8485171 | 5.810627055 | G | C | glutamate receptor 1-like | India Colony\|India Wildtype |
| 50203 | 8485172 | 5.412882141 | T | G | glutamate receptor 1-like | India Colony\|India Wildtype |
| 50203 | 77326077 | -4.419417499 | C | A | P protein | India Colony\|India Wildtype |
| 50203 | 77326077 | 4.419417499 | C | A | P protein | India Colony\|India Wildtype |
| 50203 | 73673505 | -4.535296303 | A | G | apoptosis-resistant E3 ubiquitin protein ligase 1 | India Colony\|India Wildtype |
| 50203 | 73673505 | 4.535296303 | A | G | apoptosis-resistant E3 ubiquitin protein ligase 1 | India Colony\|India Wildtype |
| 50203 | 15276293 | -4.680159147 | T | G | trithorax group protein osa | India Colony\|India Wildtype |
| 50203 | 15276293 | 4.680159147 | T | G | trithorax group protein osa | India Colony\|India Wildtype |
| 50203 | 2895486 | -4.590372242 | C | T | uncharacterized LOC118511244 | India Colony\|Ethiopia |
| 50203 | 2895486 | 4.590372242 | C | T | uncharacterized LOC118511244 | India Colony\|Ethiopia |
| 50203 | 46918232 | -4.530998326 | T | G | serine-rich adhesin for platelets-like | India Colony\|India Wildtype |
| 50203 | 46918232 | 4.530998326 | T | G | serine-rich adhesin for platelets-like | India Colony\|India Wildtype |
| 50203 | 27625615 | -4.475598481 | G | A | dead ringer-like | India Colony\|India Wildtype |
| 50203 | 27625615 | 4.475598481 | G | A | dead ringer-like | India Colony\|India Wildtype |
| 50203 | 65163599 | -4.692754597 | C | A | paxillin | India Colony\|Ethiopia |
| 50203 | 65163599 | 4.692754597 | C | A | paxillin | India Colony\|Ethiopia |
| 50203 | 40136361 | -4.903095958 | A | G | myb-like protein Q | India Colony\|India Wildtype |
| 50203 | 40136361 | 4.903095958 | A | G | myb-like protein Q | India Colony\|India Wildtype |
| 50203 | 40679757 | -4.444395417 | C | T | NACHT domain- and WD repeat-containing protein 1 | India Colony\|Ethiopia |
| 50203 | 40679757 | 4.444395417 | C | T | NACHT domain- and WD repeat-containing protein 1 | India Colony\|Ethiopia |
| 50203 | 1538814 | 4.710636611 | C | T | lysophospholipid acyltransferase 1 | Ethiopia\|India Wildtype |
| 50203 | 1538814 | -4.710636611 | C | T | lysophospholipid acyltransferase 1 | Ethiopia\|India Wildtype |
| 50203 | 1445974 | -4.804446916 | A | C | uncharacterized LOC118512080 | India Colony\|India Wildtype |
| 50203 | 1445974 | 4.804446916 | A | C | uncharacterized LOC118512080 | India Colony\|India Wildtype |
| 50203 | 69463786 | -4.521174362 | C | T | T-box protein H15-like | India Colony\|India Wildtype |
| 50203 | 69463786 | 4.521174362 | C | T | T-box protein H15-like | India Colony\|India Wildtype |
| 50203 | 15561768 | -4.709458944 | A | G | platelet binding protein GspB | India Colony\|Pakistan Colony |
| 50203 | 15561771 | -4.610603285 | A | G | platelet binding protein GspB | India Colony\|Pakistan Colony |
| 50203 | 15561772 | -4.565390713 | G | A | platelet binding protein GspB | India Colony\|Pakistan Colony |
| 50203 | 15561775 | -4.422688562 | C | G | platelet binding protein GspB | India Colony\|Pakistan Colony |
| 50203 | 15561776 | -4.556652629 | A | T | platelet binding protein GspB | India Colony\|Pakistan Colony |
| 50203 | 15561768 | -4.478021367 | A | G | platelet binding protein GspB | India Colony\|India Wildtype |
| 50203 | 15561768 | 4.709458944 | A | G | platelet binding protein GspB | India Colony\|Pakistan Colony |
| 50203 | 15561771 | 4.610603285 | A | G | platelet binding protein GspB | India Colony\|Pakistan Colony |
| 50203 | 15561772 | 4.565390713 | G | A | platelet binding protein GspB | India Colony\|Pakistan Colony |
| 50203 | 15561775 | 4.422688562 | C | G | platelet binding protein GspB | India Colony\|Pakistan Colony |
| 50203 | 15561776 | 4.556652629 | A | T | platelet binding protein GspB | India Colony\|Pakistan Colony |
| 50203 | 15561768 | 4.478021367 | A | G | platelet binding protein GspB | India Colony\|India Wildtype |
| 50203 | 54844619 | -4.474442724 | A | G | protein outspread | India Colony\|Ethiopia |
| 50203 | 54844623 | -4.608394092 | C | T | protein outspread | India Colony\|Ethiopia |
| 50203 | 54844631 | -4.634456523 | T | C | protein outspread | India Colony\|Ethiopia |
| 50203 | 54844619 | 4.474442724 | A | G | protein outspread | India Colony\|Ethiopia |
| 50203 | 54844623 | 4.608394092 | C | T | protein outspread | India Colony\|Ethiopia |
| 50203 | 54844631 | 4.634456523 | T | C | protein outspread | India Colony\|Ethiopia |
| 50203 | 71907635 | -4.421811549 | T | G | signal-induced proliferation-associated 1-like protein 2 | Ethiopia\|India Wildtype |
| 50203 | 71907635 | 4.421811549 | T | G | signal-induced proliferation-associated 1-like protein 2 | Ethiopia\|India Wildtype |
| 50203 | 17396549 | -4.467277364 | G | C | AF4/FMR2 family member lilli-like | India Colony\|Ethiopia |
| 50203 | 17396549 | 4.467277364 | G | C | AF4/FMR2 family member lilli-like | India Colony\|Ethiopia |
| 50203 | 86803105 | -4.443099736 | T | G | F-box/LRR-repeat protein 20 | India Colony\|India Wildtype |
| 50203 | 86803105 | 4.443099736 | T | G | F-box/LRR-repeat protein 20 | India Colony\|India Wildtype |
| 50203 | 12067769 | -4.689398922 | C | T | polypeptide N-acetylgalactosaminyltransferase | India Colony\|India Wildtype |
| 50203 | 12067783 | -4.486559527 | T | A | polypeptide N-acetylgalactosaminyltransferase | India Colony\|India Wildtype |
| 50203 | 12067769 | 4.689398922 | C | T | polypeptide N-acetylgalactosaminyltransferase | India Colony\|India Wildtype |
| 50203 | 12067783 | 4.486559527 | T | A | polypeptide N-acetylgalactosaminyltransferase | India Colony\|India Wildtype |
| 50203 | 12052275 | -4.529419893 | A | G | uncharacterized LOC118514496 | India Colony\|India Wildtype |
| 50203 | 12052275 | 4.529419893 | A | G | uncharacterized LOC118514496 | India Colony\|India Wildtype |
| 50203 | 67833037 | -4.558188554 | G | T | atrial natriuretic peptide receptor 1 | India Colony\|India Wildtype |
| 50203 | 67833043 | -4.688324454 | T | C | atrial natriuretic peptide receptor 1 | India Colony\|India Wildtype |
| 50203 | 67833116 | -4.997379153 | G | T | atrial natriuretic peptide receptor 1 | India Colony\|India Wildtype |
| 50203 | 67833037 | 4.558188554 | G | T | atrial natriuretic peptide receptor 1 | India Colony\|India Wildtype |
| 50203 | 67833043 | 4.688324454 | T | C | atrial natriuretic peptide receptor 1 | India Colony\|India Wildtype |
| 50203 | 67833116 | 4.997379153 | G | T | atrial natriuretic peptide receptor 1 | India Colony\|India Wildtype |
| 50203 | 50219557 | -4.554666695 | G | C | lipopolysaccharide-induced tumor necrosis factor-alpha factor-like | India Colony\|India Wildtype |
| 50203 | 50219557 | 4.554666695 | G | C | lipopolysaccharide-induced tumor necrosis factor-alpha factor-like | India Colony\|India Wildtype |

**Supplementary Table S4.** Genomic Positions identified as having significant iHS values (>4.0)

| Chromosome | Position | iHS score | Reference Allele | Alternate Allele | Gene Name | Country |
| --- | --- | --- | --- | --- | --- | --- |
| NC_050201 | 4175483 | 4.417535013 | G | A | uncharacterized LOC118508902 | India Wildtype |
| NC_050201 | 4268996 | 4.214704754 | G | A | Paired box protein Pax-1-like | India Wildtype |
| NC_050201 | 4309548 | 3.911837969 | A | T | uncharacterized LOC118505305 | Pakistan Colony |
| NC_050201 | 4375107 | 5.021627962 | T | A | cytosolic carboxypeptidase Nna1-like | Pakistan Colony |
| NC_050201 | 4662227 | 4.087526103 | A | T | eye-specific diacylglycerol kinase | India Wildtype |
| NC_050201 | 8296083 | 3.962427807 | A | T | glutamate receptor ionotropic, kainate 2 | Pakistan Colony |
| NC_050201 | 9492451 | -4.19640634 | A | T | sodium channel protein Nach | India Wildtype |
| NC_050201 | 9506954 | -3.939423346 | A | T | sodium channel protein Nach | India Wildtype |
| NC_050201 | 9764917 | 3.919693415 | A | C | glutamine-rich protein 1-like | Pakistan Colony |
| NC_050201 | 10004067 | 3.904201867 | T | C | irregular chiasm C-roughest protein-like | India Wildtype |
| NC_050201 | 10142985 | 3.949925571 | T | C | irregular chiasm C-roughest protein-like | Ethiopia |
| NC_050201 | 13607505 | 4.157532377 | G | A | gamma-aminobutyric acid receptor subunit beta-like | Pakistan Colony |
| NC_050201 | 18450329 | 4.181925877 | G | C | uncharacterized LOC118504240 | India Wildtype |
| NC_050201 | 19342929 | 4.109573318 | G | A | transmembrane protein fend-like | Ethiopia |
| NC_050202 | 3841619 | 3.91270372 | G | T | protein O-mannosyl-transferase TMTC1-like | Ethiopia |
| NC_050202 | 4354284 | 4.905194928 | A | T | whirlin | Pakistan Colony |
| NC_050202 | 4559949 | 4.315957516 | C | T | catenin delta-2 | India Wildtype |
| NC_050202 | 8622987 | 4.110314526 | C | T | mucin-19 | India Wildtype |
| NC_050202 | 8625890 | -3.903857472 | A | T | mucin-20 | India Wildtype |
| NC_050202 | 9365806 | 4.149363924 | T | A | mucin-21 | Pakistan Colony |
| NC_050202 | 11828043 | 3.923702497 | A | G | allatotropins-like | Pakistan Colony |
| NC_050202 | 11958206 | -3.995391025 | T | C | mitogen-activated protein kinase p38b-like | India Colony |
| NC_050202 | 12150547 | 4.026924217 | C | T | uncharacterized LOC118506264 | India Wildtype |
| NC_050202 | 12185881 | -4.249589089 | C | A | chromosome transmission fidelity protein 8 homolog | India Colony |
| NC_050202 | 12185890 | -4.198850762 | T | A | chromosome transmission fidelity protein 8 homolog | India Colony |
| NC_050202 | 12194269 | -3.989034762 | C | A | uncharacterized LOC118504111 | India Colony |
| NC_050202 | 12200434 | -4.07995283 | G | A | diacylglycerol lipase-beta-like | India Colony |
| NC_050202 | 12200790 | -4.031529316 | A | T | diacylglycerol lipase-beta-like | India Colony |
| NC_050202 | 12201197 | -4.014513861 | A | C | diacylglycerol lipase-beta-like | India Colony |
| NC_050202 | 12201341 | -3.926954148 | A | C | diacylglycerol lipase-beta-like | India Colony |
| NC_050202 | 12201376 | -3.926954148 | C | T | diacylglycerol lipase-beta-like | India Colony |
| NC_050202 | 12201475 | -4.001863223 | A | G | diacylglycerol lipase-beta-like | India Colony |
| NC_050202 | 12201478 | -4.001863223 | C | A | diacylglycerol lipase-beta-like | India Colony |
| NC_050202 | 12642762 | -4.018322247 | T | C | RYamide receptor | India Colony |
| NC_050202 | 12644238 | -3.964298613 | T | G | RYamide receptor | India Colony |
| NC_050202 | 12644267 | -4.295106802 | G | A | RYamide receptor | India Colony |
| NC_050202 | 12644268 | -4.295106802 | T | G | RYamide receptor | India Colony |
| NC_050202 | 12644301 | -4.092321635 | T | A | RYamide receptor | India Colony |
| NC_050202 | 12644365 | -4.209196531 | A | G | RYamide receptor | India Colony |
| NC_050202 | 12644842 | -4.334685642 | G | A | RYamide receptor | India Colony |
| NC_050202 | 12644896 | -4.040835092 | T | A | RYamide receptor | India Colony |
| NC_050202 | 12889600 | 3.977596391 | T | C | metal cation symporter ZIP14 | India Wildtype |
| NC_050202 | 12895396 | 4.290049612 | T | G | ncharacterized LOC118504108 | India Wildtype |
| NC_050202 | 13191138 | 3.963905776 | G | C | frizzled | India Wildtype |
| NC_050202 | 13366093 | 4.143918801 | A | T | frizzled | India Wildtype |
| NC_050202 | 14666766 | 3.993557519 | T | G | failed axon connections | India Wildtype |
| NC_050202 | 15704203 | -3.946607486 | G | A | nephrin-like | India Wildtype |
| NC_050202 | 16717495 | 4.404005731 | C | T | autophagy protein 5 | India Wildtype |
| NC_050202 | 20647149 | 4.03782534 | G | T | fat-like cadherin-related tumor suppressor homolog | India Wildtype |
| NC_050202 | 20649225 | 4.294889809 | T | A | fat-like cadherin-related tumor suppressor homolog | India Wildtype |
| NC_050202 | 20649447 | 4.144176594 | T | A | fat-like cadherin-related tumor suppressor homolog | India Wildtype |
| NC_050202 | 21018593 | -3.900989708 | T | C | protein commissureless 2 homolog | India Wildtype |
| NC_050202 | 21031290 | 3.948090565 | G | A | protein commissureless 2 homolog | India Wildtype |
| NC_050202 | 21067334 | 4.119638734 | G | A | protein commissureless 2 homolog | India Wildtype |
| NC_050202 | 21138205 | 4.029283251 | T | C | bcl-2-related ovarian killer protein | India Wildtype |
| NC_050202 | 21176386 | 3.959520506 | A | T | bcl-2-related ovarian killer protein | India Wildtype |
| NC_050202 | 21176394 | 4.054232649 | T | C | bcl-2-related ovarian killer protein | India Wildtype |
| NC_050202 | 21176927 | 4.025551715 | T | C | bcl-2-related ovarian killer protein | India Wildtype |
| NC_050202 | 21176953 | -3.918076897 | G | T | bcl-2-related ovarian killer protein | India Wildtype |
| NC_050202 | 21176970 | -3.944567104 | C | G | bcl-2-related ovarian killer protein | India Wildtype |
| NC_050202 | 21177545 | -3.992357658 | T | G | bcl-2-related ovarian killer protein | India Wildtype |
| NC_050202 | 21179525 | -4.187297148 | C | A | bcl-2-related ovarian killer protein | India Wildtype |
| NC_050202 | 21179548 | -4.167388767 | G | A | bcl-2-related ovarian killer protein | India Wildtype |
| NC_050202 | 21180087 | -4.01569493 | A | C | bcl-2-related ovarian killer protein | India Wildtype |
| NC_050202 | 21180144 | -4.137933151 | A | G | bcl-2-related ovarian killer protein | India Wildtype |
| NC_050202 | 21205227 | 4.029746418 | T | A | ankyrin repeat domain-containing protein 17 | India Wildtype |
| NC_050202 | 21279080 | -4.545765773 | G | A | ankyrin repeat domain-containing protein 18 | India Wildtype |
| NC_050202 | 21292595 | -3.955388354 | T | C | ankyrin repeat domain-containing protein 19 | India Wildtype |
| NC_050202 | 21307970 | -3.993412463 | C | T | ankyrin repeat domain-containing protein 20 | India Wildtype |
| NC_050202 | 21354505 | 4.414386719 | A | T | ankyrin repeat domain-containing protein 21 | India Wildtype |
| NC_050202 | 21354532 | 4.40986679 | A | G | ankyrin repeat domain-containing protein 22 | India Wildtype |
| NC_050202 | 21357257 | 4.20016696 | T | A | ankyrin repeat domain-containing protein 23 | India Wildtype |
| NC_050202 | 21357287 | 4.247335484 | T | A | ankyrin repeat domain-containing protein 24 | India Wildtype |
| NC_050202 | 21357289 | -3.923090749 | C | A | ankyrin repeat domain-containing protein 25 | India Wildtype |
| NC_050202 | 21359187 | 4.162991307 | G | A | ankyrin repeat domain-containing protein 26 | India Wildtype |
| NC_050202 | 21359757 | 3.973789522 | C | T | ankyrin repeat domain-containing protein 27 | India Wildtype |
| NC_050202 | 21386839 | -4.457694128 | A | C | ankyrin repeat domain-containing protein 28 | India Wildtype |
| NC_050202 | 21386962 | 3.963112239 | C | T | ankyrin repeat domain-containing protein 29 | India Wildtype |
| NC_050202 | 21454270 | -4.247486938 | A | T | ankyrin repeat domain-containing protein 30 | India Wildtype |
| NC_050202 | 21464830 | 4.366570265 | G | A | ankyrin repeat domain-containing protein 31 | India Wildtype |
| NC_050202 | 21493173 | 3.998336197 | G | C | ankyrin repeat domain-containing protein 32 | India Wildtype |
| NC_050202 | 21493206 | 4.361449229 | A | C | ankyrin repeat domain-containing protein 33 | India Wildtype |
| NC_050202 | 21495232 | 4.281336006 | C | T | ankyrin repeat domain-containing protein 34 | India Wildtype |
| NC_050202 | 21609803 | 3.929065492 | C | T | uncharacterized LOC118504282 | India Wildtype |
| NC_050202 | 21609862 | 4.118706228 | G | T | uncharacterized LOC118504282 | India Wildtype |
| NC_050202 | 21667233 | -4.038983867 | C | A | protein yippee-like CG1530 | India Wildtype |
| NC_050202 | 21667238 | -4.03195732 | T | C | protein yippee-like CG1531 | India Wildtype |
| NC_050202 | 21680339 | -4.283737896 | T | C | protein yippee-like CG15309 | India Wildtype |
| NC_050202 | 21885581 | 4.240972982 | A | G | FAM117B-like | India Wildtype |
| NC_050202 | 21913882 | 4.063191698 | T | A | esterase B1-like | Ethiopia |
| NC_050202 | 21920287 | -4.019263749 | G | C | esterase B1-like | India Wildtype |
| NC_050202 | 23744780 | 4.042925156 | C | A | MOXD1 homolog 2-like | India Wildtype |
| NC_050202 | 23744785 | 3.989137982 | A | T | MOXD1 homolog 2-like | India Wildtype |
| NC_050202 | 24688723 | 3.990574578 | T | C | phosphatidylinositol 4,5-bisphosphate 5-phosphatase A-like | India Wildtype |
| NC_050202 | 25088038 | 4.624869702 | C | T | possible lysine-specific histone demethylase 1-like | India Wildtype |
| NC_050202 | 29856573 | 4.010970168 | G | T | signal recognition particle receptor FtsY | Pakistan Colony |
| NC_050202 | 30191449 | 4.167101577 | T | C | zinc finger protein 37 | India Wildtype |
| NC_050202 | 30193115 | 4.119405823 | T | A | zinc finger protein 38 | India Wildtype |
| NC_050202 | 30380503 | 4.104039059 | A | G | zinc finger protein jing homolog | India Colony |
| NC_050202 | 30380552 | 4.244589467 | C | T | zinc finger protein jing homolog | India Colony |
| NC_050202 | 31620145 | 3.939420201 | C | T | neuroligin-4, X-linked-like | Pakistan Colony |
| NC_050202 | 36394132 | -4.581412325 | G | T | insulin gene enhancer protein ISL-2B-like | India Wildtype |
| NC_050202 | 36501702 | 4.63744568 | G | C | kelch-like protein 20 | India Wildtype |
| NC_050202 | 36502771 | 4.025024777 | C | T | uncharacterized LOC118504780 | India Wildtype |
| NC_050202 | 40843096 | -3.915346753 | A | G | CCR4-NOT transcription complex subunit 6-like | India Wildtype |
| NC_050202 | 41236617 | 3.977888527 | C | T | uncharacterized LOC118506330 | India Wildtype |
| NC_050202 | 41792269 | 3.929862683 | G | T | C1orf43 homolo | India Wildtype |
| NC_050202 | 46037260 | 4.371549941 | T | G | regulator of G-protein signaling loco | India Colony |
| NC_050202 | 46037344 | 4.210359859 | C | T | regulator of G-protein signaling loco | India Colony |
| NC_050202 | 46402717 | 4.089987796 | T | G | lachesin | India Wildtype |
| NC_050202 | 46460544 | 4.077827906 | T | C | uncharacterized protein ZK1073.1 | India Wildtype |
| NC_050202 | 46460719 | 3.897285528 | G | A | uncharacterized protein ZK1073.2 | India Wildtype |
| NC_050202 | 46701868 | 3.993067696 | C | A | mucin-2 | India Wildtype |
| NC_050202 | 47489784 | -4.046485117 | T | A | neuropeptide CCHamide-2 receptor-like | India Colony |
| NC_050202 | 48089519 | 3.919920953 | T | A | 5-aminolevulinate synthase, erythroid-specific, mitochondrial | Ethiopia |
| NC_050202 | 48168576 | 4.030793711 | G | A | protein catecholamines up-like | Ethiopia |
| NC_050202 | 48241216 | 3.936873225 | C | T | protein catecholamines up-like | Ethiopia |
| NC_050202 | 49165265 | 4.602067029 | A | C | synapse-associated protein of 47 kDa | India Wildtype |
| NC_050202 | 49313112 | 4.014681388 | G | T | B-cell receptor CD22 | India Wildtype |
| NC_050202 | 49512432 | 4.196573764 | G | T | keratin-associated protein 19-2-like | India Wildtype |
| NC_050202 | 49512877 | 4.187829679 | C | T | keratin-associated protein 19-2-like | India Wildtype |
| NC_050202 | 49518532 | 3.890875054 | A | T | uncharacterized LOC118504868 | India Wildtype |
| NC_050202 | 50307618 | 4.154260614 | A | T | uncharacterized LOC118502540 | India Wildtype |
| NC_050202 | 50536211 | 3.933050778 | T | C | uncharacterized LOC118502555 | India Wildtype |
| NC_050202 | 50652267 | 4.362916868 | A | G | serine-rich adhesin for platelets-like | India Wildtype |
| NC_050202 | 50660616 | 3.933913389 | C | T | serine-rich adhesin for platelets-like | India Wildtype |
| NC_050202 | 51155667 | 3.97254115 | C | T | pseudouridine-metabolizing bifunctional protein C1861.05 | India Wildtype |
| NC_050202 | 51437917 | 4.091020754 | T | A | flotillin-2 | India Wildtype |
| NC_050202 | 51437918 | 4.045666823 | G | A | flotillin-3 | India Wildtype |
| NC_050202 | 51443555 | 3.97219079 | T | A | flotillin-4 | India Wildtype |
| NC_050202 | 52039954 | 3.973799887 | T | G | maternal protein pumilio | India Wildtype |
| NC_050202 | 52267631 | 4.39529829 | T | G | uncharacterized LOC118502710 | India Wildtype |
| NC_050202 | 52611512 | 4.518530906 | C | A | polypyrimidine tract-binding protein 1 | India Wildtype |
| NC_050202 | 53006707 | -3.954018966 | G | A | cell wall protein AWA | India Wildtype |
| NC_050202 | 53010391 | 3.956158077 | G | T | cell wall protein AWA | India Wildtype |
| NC_050202 | 53022109 | 4.094506283 | T | C | cell wall protein AWA | India Wildtype |
| NC_050202 | 53779778 | 3.891733363 | T | C | uncharacterized LOC118502680 | India Wildtype |
| NC_050202 | 53779780 | 4.241989069 | A | C | uncharacterized LOC118502681 | India Wildtype |
| NC_050202 | 54216175 | 4.177137236 | G | C | F-box/LRR-repeat protein 7 | India Wildtype |
| NC_050202 | 54216241 | 4.373459555 | A | T | F-box/LRR-repeat protein 8 | India Wildtype |
| NC_050202 | 54216793 | 4.289297893 | C | G | F-box/LRR-repeat protein 9 | India Wildtype |
| NC_050202 | 54217056 | 4.386844233 | A | T | F-box/LRR-repeat protein 10 | India Wildtype |
| NC_050202 | 54429561 | 4.047323582 | T | A | histone-lysine N-methyltransferase, H3 lysine-79 specific-like | India Wildtype |
| NC_050202 | 56461081 | 4.044115365 | A | C | uncharacterized LOC118502423 | India Wildtype |
| NC_050202 | 57076494 | 4.141853548 | G | A | calcium uptake protein 3, mitochondrial | India Wildtype |
| NC_050202 | 57906894 | 3.927716691 | A | G | GATA-binding factor C | Ethiopia |
| NC_050202 | 58183563 | 4.113451924 | C | T | neural cell adhesion molecule 1-like | India Wildtype |
| NC_050202 | 58494105 | 4.022627995 | A | G | GPALPP motifs-containing protein 1 | India Wildtype |
| NC_050202 | 58508161 | 3.933334513 | T | A | cyclin-C | India Wildtype |
| NC_050202 | 58589596 | 3.914922695 | C | T | protein TANC2 | India Wildtype |
| NC_050202 | 58918129 | 4.089942954 | A | G | cardioacceleratory peptide receptor-like | India Wildtype |
| NC_050202 | 59047584 | -4.286574506 | A | T | ELMO domain-containing protein 2 | India Colony |
| NC_050202 | 60235734 | 4.119725813 | T | A | ELMO domain-containing protein 2 | Ethiopia |
| NC_050202 | 60638235 | -3.992550723 | C | T | atrophin-1-like | India Colony |
| NC_050202 | 60952754 | 4.100106655 | A | G | fibroblast growth factor receptor-like | Ethiopia |
| NC_050202 | 60991930 | 4.636823433 | T | C | fibroblast growth factor receptor-like | India Wildtype |
| NC_050202 | 61047156 | 3.939104717 | A | T | T-box transcription factor TBX2 | India Wildtype |
| NC_050202 | 61047165 | 4.180412338 | A | G | T-box transcription factor TBX3 | India Wildtype |
| NC_050202 | 61047228 | 4.318218962 | G | C | T-box transcription factor TBX4 | India Wildtype |
| NC_050202 | 61048600 | 3.973265757 | C | A | T-box transcription factor TBX1 | Ethiopia |
| NC_050202 | 61056302 | 4.243065703 | G | A | T-box transcription factor TBX5 | India Wildtype |
| NC_050202 | 61116954 | 4.004521732 | A | G | uncharacterized LOC118506545 | India Wildtype |
| NC_050202 | 61161934 | -4.050947857 | T | G | dynein light chain 1, cytoplasmic | India Wildtype |
| NC_050202 | 61169468 | 4.669028954 | G | T | dynein light chain 1, cytoplasmic | India Wildtype |
| NC_050202 | 61190821 | 4.091778742 | T | G | uncharacterized LOC118506553 | India Wildtype |
| NC_050202 | 61239856 | 4.284370879 | A | G | N-acetylglucosaminyl-phosphatidylinositol de-N-acetylase | India Wildtype |
| NC_050202 | 61276597 | -4.379225049 | C | T | suppressor of fused homolog | India Colony |
| NC_050202 | 61294066 | -4.009879631 | C | T | poly [ADP-ribose] polymerase tankyrase | India Wildtype |
| NC_050202 | 61294357 | 4.073603965 | T | A | poly [ADP-ribose] polymerase tankyrase | India Wildtype |
| NC_050202 | 61295965 | 3.910504577 | T | G | poly [ADP-ribose] polymerase tankyrase | India Wildtype |
| NC_050202 | 61296068 | 4.260527519 | G | T | poly [ADP-ribose] polymerase tankyrase | India Wildtype |
| NC_050202 | 61296173 | -4.200509067 | C | T | poly [ADP-ribose] polymerase tankyrase | India Wildtype |
| NC_050202 | 61296194 | -4.192920205 | G | T | poly [ADP-ribose] polymerase tankyrase | India Wildtype |
| NC_050202 | 61296197 | 4.455620517 | T | C | poly [ADP-ribose] polymerase tankyrase | India Wildtype |
| NC_050202 | 61296198 | 4.567227031 | A | C | poly [ADP-ribose] polymerase tankyrase | India Wildtype |
| NC_050202 | 61296201 | 4.567227031 | C | T | poly [ADP-ribose] polymerase tankyrase | India Wildtype |
| NC_050202 | 61324363 | 4.009526361 | G | C | beta-TrCP | India Wildtype |
| NC_050202 | 61328023 | -4.177031037 | G | C | beta-TrCP | India Wildtype |
| NC_050202 | 61328315 | 4.122107035 | T | G | beta-TrCP | India Wildtype |
| NC_050202 | 61328347 | 4.131527884 | C | G | beta-TrCP | India Wildtype |
| NC_050202 | 61328354 | -3.920167419 | C | A | beta-TrCP | India Wildtype |
| NC_050202 | 61366766 | 4.086546791 | G | A | 39S ribosomal protein L33, mitochondrial | India Wildtype |
| NC_050202 | 61366784 | -4.155793744 | G | T | 39S ribosomal protein L33, mitochondrial | India Wildtype |
| NC_050202 | 61366786 | 4.598049444 | G | T | 39S ribosomal protein L33, mitochondrial | India Wildtype |
| NC_050202 | 61378217 | 4.042919297 | C | T | 1-phosphatidylinositol 4,5-bisphosphate phosphodiesterase | India Wildtype |
| NC_050202 | 61434500 | -3.895314466 | G | A | uncharacterized protein CG43867 | India Wildtype |
| NC_050202 | 61508479 | 3.894579549 | C | T | uncharacterized protein CG43867 | India Wildtype |
| NC_050202 | 61525001 | 4.359346377 | G | A | uncharacterized protein CG43867 | India Wildtype |
| NC_050202 | 61537579 | -3.989995025 | G | T | uncharacterized protein CG43867 | India Wildtype |
| NC_050202 | 61562536 | 4.185822086 | T | C | uncharacterized protein CG43867 | India Wildtype |
| NC_050202 | 61782661 | 4.130774225 | G | A | nephrin-like | India Wildtype |
| NC_050202 | 62041935 | 3.995115017 | T | C | odorant receptor 85c-like | India Wildtype |
| NC_050202 | 62043925 | 3.999026741 | C | T | odorant receptor 85c-like | Ethiopia |
| NC_050202 | 62182447 | 4.029376189 | C | T | protein elav | India Wildtype |
| NC_050202 | 62192176 | 4.101782379 | T | C | 40S ribosomal protein S12, mitochondrial | India Wildtype |
| NC_050202 | 62315314 | 4.476391896 | G | C | b(0,+)-type amino acid transporter | India Wildtype |
| NC_050202 | 62319575 | -3.96395661 | T | C | b(0,+)-type amino acid transporter | India Colony |
| NC_050202 | 62343395 | 3.957110759 | A | G | dual specificity mitogen-activated protein kinase kinase hemipterous-like | India Wildtype |
| NC_050202 | 62391746 | -4.619754688 | A | G | uncharacterized LOC118506651 | India Wildtype |
| NC_050202 | 62396796 | 4.064030358 | T | A | uncharacterized LOC118506652 | India Wildtype |
| NC_050202 | 62396799 | 4.064030358 | C | T | uncharacterized LOC118506653 | India Wildtype |
| NC_050202 | 62459584 | -3.918080786 | C | A | FMRFamide receptor-like | India Wildtype |
| NC_050202 | 62478020 | -3.913706458 | A | G | FMRFamide receptor-like | India Wildtype |
| NC_050202 | 62478023 | -4.084936989 | A | T | FMRFamide receptor-like | India Wildtype |
| NC_050202 | 62478518 | 3.987296425 | G | A | FMRFamide receptor-like | India Wildtype |
| NC_050202 | 62489482 | 3.95694118 | A | C | FMRFamide receptor-like | India Wildtype |
| NC_050202 | 62516802 | -4.151921039 | C | T | FMRFamide receptor-like | India Wildtype |
| NC_050202 | 62516805 | -4.151921039 | A | T | FMRFamide receptor-like | India Wildtype |
| NC_050202 | 62545839 | -4.238339178 | A | G | ATP-binding cassette sub-family G member 1-like | India Wildtype |
| NC_050202 | 62692366 | -4.268848023 | G | A | uncharacterized LOC118506670 | India Wildtype |
| NC_050202 | 62693004 | -5.063278451 | G | A | uncharacterized LOC118506670 | India Wildtype |
| NC_050202 | 62693969 | -3.935088476 | C | T | uncharacterized LOC118506670 | India Wildtype |
| NC_050202 | 62696202 | -4.164073127 | A | T | uncharacterized LOC118506670 | India Wildtype |
| NC_050202 | 62696214 | 4.444366399 | A | G | uncharacterized LOC118506670 | India Wildtype |
| NC_050202 | 62696228 | 4.308830863 | G | A | uncharacterized LOC118506670 | India Wildtype |
| NC_050202 | 62831643 | -4.033421862 | T | A | ankyrin-3 | India Wildtype |
| NC_050202 | 62857378 | 4.098597299 | C | A | ankyrin-3 | India Wildtype |
| NC_050202 | 62871220 | 4.62082764 | T | A | ankyrin-3 | India Wildtype |
| NC_050202 | 62871561 | 3.904217071 | A | C | ankyrin-3 | India Wildtype |
| NC_050202 | 63632827 | 4.310398399 | T | C | uncharacterized LOC118506742 | India Wildtype |
| NC_050202 | 63647692 | -3.914714806 | A | T | myoneurin-like | India Wildtype |
| NC_050202 | 63914652 | 4.267358399 | G | T | extracellular sulfatase SULF-1 homolog | India Wildtype |
| NC_050202 | 64541869 | -4.139641914 | A | T | organic cation transporter protein | India Wildtype |
| NC_050202 | 64757546 | 4.049568944 | G | A | nose resistant to fluoxetine protein 6 | India Wildtype |
| NC_050202 | 65258307 | 4.349756526 | G | C | tubulin-specific chaperone A | India Wildtype |
| NC_050202 | 65261825 | 4.479194698 | G | T | BRCA1-associated protein | India Wildtype |
| NC_050202 | 65273072 | 4.021366747 | A | T | odorant receptor 94a-like | India Wildtype |
| NC_050202 | 65273615 | 4.200722517 | T | A | odorant receptor 94a-like | India Wildtype |
| NC_050202 | 65285629 | 4.191217549 | T | A | axin | India Wildtype |
| NC_050202 | 65286386 | 4.205374839 | G | C | axin | India Wildtype |
| NC_050202 | 65287196 | 4.238893925 | A | G | axin | India Wildtype |
| NC_050202 | 66875019 | -4.203420953 | G | C | ncharacterized LOC118504356 | India Colony |
| NC_050202 | 66875027 | -4.203420953 | C | G | ncharacterized LOC118504357 | India Colony |
| NC_050202 | 66999978 | 3.959845501 | C | T | uncharacterized LOC118504363 | Ethiopia |
| NC_050202 | 67084282 | -4.11239482 | C | T | cadherin-23-like | India Colony |
| NC_050202 | 67269104 | -4.513743563 | T | A | uncharacterized LOC118504384 | India Colony |
| NC_050202 | 67274986 | -3.91500133 | A | T | zinc finger and BTB domain-containing protein 17-like | India Colony |
| NC_050202 | 67275023 | -4.099502609 | A | T | zinc finger and BTB domain-containing protein 17-like | India Colony |
| NC_050202 | 67407539 | 4.101100733 | G | A | sodium/calcium exchanger 3 | Ethiopia |
| NC_050202 | 67818447 | -4.121155184 | C | T | octopamine receptor beta-3R-like | India Colony |
| NC_050202 | 68529960 | -4.139439583 | A | G | uncharacterized LOC118504479 | India Wildtype |
| NC_050202 | 68650001 | 3.973230284 | T | C | mucin-5AC-like | India Wildtype |
| NC_050202 | 68736715 | 4.163205917 | T | C | acetylcholine receptor subunit beta-like 2 | India Wildtype |
| NC_050202 | 68736717 | 4.244479155 | C | A | acetylcholine receptor subunit beta-like 3 | India Wildtype |
| NC_050202 | 68789775 | -4.533588827 | G | A | acetylcholine receptor subunit alpha-like 2 | India Wildtype |
| NC_050202 | 68790027 | -4.205614083 | T | C | acetylcholine receptor subunit alpha-like 2 | India Wildtype |
| NC_050202 | 68790037 | -4.525452283 | G | T | acetylcholine receptor subunit alpha-like 2 | India Wildtype |
| NC_050202 | 68790043 | -4.511803665 | A | T | acetylcholine receptor subunit alpha-like 2 | India Wildtype |
| NC_050202 | 68874338 | 3.97023238 | C | T | acetylcholine receptor subunit alpha-like | Ethiopia |
| NC_050202 | 69480790 | -4.150272452 | C | T | broad-complex core protein isoforms 1/2/3/4/5 | India Wildtype |
| NC_050202 | 69732934 | 4.199955383 | C | A | homeobox protein homothorax | India Wildtype |
| NC_050202 | 70010058 | 4.158893889 | G | A | low-density lipoprotein receptor | India Wildtype |
| NC_050202 | 70047918 | 4.425811381 | A | G | low-density lipoprotein receptor | India Wildtype |
| NC_050202 | 70311936 | -3.922492078 | T | G | uncharacterized LOC118504591 | India Wildtype |
| NC_050202 | 72304941 | 3.928831317 | T | C | elongation of very long chain fatty acids protein | India Wildtype |
| NC_050202 | 72306517 | 4.304559602 | T | C | elongation of very long chain fatty acids protein | India Wildtype |
| NC_050202 | 72346827 | 4.035755408 | T | C | 60S acidic ribosomal protein P0 | India Wildtype |
| NC_050202 | 72699026 | 4.037317517 | G | A | protein eva-1-like | India Wildtype |
| NC_050202 | 73460397 | 4.377269194 | T | A | protein tramtrack, beta isoform | India Wildtype |
| NC_050202 | 73460398 | 4.377269194 | G | A | protein tramtrack, beta isoform | India Wildtype |
| NC_050202 | 73460401 | 4.462191698 | C | T | protein tramtrack, beta isoform | India Wildtype |
| NC_050202 | 73460412 | 3.907056704 | C | T | protein tramtrack, beta isoform | India Wildtype |
| NC_050202 | 73463637 | 4.303908898 | A | G | protein tramtrack, beta isoform | India Wildtype |
| NC_050202 | 73475342 | 4.194092969 | G | T | protein tramtrack | India Wildtype |
| NC_050202 | 73477885 | 4.4947539 | A | T | protein tramtrack, beta isoform | India Wildtype |
| NC_050202 | 73480627 | 3.99668325 | T | A | S-adenosylmethionine mitochondrial carrier protein homolo | India Wildtype |
| NC_050202 | 73480659 | 4.230182138 | A | C | S-adenosylmethionine mitochondrial carrier protein homolo | India Wildtype |
| NC_050202 | 73480690 | 4.238026822 | C | T | S-adenosylmethionine mitochondrial carrier protein homolo | India Wildtype |
| NC_050202 | 73480693 | 4.582333218 | T | G | S-adenosylmethionine mitochondrial carrier protein homolo | India Wildtype |
| NC_050202 | 73483399 | -3.952290934 | G | A | uncharacterized LOC118504260 | India Wildtype |
| NC_050202 | 73483976 | 3.960225746 | A | C | single-pass membrane and coiled-coil domain-containing protein 4 homolo | India Wildtype |
| NC_050202 | 73484172 | 4.637969226 | G | T | single-pass membrane and coiled-coil domain-containing protein 4 homolo | India Wildtype |
| NC_050202 | 73484193 | 3.926458152 | C | T | single-pass membrane and coiled-coil domain-containing protein 4 homolo | India Wildtype |
| NC_050202 | 73484909 | 3.916353417 | C | T | mitochondrial import inner membrane translocase subunit TIM14 | India Wildtype |
| NC_050202 | 73484930 | 4.314427296 | T | A | mitochondrial import inner membrane translocase subunit TIM15 | India Wildtype |
| NC_050202 | 73488129 | 4.213253472 | T | C | uncharacterized LOC118504111 | India Wildtype |
| NC_050202 | 73491551 | 4.033370223 | G | A | uncharacterized LOC118504263 | India Wildtype |
| NC_050202 | 73696689 | 4.048260168 | T | C | complexin | India Wildtype |
| NC_050202 | 74164291 | -4.2196074 | C | T | uncharacterized LOC118505829 | India Colony |
| NC_050202 | 74208464 | 4.022106301 | T | A | uncharacterized LOC118505831 | India Wildtype |
| NC_050202 | 74209057 | -3.912692262 | A | T | uncharacterized LOC118505832 | India Wildtype |
| NC_050202 | 74217706 | -3.909400197 | C | T | uncharacterized LOC118505830 | India Colony |
| NC_050202 | 74520216 | 4.255036379 | T | G | rootletin | India Wildtype |
| NC_050202 | 75028020 | -4.239456804 | G | T | fatty acid hydroxylase domain-containing protein 2-lik | India Wildtype |
| NC_050202 | 75028131 | -3.894735479 | A | C | fatty acid hydroxylase domain-containing protein 2-lik | India Wildtype |
| NC_050202 | 75127185 | 4.082354518 | A | C | TLD domain-containing protein 2 | India Wildtype |
| NC_050202 | 75430232 | -4.051909749 | T | G | protein tweety-2-like | India Wildtype |
| NC_050202 | 76556452 | 3.931566891 | T | C | uncharacterized LOC118507728 | Ethiopia |
| NC_050202 | 76570256 | 4.158374082 | A | C | uncharacterized LOC118507728 | India Wildtype |
| NC_050202 | 76661283 | 3.973739084 | C | T | relaxin receptor 2 | India Wildtype |
| NC_050202 | 76758451 | -3.926331222 | A | C | hemicentin-1 | India Wildtype |
| NC_050202 | 76811059 | -3.979860678 | A | G | hemicentin-1 | India Wildtype |
| NC_050202 | 76820348 | -3.954433119 | C | T | hemicentin-1 | India Wildtype |
| NC_050202 | 78336763 | 3.938106262 | T | A | uncharacterized LOC118508495 | India Wildtype |
| NC_050202 | 78662880 | 4.067194196 | C | A | protein FAM160B1-like | Ethiopia |
| NC_050202 | 78746627 | 3.921886091 | G | T | putative transcription factor capicua | India Wildtype |
| NC_050202 | 78849305 | 4.609899069 | C | T | neuroligin-3 | India Wildtype |
| NC_050202 | 78849308 | 4.406001097 | C | T | neuroligin-4 | India Wildtype |
| NC_050202 | 78957030 | 4.041904096 | C | T | serine/threonine-protein phosphatase PP1-beta catalytic subunit | India Wildtype |
| NC_050202 | 78957400 | 4.119881504 | A | T | serine/threonine-protein phosphatase PP1-beta catalytic subunit | India Wildtype |
| NC_050202 | 78962993 | 4.048399393 | G | T | serine/threonine-protein phosphatase PP1-beta catalytic subunit | India Wildtype |
| NC_050202 | 78963182 | 4.324504937 | G | T | serine/threonine-protein phosphatase PP1-beta catalytic subunit | India Wildtype |
| NC_050202 | 78963185 | 4.576009348 | C | A | serine/threonine-protein phosphatase PP1-beta catalytic subunit | India Wildtype |
| NC_050202 | 78965248 | 4.273713615 | G | T | serine/threonine-protein phosphatase PP1-beta catalytic subunit | India Wildtype |
| NC_050202 | 78965351 | 3.918888488 | C | A | serine/threonine-protein phosphatase PP1-beta catalytic subunit | India Wildtype |
| NC_050202 | 78965402 | 4.343813516 | A | T | serine/threonine-protein phosphatase PP1-beta catalytic subunit | India Wildtype |
| NC_050202 | 78967333 | 4.173951734 | T | A | serine/threonine-protein phosphatase PP1-beta catalytic subunit | India Wildtype |
| NC_050202 | 78977684 | 4.019040148 | T | G | fibroblast growth factor receptor homolog 1-like | India Wildtype |
| NC_050202 | 78979224 | 3.934280685 | A | T | fibroblast growth factor receptor homolog 1-like | India Wildtype |
| NC_050202 | 78979231 | 4.307698189 | T | A | fibroblast growth factor receptor homolog 1-like | India Wildtype |
| NC_050202 | 79098385 | -3.96942058 | G | A | uncharacterized LOC118505424 | India Wildtype |
| NC_050202 | 79101675 | 4.69057439 | A | G | uncharacterized LOC118505425 | India Wildtype |
| NC_050202 | 79102038 | 4.135748973 | G | T | uncharacterized LOC118505426 | India Wildtype |
| NC_050202 | 79102052 | 4.143033367 | G | A | uncharacterized LOC118505427 | India Wildtype |
| NC_050202 | 79125852 | 4.286388161 | C | G | uncharacterized LOC118505424 | India Wildtype |
| NC_050202 | 79933862 | 4.343552122 | G | A | soluble guanylate cyclase 88E | India Wildtype |
| NC_050202 | 80295326 | 4.159447328 | C | T | connectin-like | India Wildtype |
| NC_050202 | 80302297 | 4.190157554 | A | T | connectin-like | India Wildtype |
| NC_050202 | 80340317 | -4.249673152 | T | C | connectin-like | India Wildtype |
| NC_050202 | 80340339 | -4.1204357 | C | A | connectin-like | India Wildtype |
| NC_050202 | 80356943 | 4.020078734 | G | A | connectin-like | India Wildtype |
| NC_050202 | 80359828 | 3.915350696 | C | A | connectin-like | India Wildtype |
| NC_050202 | 80359908 | 4.11710405 | T | C | connectin-like | India Wildtype |
| NC_050202 | 80395992 | 4.126087428 | G | A | connectin-like | India Wildtype |
| NC_050202 | 80440102 | 3.952149368 | T | A | connectin-like | India Wildtype |
| NC_050202 | 80440486 | -3.964274194 | C | T | connectin-like | India Wildtype |
| NC_050202 | 80444851 | -4.030386017 | G | A | connectin-like | India Wildtype |
| NC_050202 | 84259372 | 4.421996208 | G | T | uncharacterized LOC118506186 | India Wildtype |
| NC_050202 | 87297780 | 4.84690973 | T | G | MAGUK p55 subfamily member 5-A | Pakistan Colony |
| NC_050202 | 87707388 | 3.897748473 | G | A | histone-lysine N-methyltransferase trithorax | India Wildtype |
| NC_050202 | 91353436 | 4.118492968 | G | C | neuropeptide CCHamide-2 receptor-like | India Wildtype |
| NC_050203 | 478510 | 3.937395906 | C | T | mucin-5AC | Ethiopia |
| NC_050203 | 2064822 | 3.915041557 | G | T | oronin-1C-like | Ethiopia |
| NC_050203 | 2166509 | 3.893462167 | T | A | phosphatidylinositol 4-kinase beta | India Wildtype |
| NC_050203 | 2255256 | 4.083349291 | G | A | pneumococcal serine-rich repeat protein | Ethiopia |
| NC_050203 | 2257529 | 4.212292305 | A | G | pneumococcal serine-rich repeat protein | Ethiopia |
| NC_050203 | 3259152 | -4.032792425 | G | A | glucose transporter type 1 | Ethiopia |
| NC_050203 | 3983354 | -3.903111758 | A | G | ribonuclease P protein subunit p25-like protein | India Colony |
| NC_050203 | 3983355 | -3.903111758 | C | T | ribonuclease P protein subunit p25-like protein | India Colony |
| NC_050203 | 5689668 | 3.971559414 | G | A | potassium voltage-gated channel protein Shab | India Wildtype |
| NC_050203 | 6288011 | -4.020059827 | A | C | uncharacterized LOC118510401 | India Colony |
| NC_050203 | 6412679 | -3.942697834 | C | T | uncharacterized LOC118510402 | India Colony |
| NC_050203 | 6412688 | -3.969803744 | T | C | uncharacterized LOC118510402 | India Colony |
| NC_050203 | 6412692 | -4.17792554 | G | A | uncharacterized LOC118510402 | India Colony |
| NC_050203 | 6412839 | -4.023359591 | T | A | uncharacterized LOC118510402 | India Colony |
| NC_050203 | 7832887 | -4.012648837 | G | T | mediator of RNA polymerase II transcription subunit 26 | India Wildtype |
| NC_050203 | 7854743 | -4.158523972 | A | C | uncharacterized LOC118510494 | India Wildtype |
| NC_050203 | 7854755 | -4.132592368 | A | T | uncharacterized LOC118510494 | India Wildtype |
| NC_050203 | 8204081 | -3.914415377 | C | A | transcriptional coactivator YAP1-A-like | India Colony |
| NC_050203 | 8227542 | -4.011421343 | A | G | WD repeat-containing protein 26 homolog | India Colony |
| NC_050203 | 8347167 | 4.262880468 | A | C | gamma-aminobutyric acid receptor subunit beta | India Wildtype |
| NC_050203 | 8441087 | 4.253055482 | A | G | glutamate receptor 1-like | India Wildtype |
| NC_050203 | 8595092 | 4.090429303 | A | C | glutamate receptor 1-like | India Wildtype |
| NC_050203 | 8703703 | 3.986269939 | A | G | uncharacterized LOC118514464 | India Wildtype |
| NC_050203 | 8877427 | -3.986743238 | T | G | protein bric-a-brac 1-like | India Wildtype |
| NC_050203 | 8879557 | -4.247636633 | C | T | protein bric-a-brac 1-like | India Wildtype |
| NC_050203 | 8880729 | 4.493191596 | T | C | protein bric-a-brac 1-like | India Wildtype |
| NC_050203 | 8881800 | 4.567791359 | G | C | protein bric-a-brac 1-like | India Wildtype |
| NC_050203 | 8884631 | 3.984795407 | A | C | protein bric-a-brac 1-like | India Wildtype |
| NC_050203 | 8887404 | 3.902689977 | C | G | protein bric-a-brac 1-like | India Wildtype |
| NC_050203 | 8887496 | 4.034224977 | C | A | protein bric-a-brac 1-like | India Wildtype |
| NC_050203 | 8888620 | 4.168039608 | A | G | protein bric-a-brac 1-like | India Wildtype |
| NC_050203 | 8889063 | 4.650291184 | T | A | protein bric-a-brac 1-like | India Wildtype |
| NC_050203 | 8889065 | 4.963989489 | C | G | protein bric-a-brac 1-like | India Wildtype |
| NC_050203 | 8891070 | 4.087013355 | A | G | protein bric-a-brac 1-like | India Wildtype |
| NC_050203 | 8891093 | 4.344924629 | C | T | protein bric-a-brac 1-like | India Wildtype |
| NC_050203 | 8891185 | 4.068306794 | T | C | protein bric-a-brac 1-like | India Wildtype |
| NC_050203 | 8891194 | 4.068306794 | G | A | protein bric-a-brac 1-like | India Wildtype |
| NC_050203 | 8891203 | -3.988242501 | G | A | protein bric-a-brac 1-like | India Wildtype |
| NC_050203 | 8891219 | -4.205879164 | T | A | protein bric-a-brac 1-like | India Wildtype |
| NC_050203 | 8891242 | -3.906438921 | A | T | protein bric-a-brac 1-like | India Wildtype |
| NC_050203 | 8891300 | 4.067389679 | G | T | protein bric-a-brac 1-like | India Wildtype |
| NC_050203 | 8891418 | -3.968003737 | T | C | protein bric-a-brac 1-like | India Wildtype |
| NC_050203 | 8891420 | -3.968003737 | C | T | protein bric-a-brac 1-like | India Wildtype |
| NC_050203 | 8893294 | -4.303004207 | G | A | protein bric-a-brac 1-like | India Wildtype |
| NC_050203 | 8893296 | -4.303004207 | T | C | protein bric-a-brac 1-like | India Wildtype |
| NC_050203 | 8893370 | -4.187102265 | C | T | protein bric-a-brac 1-like | India Wildtype |
| NC_050203 | 8893386 | -4.392207544 | A | C | protein bric-a-brac 1-like | India Wildtype |
| NC_050203 | 8894173 | 4.44927355 | G | C | protein bric-a-brac 1-like | India Wildtype |
| NC_050203 | 8894179 | 4.44927355 | T | C | protein bric-a-brac 1-like | India Wildtype |
| NC_050203 | 8895458 | -4.082225506 | C | T | protein bric-a-brac 1-like | India Wildtype |
| NC_050203 | 8895662 | 4.089774238 | G | A | protein bric-a-brac 1-like | India Wildtype |
| NC_050203 | 8895663 | 4.089774238 | G | A | protein bric-a-brac 1-like | India Wildtype |
| NC_050203 | 8897477 | 4.089388769 | A | C | protein bric-a-brac 1-like | India Wildtype |
| NC_050203 | 8897488 | 4.068826679 | G | A | protein bric-a-brac 1-like | India Wildtype |
| NC_050203 | 8897515 | -4.220786627 | A | T | protein bric-a-brac 1-like | India Wildtype |
| NC_050203 | 8897540 | -3.941156127 | T | C | protein bric-a-brac 1-like | India Wildtype |
| NC_050203 | 8917473 | 3.927241723 | A | G | protein bric-a-brac 1-like | India Wildtype |
| NC_050203 | 8920043 | 3.99467293 | C | A | protein bric-a-brac 1-like | India Wildtype |
| NC_050203 | 8922858 | -4.018184566 | G | T | protein bric-a-brac 1-like | India Wildtype |
| NC_050203 | 8925835 | 3.962858477 | C | T | protein bric-a-brac 1-like | India Wildtype |
| NC_050203 | 8948642 | -4.292883966 | C | T | protein bric-a-brac 1-like | India Wildtype |
| NC_050203 | 8959562 | 3.924573552 | C | T | protein bric-a-brac 1-like | India Wildtype |
| NC_050203 | 11047718 | -3.916370586 | C | T | uncharacterized LOC118513184 | Ethiopia |
| NC_050203 | 15208656 | 3.986273909 | G | C | sphingomyelinase-like phosphodiesterase 3a | India Wildtype |
| NC_050203 | 15606721 | 4.012410716 | G | A | platelet binding protein GspB | Pakistan Colony |
| NC_050203 | 15715262 | 3.948210664 | T | C | collagen alpha-1(XVIII) chain | Ethiopia |
| NC_050203 | 15797246 | -3.997700249 | C | T | collagen alpha-1(XVIII) chain | India Colony |
| NC_050203 | 16805773 | 3.986061397 | C | T | uncharacterized LOC118512690 | India Wildtype |
| NC_050203 | 16807970 | 4.460931762 | T | C | uncharacterized LOC118512690 | India Wildtype |
| NC_050203 | 17618039 | 4.048949994 | T | G | mediator of RNA polymerase II transcription subunit 13-like | India Wildtype |
| NC_050203 | 20277394 | 4.262857428 | G | A | cold shock domain-containing protein CG9705-like | India Wildtype |
| NC_050203 | 20480633 | -4.265950873 | G | A | NADH dehydrogenase [ubiquinone] 1 alpha subcomplex subunit 5 | India Colony |
| NC_050203 | 20668384 | 3.94395547 | A | T | glutamate-gated chloride channel | Pakistan Colony |
| NC_050203 | 20668385 | 3.94395547 | A | T | glutamate-gated chloride channel | Pakistan Colony |
| NC_050203 | 21836784 | 3.991798463 | T | C | putative mediator of RNA polymerase II transcription subunit 26 | India Wildtype |
| NC_050203 | 21836797 | 4.231347269 | T | C | putative mediator of RNA polymerase II transcription subunit 26 | India Wildtype |
| NC_050203 | 21836803 | 3.905148853 | T | C | putative mediator of RNA polymerase II transcription subunit 26 | India Wildtype |
| NC_050203 | 21836807 | 4.394234004 | A | T | putative mediator of RNA polymerase II transcription subunit 26 | India Wildtype |
| NC_050203 | 22031200 | -4.029521128 | T | A | farnesol dehydrogenase-like | Pakistan Colony |
| NC_050203 | 22057256 | 3.945302639 | G | A | protein boule | India Wildtype |
| NC_050203 | 22738069 | 3.99011119 | A | G | uncharacterized LOC118510558 | Pakistan Colony |
| NC_050203 | 22740581 | 4.389195552 | G | C | uncharacterized LOC118510558 | Pakistan Colony |
| NC_050203 | 22741081 | 4.137371693 | C | A | uncharacterized LOC118510558 | Pakistan Colony |
| NC_050203 | 23486310 | 3.904106162 | C | T | uncharacterized LOC118511319 | India Wildtype |
| NC_050203 | 23592673 | 4.517310182 | T | A | uncharacterized LOC118511319 | India Wildtype |
| NC_050203 | 24270892 | 3.895200947 | T | G | uncharacterized LOC118511351 | India Wildtype |
| NC_050203 | 24270894 | 3.895234988 | G | A | uncharacterized LOC118511351 | India Wildtype |
| NC_050203 | 24275779 | 4.328063018 | T | A | uncharacterized LOC118511351 | India Wildtype |
| NC_050203 | 24418571 | 3.964584487 | G | A | uncharacterized LOC118511351 | India Wildtype |
| NC_050203 | 24532458 | 3.978921586 | C | G | 5-hydroxytryptamine receptor-like | India Wildtype |
| NC_050203 | 24937241 | -4.112391516 | G | A | uncharacterized LOC118511385 | India Wildtype |
| NC_050203 | 25887792 | 3.939366369 | A | T | peptidyl-alpha-hydroxyglycine alpha-amidating lyase 2 | India Wildtype |
| NC_050203 | 26235010 | 4.049734971 | T | A | feline leukemia virus subgroup C receptor-related protein 2 | Ethiopia |
| NC_050203 | 27616117 | 4.03266111 | A | G | protein dead ringer-like | India Wildtype |
| NC_050203 | 29140193 | -3.900004089 | T | G | SCY1-like protein 2 | India Wildtype |
| NC_050203 | 30144976 | 4.403905379 | G | A | four and a half LIM domains protein 2 | India Wildtype |
| NC_050203 | 31659869 | 4.109572841 | A | G | serine/threonine-protein kinase mig-15 | India Wildtype |
| NC_050203 | 31659950 | -3.914534635 | A | G | serine/threonine-protein kinase mig-15 | India Wildtype |
| NC_050203 | 31676858 | 3.903696528 | T | G | serine/threonine-protein kinase mig-15 | India Wildtype |
| NC_050203 | 31997767 | -3.895882219 | C | G | uncharacterized LOC118509520 | India Wildtype |
| NC_050203 | 32006421 | 4.394024703 | C | A | uncharacterized LOC118509520 | Pakistan Colony |
| NC_050203 | 32006422 | 4.394024703 | A | G | uncharacterized LOC118509520 | Pakistan Colony |
| NC_050203 | 32303949 | 3.971976239 | G | A | thyrotroph embryonic factor | India Wildtype |
| NC_050203 | 32439278 | 4.050326433 | G | A | uncharacterized LOC118509543 | India Wildtype |
| NC_050203 | 33243478 | 4.293820336 | T | C | uncharacterized LOC118509574 | India Wildtype |
| NC_050203 | 33248109 | -3.990493375 | T | C | uncharacterized LOC118509574 | India Wildtype |
| NC_050203 | 33346260 | 4.00529455 | C | A | multidrug resistance-associated protein lethal(2)03659 | India Wildtype |
| NC_050203 | 33459620 | 3.909133951 | G | T | mediator of RNA polymerase II transcription subunit 13 | India Wildtype |
| NC_050203 | 35326012 | 3.950493515 | C | G | myc box-dependent-interacting protein 1 | India Wildtype |
| NC_050203 | 39034801 | 3.96668872 | G | T | Down syndrome cell adhesion molecule-like protein Dscam2 | India Wildtype |
| NC_050203 | 40120407 | 4.078892075 | T | C | myb-like protein Q ( | India Wildtype |
| NC_050203 | 40461670 | 3.913176546 | C | T | potassium voltage-gated channel subfamily H member 8 | India Wildtype |
| NC_050203 | 40718842 | -4.092562353 | T | G | G-protein coupled receptor daf-37 | India Wildtype |
| NC_050203 | 41609080 | 4.425766911 | T | A | protein furry | Pakistan Colony |
| NC_050203 | 42470687 | 3.970429688 | T | C | uncharacterized LOC118512388 | India Wildtype |
| NC_050203 | 42626612 | 3.969560281 | A | T | group 3 secretory phospholipase A2 | Ethiopia |
| NC_050203 | 42629395 | 4.260116186 | G | T | group 3 secretory phospholipase A2 | Ethiopia |
| NC_050203 | 42629396 | 4.260116186 | T | A | group 3 secretory phospholipase A2 | Ethiopia |
| NC_050203 | 44429165 | 3.933920755 | A | C | uncharacterized LOC118511032 | India Wildtype |
| NC_050203 | 44630205 | 3.929612093 | A | T | lachesin-like | Pakistan Colony |
| NC_050203 | 45685454 | 4.113065836 | C | T | carnitine O-palmitoyltransferase 1, liver isoform | India Wildtype |
| NC_050203 | 46874908 | 4.24130142 | G | T | carbonic anhydrase 2 | India Wildtype |
| NC_050203 | 47670654 | -4.200443321 | A | C | zinc finger protein 773 | India Colony |
| NC_050203 | 47670662 | -4.081855259 | C | A | zinc finger protein 773 | India Colony |
| NC_050203 | 48000964 | 3.921918883 | C | T | dual oxidase maturation factor 1 | Ethiopia |
| NC_050203 | 48120832 | -4.026021145 | A | G | lachesin-like | India Colony |
| NC_050203 | 48130118 | -3.929132851 | G | C | lachesin-like | India Colony |
| NC_050203 | 48133318 | -3.962128472 | T | G | lachesin-like | India Colony |
| NC_050203 | 48133337 | -3.905111958 | T | A | lachesin-like | India Colony |
| NC_050203 | 48443062 | 3.997969911 | C | T | transcription factor SOX-3-like | India Wildtype |
| NC_050203 | 48638850 | 3.900381393 | C | G | uncharacterized LOC118512654 | India Wildtype |
| NC_050203 | 48748015 | -4.041527205 | A | T | AF4/FMR2 family member lilli | India Wildtype |
| NC_050203 | 50443326 | 4.545217431 | T | C | fibrinogen alpha chain | Pakistan Colony |
| NC_050203 | 51139214 | 3.902955074 | G | T | uncharacterized LOC118514565 | India Wildtype |
| NC_050203 | 51139815 | 3.953313226 | G | A | uncharacterized LOC118514565 | India Wildtype |
| NC_050203 | 53069096 | 3.956041313 | G | T | uncharacterized LOC118509215 | Ethiopia |
| NC_050203 | 54310053 | -4.141275556 | A | G | pupal cuticle protein 36-like | India Colony |
| NC_050203 | 54320414 | -3.892651881 | G | A | endocuticle structural glycoprotein ABD-4-like | India Colony |
| NC_050203 | 54320437 | -3.932561247 | T | C | endocuticle structural glycoprotein ABD-4-like | India Colony |
| NC_050203 | 54332752 | -3.932463563 | G | A | larval cuticle protein LCP-30-like | India Colony |
| NC_050203 | 54426118 | -3.963423924 | T | A | profilin | India Colony |
| NC_050203 | 54466480 | -4.366315817 | G | C | mucin-5AC | India Colony |
| NC_050203 | 54476761 | -3.918002175 | A | T | mucin-5AC | India Colony |
| NC_050203 | 54479203 | -4.034560304 | G | C | mucin-5AC | India Colony |
| NC_050203 | 54557454 | 5.121675998 | T | C | mucin-5AC | Pakistan Colony |
| NC_050203 | 54689318 | -3.927991283 | A | G | protein outspread | India Colony |
| NC_050203 | 54692925 | -4.063340678 | A | T | protein outspread | India Colony |
| NC_050203 | 54715577 | 4.134176435 | G | A | protein outspread | Pakistan Colony |
| NC_050203 | 54812436 | -3.950822496 | C | T | protein outspread | India Colony |
| NC_050203 | 54889392 | 3.987835672 | C | T | protein outspread | India Wildtype |
| NC_050203 | 54909264 | -4.167999762 | A | G | ras-related protein Rab-9B | India Colony |
| NC_050203 | 58105799 | 4.074797354 | G | A | serine protease filzig | India Wildtype |
| NC_050203 | 58180357 | 4.072281088 | C | A | ras-related and estrogen-regulated growth inhibitor-like protein | India Wildtype |
| NC_050203 | 58188974 | 4.070571249 | A | C | ras-related and estrogen-regulated growth inhibitor-like protein | India Wildtype |
| NC_050203 | 58188975 | 4.096277383 | T | A | ras-related and estrogen-regulated growth inhibitor-like protein | India Wildtype |
| NC_050203 | 58192447 | 4.681586845 | C | T | ras-related and estrogen-regulated growth inhibitor-like protein | India Wildtype |
| NC_050203 | 58192448 | 4.100359867 | C | G | ras-related and estrogen-regulated growth inhibitor-like protein | India Wildtype |
| NC_050203 | 58285913 | 3.89289782 | G | A | cyclin-dependent serine/threonine-protein kinase DDB_G0292550 | India Wildtype |
| NC_050203 | 58289967 | 4.073518552 | G | T | cyclin-dependent serine/threonine-protein kinase DDB_G0292551 | India Wildtype |
| NC_050203 | 58644836 | 3.893213902 | T | C | calcium/calmodulin-dependent 3',5'-cyclic nucleotide phosphodiesterase 1-like | Ethiopia |
| NC_050203 | 58988253 | 3.917467603 | T | C | zinc finger protein chinmo | India Wildtype |
| NC_050203 | 59120814 | 3.981962869 | G | A | serine/threonine-protein kinase DDB_G0282963 | India Wildtype |
| NC_050203 | 63902056 | -4.59828046 | A | G | robable serine/threonine-protein kinase clkA | India Colony |
| NC_050203 | 64345674 | 4.343593247 | T | C | kelch-like protein 17 | India Wildtype |
| NC_050203 | 66401320 | 4.141994342 | T | A | discoidin domain-containing receptor tyrosine kinase B | India Wildtype |
| NC_050203 | 67922600 | 4.092162604 | G | A | cytochrome P450 307a1-like | India Wildtype |
| NC_050203 | 67925612 | 3.917983433 | T | C | cytochrome P450 307a1-like | India Wildtype |
| NC_050203 | 67925620 | 3.925032323 | T | A | cytochrome P450 307a1-like | India Wildtype |
| NC_050203 | 67925893 | 4.308935301 | G | T | cytochrome P450 307a1-like | India Wildtype |
| NC_050203 | 67926103 | 4.251372684 | C | A | cytochrome P450 307a1-like | India Wildtype |
| NC_050203 | 67926118 | 4.033621438 | A | C | cytochrome P450 307a1-like | India Wildtype |
| NC_050203 | 67926235 | 4.11747932 | C | T | cytochrome P450 307a1-like | India Wildtype |
| NC_050203 | 68255443 | 4.574665565 | G | C | semaphorin-1A | India Wildtype |
| NC_050203 | 68277837 | 4.395019565 | A | T | semaphorin-1A | India Wildtype |
| NC_050203 | 68326024 | 4.1296806 | C | T | semaphorin-1A | India Wildtype |
| NC_050203 | 68326239 | 4.015595301 | T | A | semaphorin-1A | India Wildtype |
| NC_050203 | 68326660 | 4.13409182 | C | G | semaphorin-1A | India Wildtype |
| NC_050203 | 68486013 | 4.011040322 | G | T | protein amalgam | India Wildtype |
| NC_050203 | 69675803 | -3.92786622 | G | A | serine/threonine-protein phosphatase 6 regulatory ankyrin repeat subunit B | India Wildtype |
| NC_050203 | 70020143 | 4.172351126 | A | G | protein vestigial | India Wildtype |
| NC_050203 | 70020775 | 4.06435559 | G | C | protein vestigial | India Wildtype |
| NC_050203 | 70045156 | 4.512557977 | G | A | protein vestigial | India Wildtype |
| NC_050203 | 70626220 | 4.058284855 | T | C | collagen alpha-1(IV) chain | India Wildtype |
| NC_050203 | 71044546 | 4.888435465 | C | A | villin-like protein quail | Pakistan Colony |
| NC_050203 | 71180473 | 4.565476081 | G | A | uncharacterized LOC118512606 | India Wildtype |
| NC_050203 | 71545914 | 3.98538441 | C | G | uncharacterized LOC118514290 | India Wildtype |
| NC_050203 | 71602688 | 4.106673112 | C | G | uncharacterized LOC118514290 | India Wildtype |
| NC_050203 | 71605109 | 3.895286407 | A | T | uncharacterized LOC118514290 | India Wildtype |
| NC_050203 | 71707504 | 3.920380019 | C | A | uncharacterized LOC118513467 | India Wildtype |
| NC_050203 | 71711399 | -3.941191941 | T | C | uncharacterized LOC118513467 | India Wildtype |
| NC_050203 | 71713532 | 4.139339295 | C | A | uncharacterized LOC118513467 | India Wildtype |
| NC_050203 | 71715225 | 4.007712289 | C | T | uncharacterized LOC118513467 | India Wildtype |
| NC_050203 | 71905136 | 3.907778677 | G | A | signal-induced proliferation-associated 1-like protein 2 | India Wildtype |
| NC_050203 | 71905998 | 3.937253087 | A | G | signal-induced proliferation-associated 1-like protein 2 | India Wildtype |
| NC_050203 | 71970111 | 3.903625662 | C | G | uncharacterized LOC118509884 | India Wildtype |
| NC_050203 | 73900909 | 3.969600934 | G | A | protein nubbin-like | Ethiopia |
| NC_050203 | 73902958 | 3.933156291 | G | A | protein nubbin-like | India Wildtype |
| NC_050203 | 74021939 | -4.114803599 | C | A | translation initiation factor 4E-binding protein Mextli | India Colony |
| NC_050203 | 74023549 | -4.386336954 | C | T | translation initiation factor 4E-binding protein Mextli | India Colony |
| NC_050203 | 74023551 | -4.386336954 | G | A | translation initiation factor 4E-binding protein Mextli | India Colony |
| NC_050203 | 74023555 | -4.198143659 | C | A | translation initiation factor 4E-binding protein Mextli | India Colony |
| NC_050203 | 74023558 | -4.198143659 | T | C | translation initiation factor 4E-binding protein Mextli | India Colony |
| NC_050203 | 74070694 | -3.993126659 | C | T | zinc finger protein 177-like | India Colony |
| NC_050203 | 75113873 | -4.113206643 | A | G | ankyrin repeat domain-containing protein 29 | India Colony |
| NC_050203 | 75295606 | 3.922949542 | A | C | ankyrin repeat domain-containing protein 29 | India Wildtype |
| NC_050203 | 76578337 | 4.099453434 | A | T | troponin C | Ethiopia |
| NC_050203 | 77207978 | -4.127207778 | G | A | mitogen-activated protein kinase-binding protein 1 | India Wildtype |
| NC_050203 | 78546249 | 3.950311979 | A | T | uncharacterized LOC118514662 | India Wildtype |
| NC_050203 | 79650863 | -4.007420444 | T | C | guanine nucleotide-binding protein-like 1 | India Wildtype |
| NC_050203 | 80108705 | -4.143333402 | G | A | cGMP-dependent 3',5'-cyclic phosphodiesterase-like | India Colony |
| NC_050203 | 80214728 | -3.951967008 | T | C | uncharacterized LOC118509173 | India Wildtype |
| NC_050203 | 81170754 | 4.435852329 | T | A | Shroom | India Wildtype |
| NC_050203 | 82021224 | -4.023829074 | T | A | potassium voltage-gated channel protein Shaw-like | India Wildtype |
| NC_050203 | 82589023 | 3.926169713 | A | G | uncharacterized LOC118513670 | India Colony |
| NC_050203 | 82589034 | 3.926169713 | C | A | uncharacterized LOC118513670 | India Colony |
| NC_050203 | 83093879 | 4.423419206 | A | G | myosin-2 heavy chain-like | India Wildtype |
| NC_050203 | 83104282 | 4.090037048 | T | G | protein spire | India Wildtype |
| NC_050203 | 83104729 | 3.977076153 | A | G | protein spire | India Wildtype |
| NC_050203 | 83110002 | 4.267969412 | T | C | protein spire | India Wildtype |
| NC_050203 | 83124905 | 4.087685281 | C | T | protein spire | India Wildtype |
| NC_050203 | 85472023 | -3.897116675 | T | A | WD repeat domain phosphoinositide-interacting protein 2-like | India Wildtype |
| NC_050203 | 85786629 | -3.926921353 | T | C | uncharacterized LOC118513953 | India Wildtype |
| NC_050203 | 85918612 | 3.950698384 | A | T | zwei Ig domain protein zig-8-like | India Wildtype |
| NC_050203 | 86547280 | 4.061766049 | T | C | protein madd-4 | India Wildtype |
| NC_050203 | 88069527 | -3.90973482 | A | T | phosphatase 1 regulatory subunit 14B | India Wildtype |
| NC_050203 | 88069539 | 3.97967158 | G | A | phosphatase 1 regulatory subunit 14B | India Wildtype |
| NC_050203 | 88090779 | -3.998271469 | C | T | inactive hydroxysteroid dehydrogenase-like protein 1 | India Wildtype |

**Supplementary Table S5.** *CYP* cluster locations for coverage-based detection of CNVs

| Chromosome | Start | End | Genes contained |
| --- | --- | --- | --- |
| NC_050202.1 | 2052235 | 2078445 | *CYP9f2 & CYP9e2* |
| NC_050202.1 | 46100775 | 47814861 | *CYP4d2, CYP10, CYP4d1, CYP4d2 & CYP4aa1* |
| NC_050202.1 | 62412107 | 62528720 | *CYP4c1, CYP4d2 & CYP4d14* |
| NC_050202.1 | 67473117 | 67501071 | *CYP6a22, CYP6a13, CYP6a14 & CYP6a2* |
| NC_050202.1 | 70224272 | 70335127 | *CYP4c1, CYP313b1 & CYP4c21* |
| NC_050203.1 | 78161225 | 79031149 | *CYP4c1, CYPb5, CYP4d8 & CYP4c1* |
| NC_050203.1 | 81910765 | 81987094 | *CYP6d3, CYP6a23, CYP6a14, CYP6a1 & CYP6a8* |

**Supplementary Table S6.** Coordinates for Candidate Genes

| Chr | Start Position | End Position | Gene |
| --- | --- | --- | --- |
| NC_050201.1 | 88726 | 102111 | cytochrome_P450_4c3 |
| NC_050201.1 | 106999 | 112127 | cytochrome_P450_4c3like |
| NC_050201.1 | 111481 | 119002 | cytochrome_P450_4c3like |
| NC_050201.1 | 1504398 | 1514674 | cytochrome_P450_307a1 |
| NC_050201.1 | 3560209 | 3564024 | cytochrome_P450_315a1,_mitochondrial |
| NC_050201.1 | 3732360 | 3739197 | cytochrome_P450_4g15 |
| NC_050201.1 | 4341245 | 4343391 | cytochrome_c_oxidase_subunit_7A,_mitochondriallike |
| NC_050201.1 | 8970310 | 8983348 | acetylcholinesterase |
| NC_050201.1 | 9696545 | 9701624 | probable_cytochrome_P450_9f2 |
| NC_050201.1 | 12234996 | 12238159 | cytochrome_P450_4g15like |
| NC_050201.1 | 13053874 | 13055979 | cytochrome_c_oxidase_subunit_6A,_mitochondrial |
| NC_050201.1 | 13597535 | 13612525 | gaba |
| NC_050201.1 | 13609056 | 13627740 | gaba |
| NC_050201.1 | 14678192 | 14682991 | cytochrome_P450_4d2like |
| NC_050202.1 | 2051735 | 2056124 | probable_cytochrome_P450_9f2 |
| NC_050202.1 | 2068365 | 2076761 | probable_cytochrome_P450_9f2 |
| NC_050202.1 | 2070363 | 2073916 | probable_cytochrome_P450_9f2 |
| NC_050202.1 | 2075922 | 2078945 | cytochrome_P450_9e2like |
| NC_050202.1 | 6548074 | 6549882 | cytochrome_c_oxidase_subunit_5A,_mitochondrial |
| NC_050202.1 | 9611539 | 9614468 | probable_cytochrome_P450_6a13 |
| NC_050202.1 | 10067003 | 10068981 | cytochrome_c_oxidase_assembly_protein_COX19 |
| NC_050202.1 | 10592571 | 10595110 | cytochrome_c_oxidase_assembly_factor_7_homolog |
| NC_050202.1 | 11810265 | 11812951 | cytochrome_c_oxidase_assembly_factor_3,_mitochondrial |
| NC_050202.1 | 12456416 | 12458396 | cytochrome_bc1_complex_subunit_7like |
| NC_050202.1 | 12866820 | 12868684 | cytochrome_c_oxidase_assembly_factor_4_homolog,_mitochondrial |
| NC_050202.1 | 14420568 | 14476837 | gaba |
| NC_050202.1 | 14979070 | 15031128 | cytochrome_b5_reductase_4 |
| NC_050202.1 | 16350769 | 16354427 | probable_cytochrome_P450_6g2 |
| NC_050202.1 | 16412717 | 16418405 | probable_cytochrome_P450_6g2 |
| NC_050202.1 | 21954473 | 21956155 | cytochrome_c_oxidase_subunit_6C1like |
| NC_050202.1 | 33131911 | 33133558 | cytochrome_c_oxidase_assembly_factor_5 |
| NC_050202.1 | 34776441 | 34778411 | cytochrome_c_oxidase_assembly_protein_COX11,_mitochondrial |
| NC_050202.1 | 35626431 | 35634925 | cytochrome_P450_4c3like |
| NC_050202.1 | 36409879 | 36415240 | acetylcholinesterase |
| NC_050202.1 | 37052015 | 37053808 | cytochrome_bc1_complex_subunit_8 |
| NC_050202.1 | 38366395 | 38406189 | cytochrome_P450_306a1 |
| NC_050202.1 | 38404739 | 38430214 | cytochrome_P450_18a1 |
| NC_050202.1 | 41412380 | 41413863 | cytochrome_c_oxidase_assembly_factor_6_homolog |
| NC_050202.1 | 43175047 | 43223404 | gaba |
| NC_050202.1 | 46100275 | 46105912 | cytochrome_P450_4d2like |
| NC_050202.1 | 46104008 | 46107054 | cytochrome_P450_4d2like |
| NC_050202.1 | 46109612 | 46116799 | cytochrome_P450_10like |
| NC_050202.1 | 46115989 | 46119138 | cytochrome_P450_4d1like |
| NC_050202.1 | 46119632 | 46123608 | cytochrome_P450_4d2like |
| NC_050202.1 | 47808045 | 47815361 | probable_cytochrome_P450_4aa1 |
| NC_050202.1 | 49187051 | 49188500 | cytochrome_ctype_heme_lyase |
| NC_050202.1 | 51904965 | 51907145 | cytochrome_c_oxidase_subunit_NDUFA4 |
| NC_050202.1 | 59231801 | 59234411 | cytochrome_P450_4C1like |
| NC_050202.1 | 60903514 | 60973523 | ace1 |
| NC_050202.1 | 62411607 | 62415381 | cytochrome_P450_4d2like |
| NC_050202.1 | 62414465 | 62417489 | probable_cytochrome_P450_4d14 |
| NC_050202.1 | 62525995 | 62529220 | cytochrome_P450_4d2like |
| NC_050202.1 | 63165254 | 63167245 | cytochrome_bc1_complex_subunit_6,_mitochondrial |
| NC_050202.1 | 66806173 | 66809428 | cytochrome_b5like |
| NC_050202.1 | 67472617 | 67478060 | probable_cytochrome_P450_6a14 |
| NC_050202.1 | 67482571 | 67485276 | probable_cytochrome_P450_6a13 |
| NC_050202.1 | 67485689 | 67488495 | probable_cytochrome_P450_6a14 |
| NC_050202.1 | 67487948 | 67491312 | probable_cytochrome_P450_6a14 |
| NC_050202.1 | 67491869 | 67494776 | cytochrome_P450_6a2like |
| NC_050202.1 | 67493777 | 67496995 | cytochrome_P450_6a2like |
| NC_050202.1 | 67496203 | 67499144 | probable_cytochrome_P450_6a14 |
| NC_050202.1 | 67498607 | 67501571 | cytochrome_P450_6a2like |
| NC_050202.1 | 69227289 | 69230377 | cytochrome_P450_4C1like |
| NC_050202.1 | 70223772 | 70229983 | cytochrome_P450_4C1like |
| NC_050202.1 | 70230835 | 70233782 | probable_cytochrome_P450_313b1 |
| NC_050202.1 | 70235014 | 70239471 | cytochrome_P450_4C1like |
| NC_050202.1 | 70315918 | 70322279 | cytochrome_P450_4c21like |
| NC_050202.1 | 70329703 | 70332676 | cytochrome_P450_4c21like |
| NC_050202.1 | 70332411 | 70335627 | cytochrome_P450_4c21like |
| NC_050202.1 | 73571925 | 73575794 | cytochrome_P450_9c1like |
| NC_050202.1 | 75038337 | 75041512 | cytochrome_c_oxidase_assembly_protein_COX15_homolog |
| NC_050202.1 | 76074873 | 76077996 | cytochrome_P450_6d3like |
| NC_050202.1 | 79425406 | 79428574 | probable_cytochrome_P450_304a1 |
| NC_050202.1 | 79429061 | 79431988 | probable_cytochrome_P450_304a1 |
| NC_050202.1 | 79431030 | 79433933 | probable_cytochrome_P450_304a1 |
| NC_050202.1 | 84283143 | 84365882 | cytochrome_P450_4c21like |
| NC_050202.1 | 86892946 | 86896657 | gaba |
| NC_050203.1 | 958500 | 960230 | cytochrome_c_oxidase_subunit_7C,_mitochondriallike |
| NC_050203.1 | 2375839 | 2379041 | probable_cytochrome_P450_308a1 |
| NC_050203.1 | 7250172 | 7253445 | cytochrome_bc1_complex_subunit_2,_mitochondrial |
| NC_050203.1 | 7689427 | 7699187 | probable_cytochrome_P450_301a1,_mitochondrial |
| NC_050203.1 | 7857762 | 7859720 | cytochrome_b5like |
| NC_050203.1 | 8125129 | 8130922 | probable_cytochrome_P450_4ac1 |
| NC_050203.1 | 8127942 | 8130922 | cytochrome_P450_4C1like |
| NC_050203.1 | 8130030 | 8133705 | probable_cytochrome_P450_4ac1 |
| NC_050203.1 | 9121560 | 9127446 | cytochrome_P450_302a1,_mitochondrial |
| NC_050203.1 | 10974686 | 10989205 | probable_cytochrome_P450_49a1 |
| NC_050203.1 | 12124390 | 12128533 | cytochrome_c1,_heme_protein,_mitochondrial |
| NC_050203.1 | 20105280 | 20108797 | probable_cytochrome_P450_305a1 |
| NC_050203.1 | 20115297 | 20118162 | probable_cytochrome_P450_305a1 |
| NC_050203.1 | 20117300 | 20122574 | probable_cytochrome_P450_305a1 |
| NC_050203.1 | 20464294 | 20467220 | cytochrome_c_oxidase_assembly_protein_COX18,_mitochondrial |
| NC_050203.1 | 21704546 | 21712430 | cytochrome_b_reductase_1 |
| NC_050203.1 | 24899015 | 24903457 | cytochrome_b5 |
| NC_050203.1 | 34257949 | 34263449 | cytochrome_c_oxidase_subunit_6B1 |
| NC_050203.1 | 39837238 | 39848504 | cytochrome_b_reductase_1 |
| NC_050203.1 | 42804385 | 42848676 | para |
| NC_050203.1 | 43723261 | 43729531 | gaba |
| NC_050203.1 | 45871461 | 45875638 | cytochrome_b5related_protein |
| NC_050203.1 | 45880580 | 45889660 | cytochrome_b5related_proteinlike |
| NC_050203.1 | 46667407 | 46671592 | probable_cytochrome_P450_303a1 |
| NC_050203.1 | 47395205 | 47397305 | cytochrome_b5_domaincontaining_protein_1 |
| NC_050203.1 | 47490751 | 47494415 | cytochrome_b561_domaincontaining_protein_2like |
| NC_050203.1 | 59096598 | 59099572 | cytochrome_bc1_complex_subunit_Rieske,_mitochondrial |
| NC_050203.1 | 61151438 | 61153106 | cytochrome_c_oxidase_subunit_6A2,_mitochondriallike |
| NC_050203.1 | 66772111 | 66774579 | cytochrome_c_oxidase_subunit_4_isoform_1,_mitochondriallike |
| NC_050203.1 | 66854320 | 66856654 | cytochrome_c_oxidase_subunit_5B,_mitochondriallike |
| NC_050203.1 | 67922093 | 67936616 | cytochrome_P450_307a1like |
| NC_050203.1 | 70579640 | 70582664 | GSTe2 |
| NC_050203.1 | 73200956 | 73203966 | cytochrome_c2 |
| NC_050203.1 | 73484513 | 73486309 | cytochrome_c_oxidase_subunit_7A1,_mitochondrial |
| NC_050203.1 | 73725667 | 73738374 | gaba |
| NC_050203.1 | 76240375 | 76248445 | probable_cytochrome_P450_9f2 |
| NC_050203.1 | 78160725 | 78165154 | cytochrome_P450_4C1like |
| NC_050203.1 | 78316505 | 78320067 | cytochrome_P450_4C1like |
| NC_050203.1 | 78337768 | 78342108 | cytochrome_P450_4C1like |
| NC_050203.1 | 78814452 | 78817922 | cytochrome_b5like |
| NC_050203.1 | 78961810 | 78964713 | cytochrome_P450_4d8like |
| NC_050203.1 | 78977278 | 78980660 | cytochrome_P450_4c3like |
| NC_050203.1 | 79028214 | 79031649 | cytochrome_P450_4d1like |
| NC_050203.1 | 81910265 | 81912975 | cytochrome_P450_6d3like |
| NC_050203.1 | 81912673 | 81915454 | cytochrome_P450_6d3like |
| NC_050203.1 | 81917206 | 81919987 | cytochrome_P450_6d3like |
| NC_050203.1 | 81957872 | 81960753 | probable_cytochrome_P450_6a23 |
| NC_050203.1 | 81959809 | 81962640 | probable_cytochrome_P450_6a14 |
| NC_050203.1 | 81962588 | 81965369 | probable_cytochrome_P450_6a23 |
| NC_050203.1 | 81966113 | 81968994 | cytochrome_P450_6A1like |
| NC_050203.1 | 81968548 | 81971205 | probable_cytochrome_P450_6a14 |
| NC_050203.1 | 81971965 | 81974900 | probable_cytochrome_P450_6a14 |
| NC_050203.1 | 81974104 | 81976806 | probable_cytochrome_P450_6a14 |
| NC_050203.1 | 81976334 | 81979241 | cytochrome_P450_6a8like |
| NC_050203.1 | 81982844 | 81985834 | cytochrome_P450_6A1like |
| NC_050203.1 | 81984904 | 81987594 | probable_cytochrome_P450_6a13 |
| NC_050203.1 | 84560108 | 84563683 | cytochrome_P450_CYP12A2like |
| NC_050203.1 | 84563163 | 84566339 | cytochrome_P450_CYP12A2like |
| NC_050203.1 | 86066358 | 86068161 | cytochrome_c_oxidase_subunit_6Clike |
| NC_050203.1 | 87999243 | 88000882 | cytochrome_c_oxidase_assembly_protein_COX20,_mitochondrial |

**Supplementary Table S7.** Accession numbers for publicly available raw data used in this study

| **Accession Number** | **Population** |
| --- | --- |
| SRR1168951 | India |
| SRR14657487 | India_Colony |
| SRR14657489 | India_Colony |
| SRR14657492 | India_Colony |
| SRR14657499 | India_Colony |
| SRR14657502 | India_Colony |
| SRR15146350 | India |
| SRR15257906 | India_Bangalore |
| SRR15257907 | India_Bangalore |
| SRR15257908 | India_Bangalore |
| SRR15257909 | India_Bangalore |
| SRR15257910 | India_Bangalore |
| SRR15257911 | India_Bangalore |
| SRR15257912 | India_Bangalore |
| SRR15257913 | India_Bangalore |
| SRR15257914 | India_Bangalore |
| SRR15257915 | India_Bangalore |
| SRR15293885 | India_Mangalore |
| SRR15293886 | India_Mangalore |
| SRR15293887 | India_Mangalore |
| SRR15293888 | India_Mangalore |
| SRR15293889 | India_Mangalore |
| SRR15293890 | India_Mangalore |
| SRR15293892 | India_Mangalore |
| SRR15293893 | India_Mangalore |
| SRR15293894 | India_Mangalore |
| SRR15311902 | India_Colony |
| SRR15311908 | India_Colony |
| SRR15400245 | India_Colony |
| SRR15400250 | India_Colony |
| SRR15507619 | India_Colony |
| SRR15507622 | India_Colony |
| SRR15533963 | India_Colony |
| SRR15533968 | India_Colony |
| SRR15533971 | India_Colony |
| SRR15603373 | India_Colony |
| SRR630084 | PakistanSDA500 |
| SRR643416 | India_Colony |
| SRR850082 | PakistanSDA500 |
| SRR850083 | PakistanSDA500 |
| SRR850084 | PakistanSDA500 |
| SRR850085 | PakistanSDA500 |
| SRR850086 | PakistanSDA500 |

**Supplementary Figure S1.** Sliding window analysis of nucleotide diversity in *An. stephensi* by chromosome.

Calculated across populations in 100kb windows, (pi) represents the average number of nucleotide differences per site. Sliding window analysis generated using vcftools and visualised in R.

A)

A)


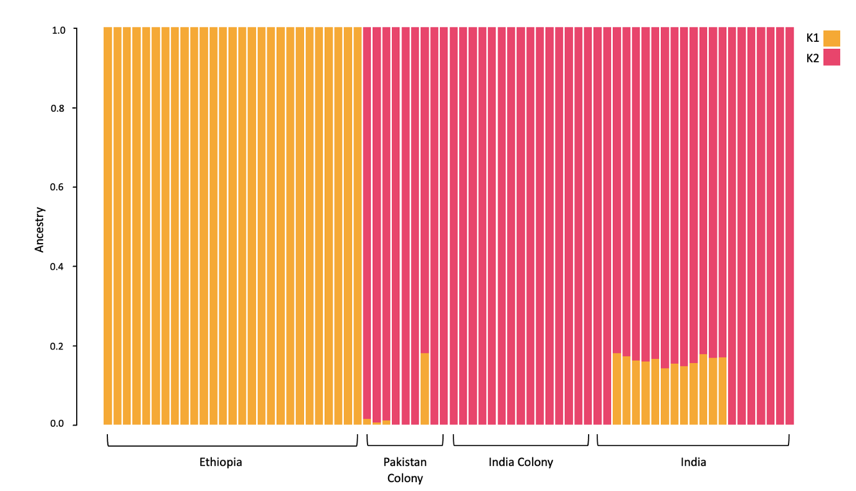


B)


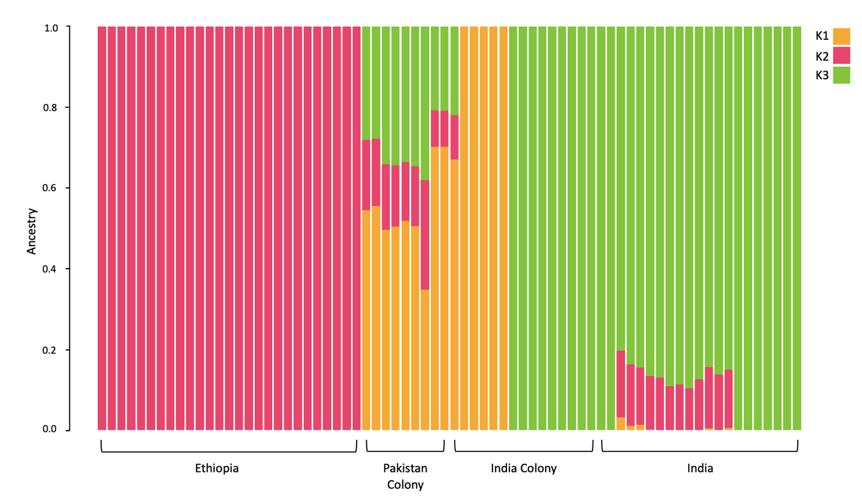


C)


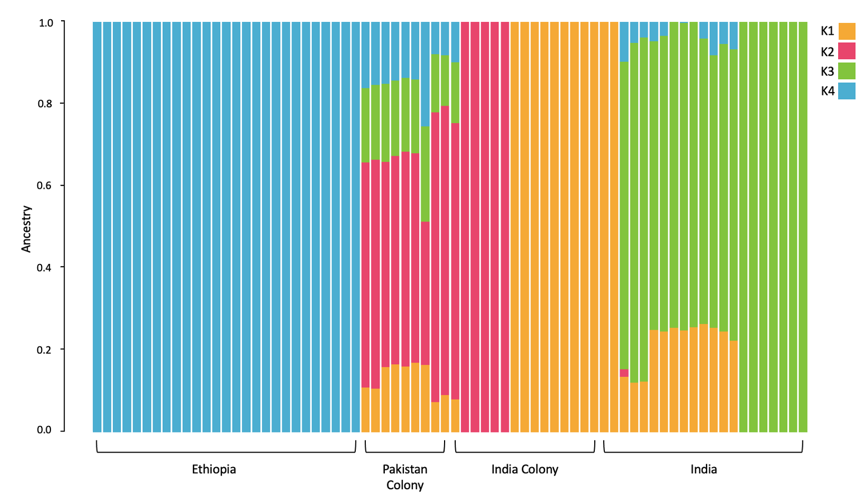


**Supplementary Figure S2.** **Genome wide admixture analysis of *An. stephensi* isolates**

Each isolate is represented by a column. A) K=2, B) K=3, C) K=4 ancestral populations, across the four isolate groups (Ethiopia (n= 27), Indian colony (n=21), Indian field (n=16), and Pakistan colony (n=8)) analyses.


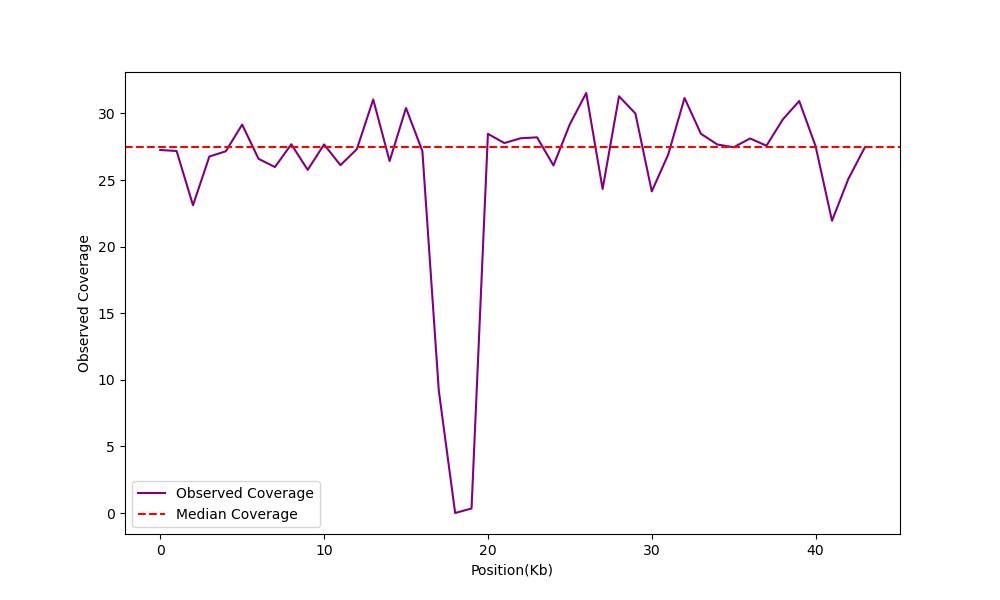

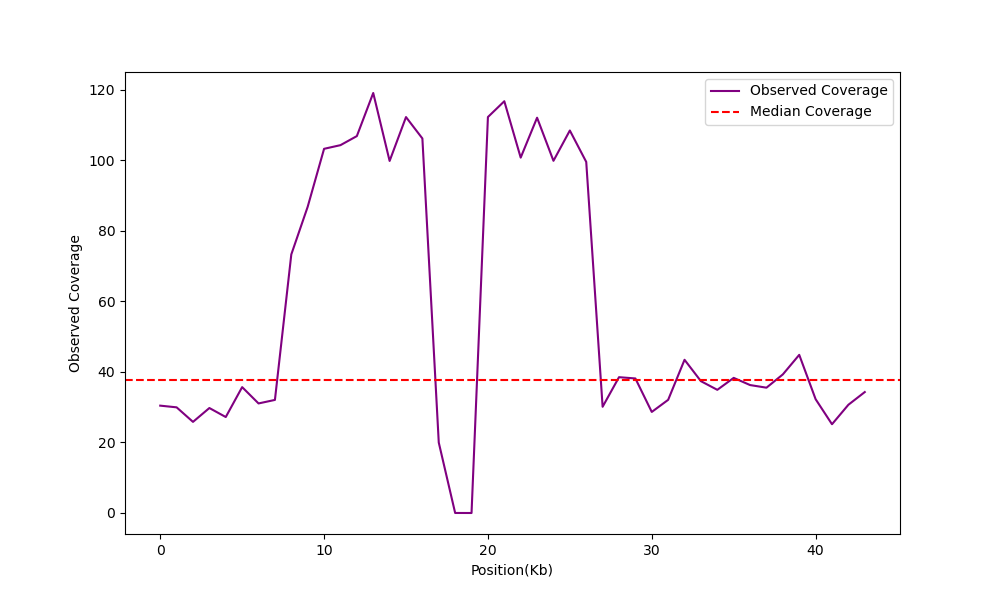

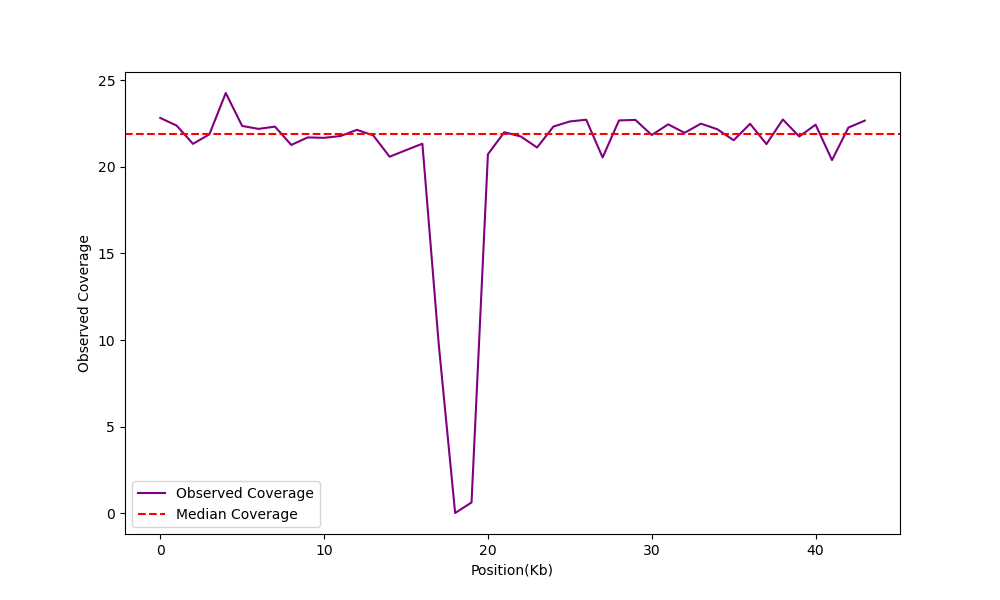

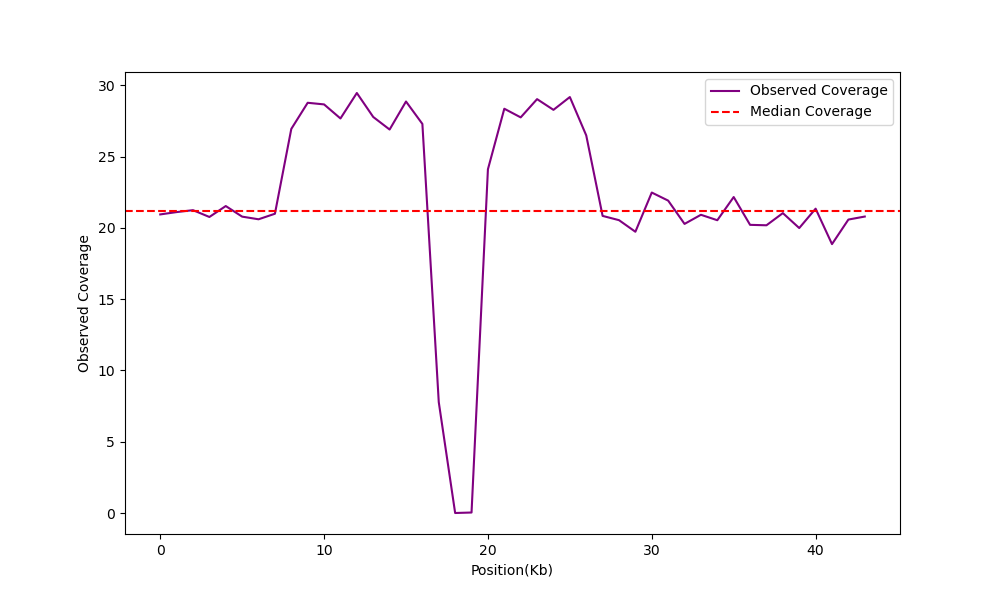


A

B

C

D

**Supplementary Figure S3.** Observed coverage for *CYP6a* gene cluster on Chromosome 2 (67473117-67501071) in comparison to genome wide median for each population. A) Ethiopia, B) Indian Colony, C) Indian field, and D) Pakistan Colony.
